# Rational Design, Synthesis, and Anti-Proliferative Evaluation of Novel 4-Aryl-3,4-Dihydro-2*H*-1,4-Benzoxazines

**DOI:** 10.3390/molecules29010166

**Published:** 2023-12-27

**Authors:** Xiaoming Fu, Daniel Wenholz, Daniel S. H. Chan, David StC. Black, Naresh Kumar

**Affiliations:** School of Chemistry, University of New South Wales (UNSW), Kensington, Sydney 2052, Australiad.black@unsw.edu.au (D.S.B.)

**Keywords:** 1,4-benzoxazines, heterocyclic, anticancer, structure activity relationship

## Abstract

A synthetic pathway to a novel 4-aryl-3,4-dihydro-2*H*-1,4-benzoxazine scaffold was developed and a series of compounds based on the scaffold were synthesised as potential anticancer agents. The 4-aryl-substituted compounds were prepared via Buchwald–Hartwig cross-coupling between substituted bromobenzenes and various 1,4-benzoxazines, which in turn were generated from a cascade hydrogenation and reductive amination one-pot reaction. These analogues exhibited moderate to good potency against various cancer cell lines. Structure–activity relationship analysis indicated that the inclusion of hydroxyl groups on ring A and ring B was beneficial to biological activity, while having a *para*-amino group on ring C significantly enhanced potency. Molecule **14f** displayed the most potent anticancer activity (IC_50_ = 7.84–16.2 µM against PC-3, NHDF, MDA-MB-231, MIA PaCa-2, and U-87 MG cancer cell lines), indicating its potential as a lead compound for further structural optimisation. All the synthesised compounds were fully characterised with NMR, HMRS, and IR. The novel benzoxazine scaffold described in this study holds promise and deserves further in-depth studies.

## 1. Introduction

Cancer is one of the leading causes of death worldwide [1]. In 2019, over 1.8 million new cancer cases were diagnosed in the United States [2]. Lung, colorectal, breast, and prostate cancer are the most frequent forms of cancer which contribute to around 30% of all new diagnoses and at least 10% of cancer-related deaths [3].

Conventional approaches for modern drug development heavily rely on the discovery of active compounds against an individual biological target. However, such single-target methods are not always effective at delivering drugs against complex multifactorial diseases such as cancer [4]. To overcome this limitation, one strategy is to design multitarget drugs that are capable of simultaneously and specifically binding to multiple sites within the target system [5,6].

In an effort toward developing novel pharmacophores as anticancer agents, the synthetic *cis*- and *trans*-isoflavanols have attracted much attention [7]. Equol, a molecule possessing a benzopyran pharmacophore, displays low-nanomolar binding affinity to 5α-DHT and reduces the risk of developing prostate-associated disorders [8]. A series of 4-substituted isoflavonoids demonstrated potential pharmacological value by binding avidly to the estrogen receptor [9,10]. After iterative chemical modification, 4-substituted benzopyran compounds demonstrated a progressive increase in broad anticancer activity which was attributed to their potential role as tubulin polymerization inhibitors leading to delayed mitosis as a result [11]. These findings suggest that 4-substituted isoflavan is a promising pharmacophore with broad anticancer potential.

Benzoxazine analogues, particularly 1,4-benzoxazine systems, represent another important class of biologically active compounds with significant pharmaceutical value against various diseases, including cardiovascular disorders, neurodegeneration, inflammation, and cancer [12,13,14]. Triazole–benzoxazine hybrids also displayed promising anti-proliferative activities [15]. A 1,4-benzoxazine inhibited hypoxic tumours by downregulating hypoxia-induced genes while only exhibiting low toxicity to normoxic cells [16]. 2,3-Dihydro-1,4-benzoxazines were investigated as orally bioavailable anticancer agents via inhibition of angiogenesis [17]. Another type of 2,3-dihydro-1,4-benzoxazine possessed a dual antiangiogenic mechanism via both thrombin and integrin inhibitory activity [18]. A 1,4-benzoxazine sulphonamide was a potent agonist of retinoic acid receptor-related orphan receptor (ROR) and decreased cancer growth via increasing T-cell activity in the tumour microenvironment [19]. 1,4-Benzoxazin-3-one sulphonamides were evaluated as inhibitors of PI3Ka in several cancer cell lines [20,21]. Recently, a tyrosine-based benzoxazine was discovered to show promising anticancer activity via the inducement of breast cancer cell apoptosis [22]. These findings suggest that the 1,4-benzoxazine scaffold is biologically privileged for the development of anticancer agents.

**Figure 1:**
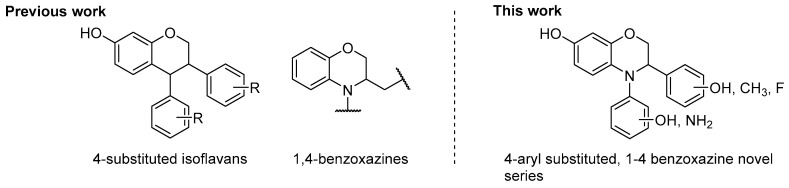
Design of 4-aryl substituted 1,4-benzoxazines as anticancer agents.

Due to the similarity in the scaffolds of 4-substituted isoflavans and 3,4-dihydro-2*H*-1,4-benzoxazines, we hypothesized that novel 4-aryl substituted 1,4-benzoxazines might display improved toxicity and selectivity against cancer cell lines (Figure 1). In this paper, we designed, synthesised, and examined the in vitro biological activities of a small library of 1,4-benzoxazine derivatives. The reported synthetic path is efficient and enables various modifications so that novel analogues can be easily prepared. The structure–activity relationships (SAR) of these compounds provide guidance to the development of future lead candidates against cancer.

## 2. Results and Discussion

### 2.1. Chemical Synthesis and Characterization

**Scheme 1:**
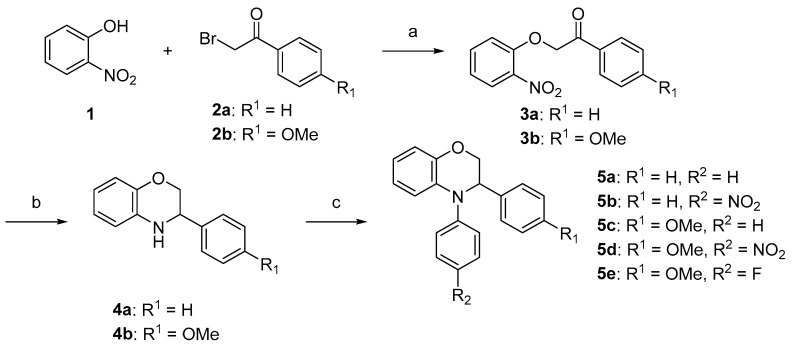
Reagents and conditions: (**a**) K_2_CO_3_, acetone, rt, 6 h, N_2_, 72–83%; (**b**) H_2_, Pd/C, methanol, rt, 12 h, 50–95%; and (**c**) substituted bromobenzene, Pd_2_(dba)_3_, XPhos, CsCO_3_, *tert*-butanol, toluene, 100 °C, 16 h, argon, 23–50%.

The methodology for synthesising the benzoxazine scaffold was based on a previously published patent, with some modifications [23]. Notably, the original patent did not detail the incorporation of 4-aryl substitution. The reaction of 2-nitrophenol **1** and 2-bromoacetophenone **2a** and 2-bromo-4′-methoxyacetopheone **2b** under Williamson ether synthesis conditions provided the nitro intermediates **3a** and **3b** in good yield (Scheme 1). The nitro groups of **3a** and **3b** were reduced to amines using Pd/C (as opposed to NaH_2_PO_2_) [23], which was followed by intramolecular cyclization via the Mannich reaction to give the 1,4-benzoxazines **4a** and **4b** in a one-pot reaction. Finally, *N*-arylation was performed under modified Buchwald–Hartwig amination conditions with excess substituted bromobenzene to generate the 4-aryl-3,4-dihydro-2*H*-1,4-benzoxazine derivatives **5a**–**e** in moderate yields of 23–50%.

**Scheme 2:**
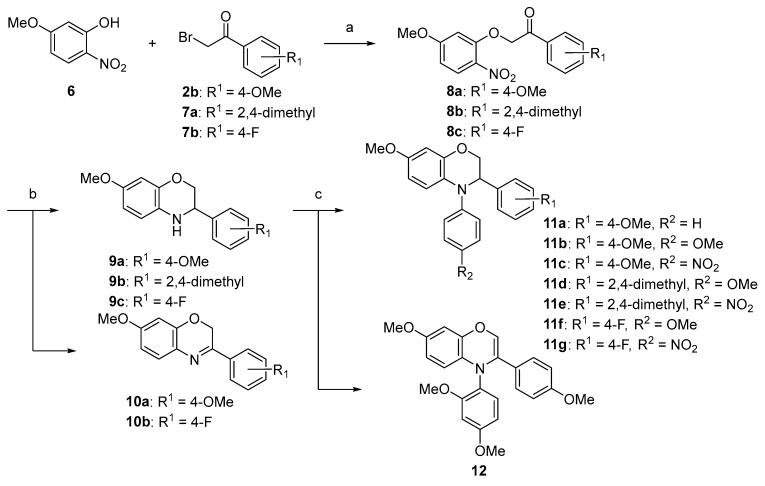
Reagents and conditions: (**a**) K_2_CO_3_, acetone, rt, 6 h, N_2_, 62–90%; (**b**) H_2_, Pd/C, methanol, rt, 12 h, 50–60%; and (**c**) substituted bromobenzene, Pd_2_(dba)_3_, XPhos, CsCO_3_, *tert*-butanol, toluene, 100 °C, 16 h, argon, 24–82%.

With the synthesis methodology in hand, we set out to further explore the biological activities of benzoxazines by introducing a 7-methoxy group to the benzoxazine core as well as different substituents on the pendant aromatic ring, and to explore further the biological activities of these benzoxazines. Thus, 5-methoxy-2-nitrophenol **6** was alkylated with various 2-bromoacetophenones **2b**, **7a**, **7b** to provide nitro compounds **8a**–**c** in 73–90% yield (Scheme 2). The intermediates were subjected to catalytic hydrogenation and intramolecular cyclization to furnish **9a**–**c**. Interestingly, small amounts of 2*H*-benzoxazines **10a** and **10b** were also isolated in addition to the main dihydrobenzoxazines **9a** and **9c** respectively. Different substitutions were introduced to the pendant 4-aryl group by reacting **9a**–**c** with various substituted bromobenzenes under similar reaction conditions, furnishing the 4-aryl-3,4-dihydro-2*H*-1,4-benzoxazine compounds **11a**–**g** in 24–82% yields. When **9a** was coupled with 1-bromo-2,4-dimethoxybenzene in similar conditions, the unsaturated benzoxazine **12** was the only product of the coupling reaction and the anticipated dihydrobenzoxazine was not formed. The variation on the benzoxazine core was evidenced by the absence of the two doublets at around 4.2 ppm corresponding to the protons of methylene at C2 and one triplet at around 5.0 ppm corresponding to the chiral proton of C3. The methylene protons are diastereotopic due to the presence of an adjacent chiral proton which makes them chemically non-equivalent, thus giving rise to two different chemical shifts. The structure was further confirmed by the presence of a singlet at 6.69 ppm integrating for one proton corresponding to the olefinic proton H2 in **12**.

**Scheme 3:**
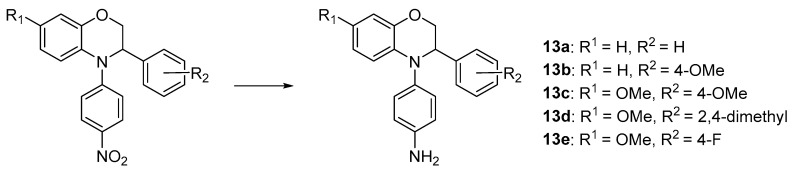
Reagents and conditions: H_2_, Pd/C, methanol, rt, 12 h, 55–100%.

Next, we wanted to evaluate the effect of amino substitution on the biological properties of the scaffold. Thus, various nitro benzoxazine derivatives were reduced by H_2_ in the presence of Pd/C in methanol to furnish the amine compounds **13a**–**e** (Scheme 3).

**Scheme 4:**
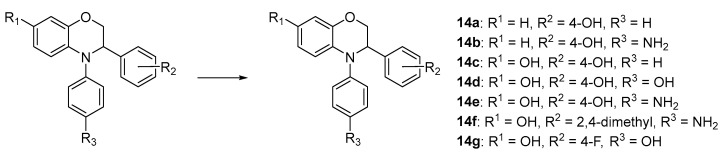
Reagents and conditions: BBr_3_, DCM, rt, 8 h, N_2_, 40–100%.

In search of improved bioactivities for the novel benzoxazines, we further modified the analogues to investigate the effect of having hydroxyl groups in the structure. Various methoxyl benzoxazines were demethylated in the presence of BBr_3_ in dichloromethane to afford the hydroxyl derivatives **14a**–**g** in good yields (Scheme 4).

**Scheme 5:**
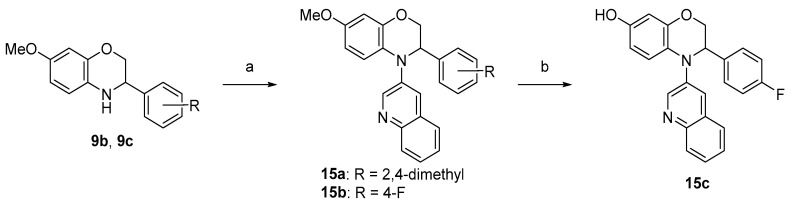
Reagents and conditions: (**a**) 3-bromoquinoline, Pd_2_(dba)_3_, Xphos, CsCO_3_, *tert*-butanol, toluene, 100 °C, 16 h, argon, 55%; (**b**) BBr_3_, DCM, rt, 8 h, N_2_, 89%.

The effect of heterocycle substitution was another direction we aimed to explore. Analogues **15a** and **15b** were prepared by coupling benzoxazines **9b** and **9c** with 3-bromoquinoline (Scheme 5). Compound **15b** was further demethylated to obtain the hydroxyl compound **15c**.

### 2.2. In Vitro Cancer Cell Proliferation Inhibitory Activities and Structure–Activity Relationship Study

To assess the cell growth inhibitory effect of the synthesised 1,4-dihydrobenzoxazine derivatives, their effects on cell proliferation were tested against MIA (PaCa-2) pancreatic cancer cells, (MDA-MB-231) breast cancer cells, and (PC-3) prostate cancer cells using an in vitro CellTiter-Glo assay (Table 1). Out of the 29 synthesised molecules, **11a**, **13c**, **13d**, **14a**, **14b**, **14c**, **14d**, **14f**, and **15c** showed >50% activity against MIA PaCa-2 at 25 µM. Compounds **5c**, **5e**, **11a**, and **14c** displayed >40% growth inhibition against MDA-MB-231. For PC-3 cell line, compounds **13c**, **13d**, **14a**, **14f** displayed >25% activity while **14a** and **14f** displayed activity against both MDA-MB-231 and PC-3 cell lines.

**Figure 2:**
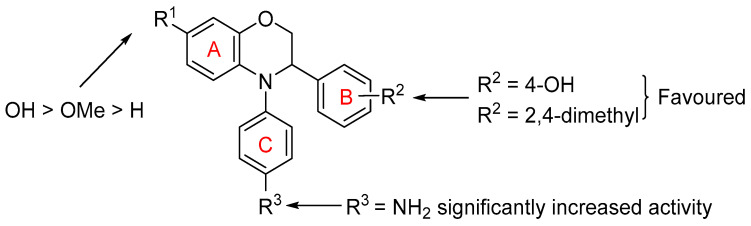
SAR conclusions of the designed 4-aryl-3,4-dihydro-2*H*-1,4-benzoxazine scaffold.

Based on the screening data of the novel 4-aryl-3,4-dihydro-2*H*-1,4-benzoxazines against three cancer cell lines, preliminary SAR conclusions could be drawn (Figure 2). Molecules with OMe (**11a**–**g**) are favoured over H (**5a**–**e**) on the C7-position of ring A, suggesting that electron-donating substitution on R^1^ improved activities. For R^2^ substitution on ring B, the introduction of 4-F, 4-OMe, and 2,4-dimethyl (**5c**–**e** and **11a**–**g**) lead to general antiproliferative improvements when compared to an unsubstituted ring (**5a** and **5b**). Among these three substitutions, compounds with 4-OMe (**11a** and **11b**, 91% and 44% against MIA PaCa-2, respectively) and 2,4-dimethyl (**11d**, 48%) were favoured over 4-F (**11f** and **11g**, 33% and 12%). These results suggested that the introduction of electron-donating groups at the *para*-position on ring B gave rise to stronger anti-cancer activities than electron-withdrawing *para*-substituents. Meanwhile, no significant difference was observed between 4-OMe and 2,4-dimethyl substituents, suggesting that the anticancer activities might be independent of the strength of the electron-donating groups. However, it should be noted that the introduction of an extra electron-donating methyl group at the 2-position could influence the overall anticancer activity of the compound. The demethylated analogues (**14a**–**g**) displayed superior efficacy over their methylated precursors (**13b**–**e**), suggesting that the presence of hydroxyl groups is beneficial for anticancer activity, with the exception being compound **14e**, which displayed no activity against all tested cell lines. The overall increased activity due to the presence of the hydroxyl groups could be attributed to hydrogen bonding interactions towards the potential binding site. This observation is supported by the binding of genistein, an isoflavone with two hydroxyl groups which allows it to bind to the estrogen receptors, thus exerting its effects against hormone-dependent cancers [24]. Compound **14f** with 4-NH_2_ at R^3^ exhibited significant improvement on inhibition against MDA-MB-231 (78%) and PC-3 (98%) cell lines when compared to other series, leading us to identify the 4-NH_2_ on ring C as a crucial substitution for increasing overall efficacy. It is hypothesized that this class of compounds exhibits anticancer activity with a mechanism similar to those of isoflavones. The precise mechanism of the anticancer activity remains to be explored.

**Figure 3:**
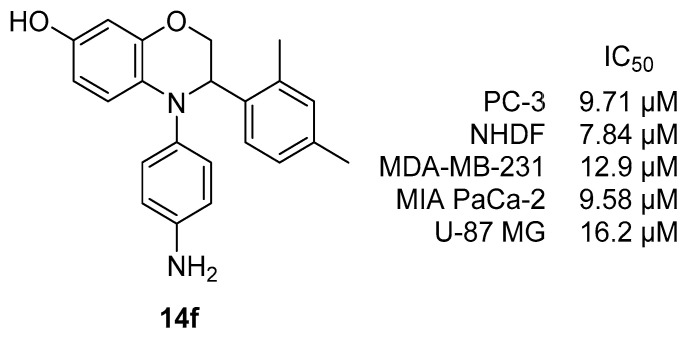
IC_50_ of molecule **14f** in cancer cell lines.

Compound **14f** (Figure 3) which exhibited 95% inhibition against MIA PaCa-2 and PC-3 and around 90% inhibition against the MDA-MB-231 cell line at 25 µM was selected as the lead compound to determine the IC_50_ values in the concentration range of 0.002–50 μM. The IC_50_ values were found to be 9.71, 7.84, 12.9, 9.58, and 16.2 μM corresponding to PC-3, NHDF, MDA-MB-231, MIA PaCa-2, and U-87 MG cancer cells, respectively. The IC_50_ values of a structurally similar anticancer agent Cantrixil were found to be 0.096, 3.72 and 0.205 μM for PC-3, MIA PaCa-2, and U-87 MG cancer cells, respectively [25]. The predicted physiochemical properties of the tested compounds were calculated using online software [26]. The significant parameters can be used as guidance to determine whether these compounds obey the Lipinski rule of five (description of the rule is provided in Table 2). The parameters of all the tested compounds fall within range of the Lipinski rule of five, suggesting these compounds potentially possess good oral bioavailability.

## 3. Materials and Methods

**General procedures**

All reagents and solvents were obtained from commercial sources and purified if necessary. Melting points were measured using a Mel-Temp melting point apparatus and are uncorrected. Melting points were measured using an OptiMelt melting point apparatus and are uncorrected. Infrared (IR) spectra were recorded using a Cary 630 FTIR spectrometer or Nicolet^TM^ iS^TM^ 10 FTIR spectrometer (Thermo Nicolet, Waltham, MA, USA) fitted with a diamond attenuated total reflectance (ATR) sample interface. ^1^H and ^13^C NMR spectra were obtained in the specified solvents on a Bruker Avance III HD 400 (Bruker, Sydney, NSW, Australia). Chemical shifts (δ) are in parts per million (ppm) internally referenced to the solvent nuclei. Multiplicities are assigned as singlet (s), broad singlet (bs), doublet (d), triplet (t), quartet (q), multiplet (m) or a combination of these (e.g., dd, dt, td), and coupling constants (*J*) are reported in Hertz (Hz). High-resolution mass spectrometry (HRMS) was performed using a Thermo LTQ Orbitrap XL instrument (Thermo Scientific, Waltham, MA, USA). ^1^H and ^13^C NMR spectra of the synthesised compounds are available in the Supplementary Materials. Thin-layer chromatography (TLC) was performed using 0.25 mm silica gel plates (60F-254). The products were purified by column chromatography on silica gel 60 (63–200 mesh).

**In vitro assay**

Cytotoxicity was determined using a CellTitre-Glo assay [27]. A stock solution of each compound was prepared at 10 mM in DMSO. IC_50_ values were determined by testing cell growth inhibition across 10 compound concentrations, starting at 50 μM and using 3-fold serial dilution in a 96-well plate. Each compound concentration was tested in duplicate in two independent experiments. DMSO final concentration was normalised at 0.5%. Plates were incubated for 72 h at 37 °C and 5% CO_2_. During data analysis, IC_50_ values were calculated with KLFit curve fitting software (version 5.5.0.5) using the 4 Parameter Logistic Model [fit = (A + ((B−A)/(1 + ((C/x)^D))))] (A: Bottom; B: Top; C: Relative IC_50_; D: Hill).

### Experimental Protocols and Analytical Details

**General procedure for the synthesis of 2-nitrophenoxyphenylethanones**

Anhydrous K_2_CO_3_ (1.8 eq.) was added to a stirred solution of the corresponding 2-nitrophenol in acetone at room temperature and stirred for 30 min. The corresponding 2-bromo-1-phenylethanone (1.1 eq.) was added to the reaction mixture and stirred at room temperature until the reaction showed signs of completion as indicated by TLC. The reaction mixture was filtered, and the residue was washed with acetone. The combined filtrate was concentrated in vacuo to obtain the crude product. Acetone was added followed by the slow addition of H_2_O until solid precipitated out. The solid thus obtained was filtered and dried to obtain the target 2-nitrophenoxyphenylethanone.

*2-(2-Nitrophenoxy)-1-phenylethan-1-one* (**3a**). Pale brown solid, yield: 83%. ^1^H NMR (400 MHz, DMSO-*d*_6_) δ 8.05–7.98 (m, 2H), 7.90 (dd, *J* = 8.1, 1.7 Hz, 1H), 7.76–7.67 (m, 1H), 7.63–7.54 (m, 3H), 7.30 (dd, *J* = 8.6, 1.1 Hz, 1H), 7.13 (m, 1H), 5.87 (s, 2H). ^13^C NMR (101 MHz, DMSO-*d*_6_) δ 194.06, 151.17, 140.15, 134.53, 134.46, 129.32, 128.38, 125.36, 121.38, 115.85, 71.54. Data consistent with reported literature [28].

*1-(4-Methoxyphenyl)-2-(2-nitrophenoxy)ethan-1-one* (**3b**). White solid, yield: 72%. ^1^H NMR (400 MHz, DMSO-*d*_6_) δ 8.03–7.94 (m, 2H), 7.89 (dd, *J* = 8.1, 1.7 Hz, 1H), 7.58 (ddd, *J* = 8.6, 7.4, 1.7 Hz, 1H), 7.25 (dd, *J* = 8.6, 1.1 Hz, 1H), 7.17–7.05 (m, 3H), 5.79 (s, 2H), 3.87 (s, 3H). ^13^C NMR (101 MHz, DMSO-*d*_6_) δ 192.32, 164.21, 151.28, 140.12, 134.51, 130.76, 127.41, 125.35, 121.29, 115.83, 114.57, 71.23, 56.14. Data consistent with reported literature [28].

*2-(5-Methoxy-2-nitrophenoxy)-1-(4-methoxyphenyl)ethan-1-one* (**8a**). Pale brown solid, yield: 62%, mp: 147–149 °C. IR (neat) 1681, 1290, 1172 cm^−1^. ^1^H NMR (400 MHz, DMSO-*d*_6_) δ 8.03–7.93 (m, 3H), 7.14–7.05 (m, 2H), 6.75 (d, *J* = 2.5 Hz, 1H), 6.69 (dd, *J* = 9.1, 2.5 Hz, 1H), 5.80 (s, 2H), 3.87 (s, 3H), 3.82 (s, 3H). ^13^C NMR (101 MHz, DMSO-*d*_6_) δ 192.26, 164.64, 164.19, 154.26, 133.21, 130.81, 128.23, 127.49, 114.55, 106.47, 101.69, 71.39, 56.63, 56.13. HRMS (+ESI): Found *m/z* 340.07910, [M + Na]^+^. C_16_H_15_NO_6_Na [340.07971].

*1-(2,4-Dimethylphenyl)-2-(5-methoxy-2-nitrophenoxy)ethan-1-one* (**8b**). Pale yellow solid, yield: 90%, mp: 124–126 °C. IR (neat) 2925, 1679, 1261 cm^−1^. ^1^H NMR (400 MHz, DMSO-*d*_6_) δ 7.97 (d, *J* = 9.1 Hz, 1H), 7.87 (d, *J* = 7.7 Hz, 1H), 7.19 (d, *J* = 8.9 Hz, 2H), 6.78–6.63 (m, 2H), 5.70 (s, 2H), 3.82 (s, 3H), 2.39 (s, 3H), 2.34 (s, 3H). ^13^C NMR (101 MHz, DMSO-*d*_6_) δ 196.66, 164.63, 154.19, 143.05, 138.76, 133.09, 131.94, 129.79, 128.22, 126.89, 106.68, 101.35, 72.42, 56.63, 21.45, 21.28. HRMS (+ESI): Found *m/z* 338.09992, [M + Na]^+^. C_17_H_17_NO_5_Na [338.10044].

*1-(4-Fluorophenyl)-2-(5-methoxy-2-nitrophenoxy)ethan-1-one* (**8c**). White solid, yield: 79%, mp: 173–175 °C. IR (neat) 1684, 1592, 1501 cm^−1^. ^1^H NMR (400 MHz, DMSO-*d*_6_) δ 8.15–8.05 (m, 2H), 7.99 (d, *J* = 9.1 Hz, 1H), 7.48–7.37 (m, 2H), 6.80 (d, *J* = 2.5 Hz, 1H), 6.70 (dd, *J* = 9.1, 2.5 Hz, 1H), 5.85 (s, 2H), 3.82 (s, 3H). ^13^C NMR (101 MHz, DMSO-*d*_6_) δ 192.64, 167.14, 164.68, 164.63, 154.10, 133.21, 131.58, 131.48, 131.41, 131.38, 128.24, 116.49, 116.27, 106.57, 101.72, 71.61, 56.66. HRMS (+ESI): Found *m/z* 328.05918, [M + Na]^+^. C_15_H_12_FNO_5_Na [328.05972].

**General procedure for the synthesis of 1,4-benzoxazines**

Palladium on carbon (10% wt, 0.1 eq) was added to a solution of the corresponding 2-nitrophenoxyphenylethanone in methanol at room temperature and stirred for 16 h. The reaction mixture was filtered through celite and the filtrate was concentrated in vacuo to obtain crude product, which was purified by flash chromatography (hexane/ethyl acetate) to obtain the target 1,4-benzoxazine.

*3-Phenyl-3,4-dihydro-2H-benzo[b][1,4]oxazine* (**4a**). Pale yellow oil, yield: 50%. ^1^H NMR (400 MHz, DMSO-*d*_6_) δ 7.49–7.26 (m, 5H), 6.78–6.66 (m, 3H), 6.59–6.47 (m, 1H), 6.26 (s, 1H), 4.47 (dt, *J* = 7.3, 2.4 Hz, 1H), 4.22 (ddd, *J* = 10.5, 3.1, 1.6 Hz, 1H), 3.90 (dd, *J* = 10.5, 7.5 Hz, 1H). ^13^C NMR (101 MHz, DMSO-*d*_6_) δ 142.70, 140.14, 134.92, 128.39, 127.63, 127.13, 121.19, 116.87, 115.74, 114.89, 69.91, 52.75. Data consistent with reported literature [29].

*3-(4-Methoxyphenyl)-3,4-dihydro-2H-benzo[b][1,4]oxazine* (**4b**). Pale yellow oil, yield: 96%. ^1^H NMR (400 MHz, DMSO-*d*_6_) δ 7.39–7.31 (m, 2H), 7.00–6.91 (m, 2H), 6.76–6.68 (m, 3H), 6.53 (ddd, *J* = 7.6, 5.2, 3.8 Hz, 1H), 6.17 (d, *J* = 1.7 Hz, 1H), 4.41 (dt, *J* = 7.5, 2.3 Hz, 1H), 4.19 (ddd, *J* = 10.4, 3.0, 1.7 Hz, 1H), 3.86 (dd, *J* = 10.4, 7.9 Hz, 1H), 3.76 (s, 3H). ^13^C NMR (101 MHz, DMSO-*d*_6_) δ 159.36, 143.19, 135.51, 132.36, 128.75, 121.58, 117.35, 116.19, 115.41, 114.29, 70.60, 55.57, 52.69. Data consistent with reported literature [30].

*7-Methoxy-3-(4-methoxyphenyl)-3,4-dihydro-2H-benzo[b][1,4]oxazine* (**9a**). Pale yellow solid, yield: 60%, mp: 147–149 °C. IR (neat) 3327, 2837, 1507 cm^−1^. ^1^H NMR (400 MHz, DMSO-*d*_6_) δ 7.39–7.30 (m, 2H), 6.98–6.90 (m, 2H), 6.69–6.56 (m, 1H), 6.40–6.31 (m, 2H), 5.74 (t, *J* = 1.8 Hz, 1H), 4.31 (dt, *J* = 8.1, 2.3 Hz, 1H), 4.16 (ddd, *J* = 10.4, 2.9, 1.8 Hz, 1H), 3.84 (dd, *J* = 10.4, 8.2 Hz, 1H), 3.75 (s, 3H), 3.63 (s, 3H). ^13^C NMR (101 MHz, DMSO-*d*_6_) δ 158.84, 151.65, 143.40, 131.91, 128.65, 128.31, 115.65, 113.79, 106.70, 102.11, 70.51, 55.27, 55.11, 52.33. HRMS (+ESI): Found *m/z* 272.12811, [M + H]+. C_16_H_18_NO_3_ [272.12867].

*3-(2,4-Dimethylphenyl)-7-methoxy-3,4-dihydro-2H-benzo[b][1,4]oxazine* (**9b**). White solid, yield: 95%, mp: 122–124 °C. IR (neat) 3348, 2872, 1504 cm^−1^. ^1^H NMR (400 MHz, DMSO) δ 7.31–7.24 (m, 1H), 7.06–6.99 (m, 2H), 6.65–6.58 (m, 1H), 6.39–6.31 (m, 2H), 5.61 (t, *J* = 1.9 Hz, 1H), 4.48 (dt, *J* = 8.2, 2.4 Hz, 1H), 4.18 (ddd, *J* = 10.6, 2.9, 2.0 Hz, 1H), 3.74 (dd, *J* = 10.5, 8.3 Hz, 1H), 3.63 (s, 3H), 2.33 (s, 3H), 2.25 (s, 3H). ^13^C NMR (101 MHz, DMSO) δ 151.64, 143.37, 136.30, 135.13, 134.64, 130.89, 128.96, 126.73, 126.70, 115.74, 106.70, 102.12, 69.19, 55.29, 49.24, 20.57, 18.66. HRMS (+ESI): Found *m/z* 270.14885, [M + H]^+^. C_17_H_20_NO_2_ [270.14940].

*3-(4-Fluorophenyl)-7-methoxy-3,4-dihydro-2H-benzo[b][1,4]oxazine* (**9c**). Brown solid: 92%, mp: 122–124 °C. IR (neat) 3363, 2860, 1507 cm^−1^. ^1^H NMR (400 MHz, DMSO-*d*_6_) δ 7.51–7.41 (m, 2H), 7.26–7.15 (m, 2H), 6.63 (dt, *J* = 8.6, 1.2 Hz, 1H), 6.40–6.33 (m, 2H), 5.84 (t, *J* = 1.8 Hz, 1H), 4.40 (dt, *J* = 7.9, 2.4 Hz, 1H), 4.19 (ddd, *J* = 10.5, 2.9, 1.7 Hz, 1H), 3.87 (dd, *J* = 10.4, 7.8 Hz, 1H), 3.64 (s, 3H). ^13^C NMR (101 MHz, DMSO-*d*_6_) δ 163.32, 160.90, 152.19, 143.88, 136.86, 136.84, 129.65, 129.57, 128.84, 116.17, 115.68, 115.47, 107.32, 102.63, 70.68, 55.74, 52.62. HRMS (+ESI): Found *m/z* 260.10818, [M + H]^+^. C_15_H_15_FNO_2_ [260.10868].

*7-Methoxy-3-(4-methoxyphenyl)-2H-benzo[b][1,4]oxazine* (**10a**). White solid: 40%, mp: 128–130 °C. IR (neat) 2970, 2921, 2842 cm^−1^. ^1^H NMR (400 MHz, DMSO) δ 7.98–7.90 (m, 2H), 7.25 (d, *J* = 8.5 Hz, 1H), 7.09–7.01 (m, 2H), 6.59 (dd, *J* = 8.5, 2.7 Hz, 1H), 6.53 (d, *J* = 2.7 Hz, 1H), 5.12 (s, 2H), 3.84 (s, 3H), 3.76 (s, 3H). ^13^C NMR (101 MHz, DMSO) δ 161.91, 159.61, 156.10, 147.58, 128.53, 128.21, 128.05, 114.55, 108.28, 101.45, 62.68, 55.92, 55.86. HRMS (+ESI): Found *m*/*z* 270.11239, [M + H]^+^. C_16_H_16_NO_3_ [270.11302].

*3-(4-Fluorophenyl)-7-methoxy-2H-benzo[b][1,4]oxazine* (**10b**). Yellow crystal: 5%, mp: 145–147 °C. IR (neat) 2966, 1587, 1306 cm^−1^. ^1^H NMR (400 MHz, DMSO) δ 8.09–7.97 (m, 2H), 7.38–7.31 (m, 2H), 7.28 (d, *J* = 8.6 Hz, 1H), 6.61 (dd, *J* = 8.6, 2.7 Hz, 1H), 6.54 (d, *J* = 2.6 Hz, 1H), 5.16 (s, 2H), 3.77 (s, 3H). ^13^C NMR (101 MHz, DMSO) δ 165.38, 162.90, 160.08, 155.60, 147.61, 132.11, 132.08, 129.28, 129.20, 128.59, 127.75, 116.27, 116.05, 108.41, 101.47, 62.78, 55.97. HRMS (+ESI): Found *m/z* 258.09252, [M + H]^+^. C_15_H_13_FNO_2_ [258.09303].

**General procedure A for the synthesis of 3,4-diphenyl-1,4-benzoxazines**

A Schlenk flask was charged with caesium carbonate (1.4 eq.), the corresponding 1,4-benzoxazine, and bromophenol (1.6 eq.). The flask was vacuumed and backfilled with nitrogen three times before the reaction mixture was dissolved in toluene and *tert*-butanol and stirred for 10 min at room temperature. Pd_2_(dba)_3_ (0.02 eq.) and XPhos (0.1 eq.) were added to the solution and the mixture was refluxed until the reaction showed signs of completion as indicated by TLC. The mixture was cooled to room temperature and diluted with ethyl acetate. The solution was washed with brine and water before being dried with sodium sulphate and concentrated in vacuo to obtain crude product, which was purified by flash chromatography (hexane/ethyl acetate) to obtain the target 3,4-diphenyl-1,4-benzoxazine.

**General procedure B for the synthesis of 3,4-diphenyl-1,4-benzoxazines**

A Schlenk flask was charged with potassium *tert*-butoxide (1.5 eq.), the corresponding 1,4-benzoxazine, and bromophenol (1.6 eq.). The flask was vacuumed and back filled with nitrogen for three times before the reaction mixture was dissolved in toluene and stirred for 10 min at room temperature. Palladium acetate (0.05 eq.) and tri-*tert*-butylphosphine (0.1 eq.) was added to the solution and the mixture was refluxed until the reaction showed signs of completion as indicated by TLC. The mixture was cooled to room temperature and diluted with ethyl acetate. The solution was washed with brine and water before drying with sodium sulphate and then concentrated in vacuo to obtain crude product, which was purified by flash chromatography (hexane/ethyl acetate) to obtain target 3,4-diphenyl-1,4-benzoxazine.

*3,4-Diphenyl-3,4-dihydro-2H-benzo[b][1,4]oxazine* (**5a**). White solid, yield: 50%, mp: 166–168 °C. IR (neat) 2936, 2167, 1493 cm^−1^. ^1^H NMR (400 MHz, DMSO-*d*_6_) δ 7.37–7.26 (m, 6H), 7.27–7.16 (m, 3H), 7.12–7.03 (m, 1H), 6.87 (dd, *J* = 8.1, 1.6 Hz, 1H), 6.83–6.72 (m, 2H), 6.69 (td, *J* = 7.5, 1.6 Hz, 1H), 5.05 (t, *J* = 3.1 Hz, 1H), 4.48 (dd, *J* = 11.0, 3.3 Hz, 1H), 4.30 (dd, *J* = 11.0, 2.8 Hz, 1H). ^13^C NMR (101 MHz, DMSO-*d*_6_) δ 146.25, 144.65, 140.33, 132.83, 129.93, 128.87, 127.67, 127.40, 124.50, 124.48, 121.74, 119.73, 117.22, 116.19, 68.86, 60.66. HRMS (+ESI): Found *m/z* 288.13830, [M + H]+. C_20_H_18_NO [288.13884].

*4-(4-Nitrophenyl)-3-phenyl-3,4-dihydro-2H-benzo[b][1,4]oxazine* (**5b**). Orange solid, yield: 43%, mp: 167–169 °C. IR (neat) 2928, 1583, 1490 cm^−1^. ^1^H NMR (400 MHz, DMSO-*d*_6_) δ 8.23–8.14 (m, 2H), 7.45–7.36 (m, 1H), 7.40–7.30 (m, 6H), 7.27 (ddd, *J* = 9.7, 5.1, 2.3 Hz, 1H), 6.96–6.86 (m, 2H), 6.87–6.79 (m, 1H), 5.37 (d, *J* = 2.4 Hz, 1H), 4.78 (dd, *J* = 11.4, 2.0 Hz, 1H), 4.39 (dd, *J* = 11.4, 2.8 Hz, 1H). ^13^C NMR (101 MHz, DMSO-*d*_6_) δ 152.11, 146.10, 141.02, 138.60, 129.16, 128.01, 127.88, 126.95, 126.08, 123.47, 121.63, 119.66, 119.57, 117.87, 68.32, 59.16. HRMS (+ESI): Found *m*/*z* 333.12349, [M + H]^+^. C_20_H_17_N_2_O_3_ [333.12392].

*3-(4-Methoxyphenyl)-4-phenyl-3,4-dihydro-2H-benzo[b][1,4]oxazine* (**5c**). Pale yellow crystal, yield: 47%, mp: 152–154 °C. IR (neat) 2906, 1489, 1248 cm^−1^. ^1^H NMR (400 MHz, DMSO-*d*_6_) δ 7.38–7.27 (m, 2H), 7.21 (ddd, *J* = 15.6, 7.6, 1.7 Hz, 4H), 7.12–7.03 (m, 1H), 6.89–6.64 (m, 6H), 4.97 (t, *J* = 3.3 Hz, 1H), 4.42 (dd, *J* = 10.9, 3.8 Hz, 1H), 4.26 (dd, *J* = 10.9, 2.8 Hz, 1H), 3.70 (s, 3H). ^13^C NMR (101 MHz, DMSO-*d*_6_) δ 158.86, 146.20, 144.63, 133.08, 131.96, 129.89, 128.63, 124.72, 124.53, 121.66, 119.65, 117.15, 116.15, 114.27, 69.04, 60.01, 55.46. HRMS (+ESI): Found *m*/*z* 318.14909, [M + H]^+^. C_21_H_20_NO_2_ [318.14940].

*3-(4-Methoxyphenyl)-4-(4-nitrophenyl)-3,4-dihydro-2H-benzo[b][1,4]oxazine* (**5d**). Orange solid, yield: 50%, mp: 107–109 °C. IR (neat) 2833, 2161, 2044 cm^−1^. ^1^H NMR (400 MHz, DMSO-*d*_6_) δ 8.23–8.14 (m, 2H), 7.43–7.31 (m, 3H), 7.30–7.23 (m, 2H), 6.92–6.81 (m, 5H), 5.28 (d, *J* = 2.6 Hz, 1H), 4.72 (dd, *J* = 11.4, 2.1 Hz, 1H), 4.36 (dd, *J* = 11.4, 2.8 Hz, 1H), 3.71 (s, 3H). ^13^C NMR (101 MHz, DMSO-*d*_6_) δ 158.54, 151.62, 145.63, 140.48, 129.75, 127.68, 127.54, 125.59, 122.96, 121.08, 119.16, 119.06, 117.36, 114.10, 67.90, 58.15, 55.05. HRMS (+ESI): Found *m*/*z* 363.13424, [M + H]^+^. C_21_H_19_N_2_O_4_ [363.13448].

*4-(4-Fluorophenyl)-3-(4-methoxyphenyl)-3,4-dihydro-2H-benzo[b][1,4]oxazine* (**5e**). Pale yellow solid, yield: 23%, mp: 101–103 °C. IR (neat) 2928, 1495, 1213 cm^−1^. ^1^H NMR (400 MHz, DMSO-*d*_6_) δ 7.25–7.11 (m, 9H), 6.88–6.78 (m, 3H), 6.78–6.63 (m, 2H), 6.61 (dd, *J* = 7.9, 1.7 Hz, 1H), 4.91 (dd, *J* = 4.5, 2.9 Hz, 1H), 4.36 (dd, *J* = 10.9, 4.6 Hz, 1H), 4.27 (dd, *J* = 10.9, 2.9 Hz, 1H), 3.70 (s, 3H). ^13^C NMR (101 MHz, DMSO-*d*_6_) δ 158.95, 144.43, 142.26, 142.23, 134.20, 131.59, 131.24, 131.16, 128.88, 128.11, 128.03, 121.76, 119.38, 117.06, 116.74, 116.52, 115.99, 115.78, 115.54, 114.27, 69.33, 60.26, 55.45. HRMS (+ESI): Found *m*/*z* 336.13957, [M + H]^+^. C_21_H_19_FNO_2_ [336.13998].

*7-Methoxy-3-(4-methoxyphenyl)-4-phenyl-3,4-dihydro-2H-benzo[b][1,4]oxazine* (**11a**). Orange oil, yield: 38%. IR (neat) 2935, 2836, 1502 cm^−1^. ^1^H NMR (400 MHz, DMSO-*d*_6_) δ 7.86–7.76 (m, 1H), 7.52–7.42 (m, 1H), 7.31–7.24 (m, 3H), 7.17–7.09 (m, 2H), 6.99 (tt, *J* = 7.2, 1.2 Hz, 1H), 6.89–6.81 (m, 2H), 6.45–6.37 (m, 2H), 4.93 (t, 1H), 4.50 (dd, *J* = 11.0, 3.3 Hz, 1H), 4.21 (dd, *J* = 11.0, 2.8 Hz, 1H), 3.70 (s, 3H), 3.65 (s, 3H). ^13^C NMR (101 MHz, DMSO-*d*_6_) δ 158.80, 153.82, 147.64, 145.91, 131.76, 129.81, 128.54, 125.26, 123.23, 122.84, 118.82, 114.26, 107.71, 102.75, 68.44, 59.80, 55.67, 55.47. HRMS (+ESI): Found *m*/*z* 348.15951, [M + H]^+^. C_22_H_22_NO_3_ [348.15997].

*7-Methoxy-3,4-bis(4-methoxyphenyl)-3,4-dihydro-2H-benzo[b][1,4]oxazine* (**11b**). Red oil, yield: 82%. IR (neat) 2931, 1596, 1501 cm^−1^. ^1^H NMR (400 MHz, DMSO-*d*_6_) δ 7.27–7.19 (m, 2H), 7.11–7.03 (m, 2H), 6.90–6.79 (m, 4H), 6.48 (d, *J* = 8.9 Hz, 1H), 6.42 (d, *J* = 2.8 Hz, 1H), 6.35 (dd, *J* = 8.9, 2.8 Hz, 1H), 4.79 (dd, *J* = 5.0, 2.8 Hz, 1H), 4.34 (dd, *J* = 10.9, 5.0 Hz, 1H), 4.21 (dd, *J* = 10.9, 2.9 Hz, 1H), 3.70 (d, *J* = 1.2 Hz, 6H), 3.64 (s, 3H). ^13^C NMR (101 MHz, DMSO-*d*_6_) δ 158.86, 156.48, 153.01, 145.24, 140.07, 131.73, 128.97, 128.48, 127.17, 117.18, 115.06, 114.17, 107.52, 102.82, 69.37, 60.42, 55.71, 55.61, 55.44. HRMS (+ESI): Found *m*/*z* 378.16984, [M + H]^+^. C_23_H_24_NO_4_ [378.17053].

*7-Methoxy-3-(4-methoxyphenyl)-4-(4-nitrophenyl)-3,4-dihydro-2H-benzo[b][1,4]oxazine* (**11c**). Red oil, yield: 24%. IR (neat) 2923, 1598, 1495 cm^−1^. ^1^H NMR (400 MHz, DMSO-*d*_6_) δ 8.20–8.11 (m, 2H), 7.31–7.24 (m, 5H), 6.94–6.85 (m, 2H), 6.52 (dd, *J* = 9.0, 2.8 Hz, 1H), 6.42 (d, *J* = 2.8 Hz, 1H), 5.31 (s, 1H), 4.77 (dd, *J* = 11.5, 1.9 Hz, 1H), 4.33 (dd, *J* = 11.4, 2.8 Hz, 1H), 3.70 (d, *J* = 11.3 Hz, 6H). ^13^C NMR (101 MHz, DMSO-*d*_6_) δ 158.99, 156.12, 152.65, 147.36, 140.08, 129.91, 128.20, 126.20, 121.76, 120.43, 118.10, 114.54, 108.03, 102.68, 68.25, 58.23, 55.73, 55.53. HRMS (+ESI): Found *m*/*z* 393.14461, [M + H]^+^. C_22_H_21_N_2_O_5_ [393.14505].

*3-(2,4-Dimethylphenyl)-7-methoxy-4-(4-methoxyphenyl)-3,4-dihydro-2H-benzo[b][1,4]oxazine* (**11d**). Light brown gum, yield: 38%. IR (neat) 2921, 1586, 1501 cm^−1^. ^1^H NMR (400 MHz, DMSO-*d*_6_) δ 7.18–7.13 (m, 1H), 7.13–7.06 (m, 2H), 6.89 (d, *J* = 6.7 Hz, 2H), 6.87–6.81 (m, 2H), 6.46 (dd, *J* = 7.9, 2.8 Hz, 1H), 6.38 (d, *J* = 8.8 Hz, 1H), 6.33 (dd, *J* = 8.9, 2.7 Hz, 1H), 5.00 (dd, *J* = 5.9, 3.0 Hz, 1H), 4.25 (dd, *J* = 11.0, 3.1 Hz, 1H), 4.17 (dd, *J* = 11.0, 5.9 Hz, 1H), 3.69 (s, 3H), 3.65 (s, 3H), 2.24 (s, 3H), 2.18 (s, 3H). ^13^C NMR (101 MHz, DMSO-*d*_6_) δ 156.90, 152.66, 144.90, 139.31, 136.50, 135.29, 134.41, 131.49, 130.19, 128.28, 128.09, 126.96, 115.96, 115.01, 107.40, 102.94, 69.02, 57.27, 55.75, 55.56, 20.97, 19.25. HRMS (+ESI): Found *m*/*z* 376.19085, [M + H]^+^. C_24_H_26_NO_3_ [376.19127].

*3-(2,4-Dimethylphenyl)-7-methoxy-4-(4-nitrophenyl)-3,4-dihydro-2H-benzo[b][1,4]oxazine* (**11e**). Red solid, yield: 78%, mp: 121–123 °C. IR (neat) 2929, 1585, 1493 cm^−1^. ^1^H NMR (400 MHz, Chloroform-*d*) δ 8.15–8.06 (m, 2H), 7.30 (d, *J* = 8.6 Hz, 1H), 7.19–7.12 (m, 2H), 7.09–7.06 (m, 1H), 7.04 (d, *J* = 7.9 Hz, 1H), 6.94–6.87 (m, 1H), 6.61–6.53 (m, 2H), 5.11 (t, *J* = 3.6 Hz, 1H), 4.47–4.39 (m, 1H), 4.35 (dd, *J* = 11.0, 3.5 Hz, 1H), 3.81 (s, 3H), 2.42 (s, 3H), 2.31 (s, 3H). ^13^C NMR (101 MHz, CDCl_3_) δ 155.74, 152.55, 147.71, 141.04, 137.72, 133.65, 133.30, 132.04, 127.42, 126.61, 125.55, 122.89, 119.79, 118.57, 108.20, 103.00, 68.33, 58.86, 55.60, 31.60, 22.67, 20.97, 19.29, 14.14. HRMS (+ESI): Found *m*/*z* 391.16525, [M + H]^+^. C_23_H_23_N_2_O_4_ [391.16578].

*3-(2,4-Dimethylphenyl)-7-methoxy-4-(quinolin-3-yl)-3,4-dihydro-2H-benzo[b][1,4]oxazine* (**15a**). Yellow solid, yield: 55%, mp: 112–114 °C. IR (neat) 2923, 1587, 1505 cm^−1^. ^1^H NMR (400 MHz, DMSO-*d*_6_) δ 8.79 (d, *J* = 2.6 Hz, 1H), 8.00 (d, *J* = 2.6 Hz, 1H), 7.91 (d, *J* = 8.4 Hz, 1H), 7.82 (dd, *J* = 8.3, 1.4 Hz, 1H), 7.63 (ddd, *J* = 8.4, 6.8, 1.5 Hz, 1H), 7.52 (ddd, *J* = 8.2, 6.8, 1.3 Hz, 1H), 7.13 (d, *J* = 7.9 Hz, 1H), 6.95 (d, *J* = 1.8 Hz, 1H), 6.91–6.82 (m, 1H), 6.76 (d, *J* = 8.9 Hz, 1H), 6.53 (d, *J* = 2.9 Hz, 1H), 6.43 (dd, *J* = 8.9, 2.9 Hz, 1H), 5.32 (d, *J* = 3.7 Hz, 1H), 4.41–4.26 (m, 2H), 3.69 (s, 3H), 2.34 (s, 3H), 2.17 (s, 3H). ^13^C NMR (101 MHz, DMSO-*d*_6_) δ 153.93, 149.67, 145.91, 144.82, 140.22, 136.84, 135.37, 134.10, 131.80, 128.95, 128.71, 128.65, 128.36, 127.89, 127.63, 127.40, 127.24, 127.06, 116.96, 107.83, 103.22, 68.51, 57.39, 55.79, 20.94, 19.33. HRMS (+ESI): Found *m*/*z* 397.19073, [M + H]^+^. C_26_H_25_N_2_O_2_ [397.19160].

*3-(4-Fluorophenyl)-7-methoxy-4-(4-methoxyphenyl)-3,4-dihydro-2H-benzo[b][1,4]oxazine* (**11f**). Yellow oil, yield: 83%. IR (neat) 2938, 2835, 1501 cm^−1^. ^1^H NMR (400 MHz, DMSO-*d*_6_) δ 7.41–7.31 (m, 2H), 7.16–7.03 (m, 4H), 6.91–6.82 (m, 2H), 6.53 (d, *J* = 8.9 Hz, 1H), 6.44 (d, *J* = 2.8 Hz, 1H), 6.37 (dd, *J* = 8.9, 2.8 Hz, 1H), 4.88 (t, *J* = 3.7 Hz, 1H), 4.38 (dd, *J* = 10.9, 4.6 Hz, 1H), 4.24 (dd, *J* = 10.9, 2.8 Hz, 1H), 3.70 (s, 3H), 3.65 (s, 3H). ^13^C NMR (101 MHz, DMSO-*d*_6_) δ 163.00, 160.58, 156.54, 153.13, 145.23, 140.07, 136.26, 136.23, 129.80, 129.72, 128.03, 127.03, 117.37, 115.65, 115.44, 115.13, 107.72, 102.86, 68.99, 60.48, 55.70, 55.62. HRMS (+ESI): Found *m*/*z* 388.13199, [M + Na]^+^. C_22_H_20_FNO_3_Na [388.13249].

*3-(4-Fluorophenyl)-7-methoxy-4-(4-nitrophenyl)-3,4-dihydro-2H-benzo[b][1,4]oxazine* (**11g**). Yellow solid, yield: 33%, mp: 139–141 °C. IR (neat) 2912, 1589, 1494 cm^−1^. ^1^H NMR (400 MHz, DMSO-*d*_6_) δ 8.21–8.08 (m, 2H), 7.45–7.36 (m, 2H), 7.35–7.24 (m, 3H), 7.24–7.12 (m, 2H), 6.53 (dd, *J* = 9.0, 2.8 Hz, 1H), 6.43 (d, *J* = 2.8 Hz, 1H), 5.38 (s, 1H), 4.81 (dd, *J* = 11.5, 1.9 Hz, 1H), 4.34 (dd, *J* = 11.5, 2.8 Hz, 1H), 3.69 (s, 3H). ^13^C NMR (101 MHz, DMSO-*d*_6_) δ 163.10, 160.68, 156.18, 152.65, 147.29, 140.27, 134.45, 134.42, 129.15, 129.07, 126.19, 121.93, 120.24, 118.28, 116.03, 115.82, 108.20, 102.70, 68.09, 58.30, 55.73. HRMS (+ESI): Found *m*/*z* 403.10655, [M + Na]^+^. C_21_H_17_FN_2_O_4_Na [403.10700].

*3-(4-Fluorophenyl)-7-methoxy-2H-benzo[b][1,4]oxazine* (**12**). Dark purple solid: 35%, mp: 159–161 °C. IR (neat) 2989, 2935, 2834 cm^−1^. ^1^H NMR (400 MHz, DMSO) δ 7.83–7.72 (m, 2H), 7.34 (d, *J* = 8.6 Hz, 1H), 7.04–6.93 (m, 2H), 6.72 (d, *J* = 8.5 Hz, 2H), 6.69 (s, 2H), 6.65 (d, *J* = 2.4 Hz, 1H), 6.56 (dd, *J* = 8.6, 2.7 Hz, 1H), 6.36 (d, *J* = 2.7 Hz, 1H), 6.32 (dd, *J* = 8.6, 2.4 Hz, 1H), 3.95 (s, 3H), 3.77 (s, 3H), 3.69 (s, 6H). ^13^C NMR (101 MHz, DMSO) δ 162.12, 161.70, 160.02, 158.53, 156.13, 145.90, 129.79, 128.34, 128.12, 128.07, 127.76, 116.04, 114.59, 108.16, 105.44, 102.18, 99.64, 66.66, 56.49, 55.82, 55.80, 55.70. HRMS (+ESI): Found *m*/*z* 406.16517, [M + H]^+^. C_24_H_24_NO_5_ [406.16545].

*3-(4-Fluorophenyl)-7-methoxy-4-(quinolin-3-yl)-3,4-dihydro-2H-benzo[b][1,4]oxazine* (**15b**). Brown gum, yield: 67%. IR (neat) 3078, 3002, 1507 cm^−1^. ^1^H NMR (400 MHz, DMSO-*d*_6_) δ 8.83 (d, *J* = 2.6 Hz, 1H), 7.93 (d, *J* = 8.4 Hz, 1H), 7.90 (d, *J* = 2.7 Hz, 1H), 7.82 (d, *J* = 7.5 Hz, 1H), 7.61 (ddd, *J* = 8.4, 6.9, 1.5 Hz, 1H), 7.55–7.44 (m, 3H), 7.21–7.11 (m, 2H), 7.02–6.95 (m, 1H), 6.51–6.44 (m, 2H), 5.26 (s, 1H), 4.64 (dd, *J* = 11.2, 3.1 Hz, 1H), 4.34 (dd, *J* = 11.2, 2.7 Hz, 1H), 3.69 (s, 3H). ^13^C NMR (101 MHz, DMSO-*d*_6_) δ 163.07, 160.65, 154.77, 148.15, 146.49, 144.34, 141.22, 135.56, 135.53, 129.55, 129.47, 128.94, 128.79, 128.16, 127.70, 127.49, 125.34, 123.92, 119.52, 115.88, 115.67, 108.20, 102.96, 67.97, 59.71, 55.72. HRMS (+ESI): Found *m*/*z* 387.15037, [M + H]^+^. C_24_H_20_FN_2_O_2_ [387.15088].

**General procedure for the synthesis of 3-phenyl-4-aniline-1,4-benzoxazine**

Palladium on carbon (10% wt, 0.1 eq.) was added to a solution of the corresponding 3-phenyl-4-aniline-1,4-benzoxazine in methanol at room temperature and stirred for 16 h. The reaction mixture was filtered through celite and the filtrate removed in vacuo to obtain crude product, which was purified by flash chromatography (hexane/ethyl acetate) to obtain target 3-phenyl-4-aniline-1,4-benzoxazine.

*4-(3-Phenyl-2,3-dihydro-4H-benzo[b][1,4]oxazin-4-yl)aniline* (**13a**). Brown solid, yield: 85%, mp: 169–171 °C. IR (neat) 3454, 3374, 2920 cm^−1^. ^1^H NMR (400 MHz, DMSO-*d*_6_) δ 7.33–7.15 (m, 5H), 6.89–6.81 (m, 2H), 6.76 (dd, *J* = 7.8, 1.5 Hz, 1H), 6.68 (ddd, *J* = 8.0, 7.3, 1.6 Hz, 1H), 6.57 (td, *J* = 7.6, 1.6 Hz, 1H), 6.53–6.41 (m, 3H), 5.00 (s, 2H), 4.83 (t, *J* = 3.8 Hz, 1H), 4.32–4.21 (m, 2H). ^13^C NMR (101 MHz, DMSO-*d*_6_) δ 146.59, 143.41, 140.24, 135.86, 133.58, 128.20, 127.96, 127.38, 127.17, 121.34, 117.26, 116.16, 114.52, 114.15, 69.15, 61.04. HRMS (+ESI): Found *m*/*z* 303.14921, [M + H]^+^. C_20_H_19_N_2_O [303.14974].

*4-(3-(4-Methoxyphenyl)-2,3-dihydro-4H-benzo[b][1,4]oxazin-4-yl)aniline* (**13b**). Yellow solid, yield: 78%, mp: 170–172 °C. IR (neat) 3456, 3371, 2932 cm^−1^. ^1^H NMR (400 MHz, DMSO-*d*_6_) δ 7.23–7.15 (m, 2H), 6.87–6.79 (m, 4H), 6.76 (dd, *J* = 7.8, 1.6 Hz, 1H), 6.71–6.62 (m, 1H), 6.56 (td, *J* = 7.5, 1.6 Hz, 1H), 6.52–6.44 (m, 2H), 6.41 (dd, *J* = 8.0, 1.5 Hz, 1H), 5.00 (s, 2H), 4.75 (t, *J* = 4.0 Hz, 1H), 4.22 (d, *J* = 4.0 Hz, 2H), 3.70 (s, 3H). ^13^C NMR (101 MHz, DMSO-*d*_6_) δ 158.85, 147.04, 143.91, 136.60, 133.99, 132.29, 129.07, 128.59, 121.72, 117.74, 116.56, 114.98, 114.73, 114.07, 69.87, 60.74, 55.42. HRMS (+ESI): Found *m*/*z* 333.15971, [M + H]^+^. C_21_H_21_N_2_O_2_ [333.16030].

*4-(7-Methoxy-3-(4-methoxyphenyl)-2,3-dihydro-4H-benzo[b][1,4]oxazin-4-yl)aniline* (**13c**). Dark-brown solid, yield: 100%, mp: 142–144 °C. IR (neat) 3448, 2929, 2837 cm^−1^. ^1^H NMR (400 MHz, DMSO-*d*_6_) δ 7.28–7.13 (m, 2H), 6.88–6.73 (m, 4H), 6.50–6.42 (m, 2H), 6.42–6.25 (m, 3H), 4.93 (s, 2H), 4.68 (dd, *J* = 5.6, 3.0 Hz, 1H), 4.32–4.13 (m, 2H), 3.70 (s, 3H), 3.63 (s, 3H). ^13^C NMR (101 MHz, DMSO-*d*_6_) δ 158.82, 152.40, 146.54, 144.82, 135.40, 132.00, 130.17, 129.14, 127.96, 116.46, 114.98, 114.05, 107.33, 102.75, 69.89, 60.59, 55.72, 55.41. HRMS (+ESI): Found *m*/*z* 363.17036, [M + H]^+^. C_22_H_23_N_2_O_3_ [363.17087].

*4-(3-(2,4-Dimethylphenyl)-7-methoxy-2,3-dihydro-4H-benzo[b][1,4]oxazin-4-yl)aniline* (**13d**). Brown solid, yield: 75%, mp: 119–121 °C. IR (neat) 3466, 3377, 2883 cm^−1^. ^1^H NMR (400 MHz, DMSO-*d*_6_) δ 7.17 (d, *J* = 7.8 Hz, 1H), 6.93–6.85 (m, 2H), 6.86–6.78 (m, 2H), 6.48–6.37 (m, 3H), 6.30 (d, *J* = 1.6 Hz, 2H), 4.94 (s, 2H), 4.91 (dd, *J* = 6.4, 3.0 Hz, 1H), 4.22 (dd, *J* = 10.9, 3.0 Hz, 1H), 4.12 (dd, *J* = 10.9, 6.4 Hz, 1H), 3.64 (s, 3H), 2.22 (s, 3H), 2.18 (s, 3H). ^13^C NMR (101 MHz, DMSO-*d*_6_) δ 152.21, 146.82, 144.62, 136.36, 135.33, 134.85, 134.56, 131.40, 131.35, 128.52, 128.30, 126.89, 115.76, 114.95, 107.23, 102.83, 69.29, 57.17, 55.75, 20.99, 19.26. HRMS (+ESI): Found *m*/*z* 361.19108, [M + H]^+^. C_23_H_25_N_2_O_2_ [361.19160].

*4-(3-(4-Fluorophenyl)-7-methoxy-2,3-dihydro-4H-benzo[b][1,4]oxazin-4-yl)aniline* (**13e**).

Dark-brown solid, yield: 43%, mp: 128–130 °C. IR (neat) 3455, 3364, 2836 cm^−1^. ^1^H NMR (400 MHz, Chloroform-*d*) δ 7.32–7.23 (m, 2H), 7.04–6.93 (m, 2H), 6.97–6.89 (m, 2H), 6.65–6.57 (m, 3H), 6.52 (d, *J* = 2.8 Hz, 1H), 6.39 (dd, *J* = 8.9, 2.8 Hz, 1H), 4.68 (dd, *J* = 5.1, 3.1 Hz, 1H), 4.37 (dd, *J* = 10.8, 5.1 Hz, 1H), 4.31 (dd, *J* = 10.8, 3.1 Hz, 1H), 3.75 (s, 3H), 3.64 (s, 2H). ^13^C NMR (101 MHz, CDCl_3_) δ 163.35, 160.91, 152.98, 144.97, 143.41, 138.35, 135.41, 135.38, 129.16, 129.08, 128.72, 127.54, 117.25, 116.08, 115.46, 115.24, 107.56, 102.61, 69.38, 61.52, 55.67. HRMS (+ESI): Found *m*/*z* 351.15036, [M + H]^+^. C_21_H_20_FN_2_O_2_ [351.15088].

**General procedure for demethylation using BBr_3_**

BBr_3_ (1M) in dichloromethane solution (2 eq. per methoxy group) was added to a solution of the corresponding methylated 3,4-diphenyl-1,4-benzoxazine in dichloromethane at 0 °C and stirred for 12 h. The reaction mixture was quenched with saturated sodium bicarbonate solution and diluted with ethyl acetate. The solution was washed with brine and water before drying with sodium sulphate and concentrated in vacuo to obtain crude product, which was purified by flash chromatography (dichloromethane/methanol) to obtain target demethylated 3,4-diphenyl-1,4-benzoxazine.

*4-(4-Phenyl-3,4-dihydro-2H-benzo[b][1,4]oxazin-3-yl)phenol* (**14a**). White solid, yield: 67%, mp: 150–152 °C. IR (neat) 3516, 1586, 1490 cm^−1^. ^1^H NMR (400 MHz, DMSO-*d*_6_) δ 9.32 (s, 1H), 7.37–7.27 (m, 2H), 7.22–7.14 (m, 2H), 7.14–7.03 (m, 3H), 6.83–6.62 (m, 6H), 4.90 (t, *J* = 3.3 Hz, 1H), 4.38 (dd, *J* = 10.9, 3.9 Hz, 1H), 4.25 (dd, *J* = 10.9, 2.8 Hz, 1H). ^13^C NMR (101 MHz, DMSO-*d*_6_) δ 156.97, 146.22, 144.62, 133.29, 130.12, 129.85, 128.62, 124.92, 124.55, 121.61, 119.53, 117.10, 116.08, 115.59, 69.13, 60.10. HRMS (+ESI): Found *m*/*z* 304.13317, [M + H]^+^. C_20_H_18_NO_2_ [304.13375].

*4-(4-(4-Aminophenyl)-3,4-dihydro-2H-benzo[b][1,4]oxazin-3-yl)phenol* (**14b**). Brown solid, yield: 62%, mp: 113–115 °C. IR (neat) 3438, 3352, 2884 cm^−1^. ^1^H NMR (400 MHz, DMSO-*d*_6_) δ 9.28 (s, 1H), 7.10–7.02 (m, 2H), 6.85–6.77 (m, 2H), 6.75 (dd, *J* = 7.8, 1.6 Hz, 1H), 6.69–6.60 (m, 3H), 6.55 (td, *J* = 7.5, 1.6 Hz, 1H), 6.52–6.44 (m, 2H), 6.39 (dd, *J* = 8.1, 1.6 Hz, 1H), 4.99 (s, 2H), 4.68 (t, *J* = 4.1 Hz, 1H), 4.22–4.18 (m, 2H). ^13^C NMR (101 MHz, DMSO-*d*_6_) δ 156.95, 146.98, 143.91, 136.72, 134.06, 130.49, 129.04, 128.68, 121.67, 117.65, 116.52, 115.42, 114.97, 114.70, 69.94, 60.84. HRMS (+ESI): Found *m*/*z* 319.14402, [M + H]^+^. C_20_H_19_N_2_O_2_ [319.14465].

*3-(4-Hydroxyphenyl)-4-phenyl-3,4-dihydro-2H-benzo[b][1,4]oxazin-7-ol* (**14c**). Brown solid, yield: 40%, mp: 129–131 °C. IR (neat) 3189, 3927, 2865 cm^−1^. ^1^H NMR (400 MHz, DMSO-*d*_6_) δ 9.30 (s, 1H), 8.92 (s, 1H), 7.31–7.21 (m, 2H), 7.17–7.06 (m, 4H), 6.96 (tt, *J* = 7.1, 1.1 Hz, 1H), 6.73 (d, *J* = 8.5 Hz, 1H), 6.71–6.62 (m, 2H), 6.27–6.19 (m, 2H), 4.84 (t, *J* = 3.0 Hz, 1H), 4.43 (dd, *J* = 11.0, 3.4 Hz, 1H), 4.16 (dd, *J* = 11.0, 2.8 Hz, 1H). ^13^C NMR (101 MHz, DMSO-*d*_6_) δ 156.85, 151.84, 148.11, 146.04, 130.05, 129.69, 128.52, 123.77, 122.78, 122.58, 119.28, 115.54, 108.79, 103.97, 68.27, 59.93. HRMS (+ESI): Found *m*/*z* 342.11001, [M + Na]^+^. C_20_H_17_NO_3_Na [342.11061].

*4,4′-(7-Hydroxy-2,3-dihydro-4H-benzo[b][1,4]oxazine-3,4-diyl)diphenol* (**14d**). Black solid, yield: 70%, mp: 91–93 °C. IR (neat) 3023, 2814, 2692 cm^−1^. ^1^H NMR (400 MHz, DMSO-*d*_6_) δ 9.28 (s, 1H), 9.22 (s, 1H), 8.74 (s, 1H), 7.12–7.04 (m, 2H), 6.95–6.87 (m, 2H), 6.69–6.59 (m, 4H), 6.30 (d, *J* = 8.7 Hz, 1H), 6.22 (d, *J* = 2.6 Hz, 1H), 6.15 (dd, *J* = 8.7, 2.7 Hz, 1H), 4.62 (dd, *J* = 5.5, 2.9 Hz, 1H), 4.24 (dd, *J* = 10.8, 5.5 Hz, 1H), 4.14 (dd, *J* = 10.8, 2.9 Hz, 1H). ^13^C NMR (101 MHz, DMSO-*d*_6_) δ 156.90, 154.62, 150.61, 145.15, 138.92, 130.08, 129.05, 127.88, 127.65, 117.31, 116.23, 115.42, 108.57, 103.88, 69.52, 60.68. HRMS (+ESI): Found *m*/*z* 358.10491, [M + Na]^+^. C_20_H_17_NO_4_Na [358.10553].

*4-(4-Aminophenyl)-3-(4-hydroxyphenyl)-3,4-dihydro-2H-benzo[b][1,4]oxazin-7-ol* (**14e**). Black gum, yield: 75%. IR (neat) 2929, 1596, 1502 cm^−1^. ^1^H NMR (400 MHz, DMSO-*d*_6_) δ 9.26 (s, 1H), 8.68 (s, 1H), 7.13–6.99 (m, 2H), 6.82–6.71 (m, 2H), 6.68–6.57 (m, 2H), 6.50–6.39 (m, 2H), 6.28–6.18 (m, 2H), 6.13 (dd, *J* = 8.7, 2.7 Hz, 1H), 4.89 (s, 2H), 4.56 (dd, *J* = 5.8, 3.1 Hz, 1H), 4.19 (dd, *J* = 10.8, 5.8 Hz, 1H), 4.13 (dd, *J* = 10.8, 3.1 Hz, 1H). ^13^C NMR (101 MHz, DMSO-*d*_6_) δ 156.87, 150.21, 146.30, 144.93, 135.98, 130.24, 129.15, 128.89, 127.94, 116.93, 115.36, 114.92, 108.46, 103.80, 69.87, 60.76. HRMS (+ESI): Found *m*/*z* 335.13894, [M + H]^+^. C_20_H_19_N_2_O_3_ [335.13957].

*4-(4-Aminophenyl)-3-(2,4-dimethylphenyl)-3,4-dihydro-2H-benzo[b][1,4]oxazin-7-ol* (**14f**). Pale-brown Solid, yield: 89%, mp: 185–187 °C. IR (neat) 3373, 3304, 2920 cm^−1^. ^1^H NMR (400 MHz, DMSO-*d*_6_) δ 8.67 (s, 1H), 7.18 (d, *J* = 7.8 Hz, 1H), 6.94–6.84 (m, 2H), 6.84–6.74 (m, 2H), 6.47–6.37 (m, 2H), 6.24 (d, *J* = 2.6 Hz, 1H), 6.20 (d, *J* = 8.7 Hz, 1H), 6.13 (dd, *J* = 8.7, 2.6 Hz, 1H), 4.91 (s, 2H), 4.86 (dd, *J* = 6.7, 3.0 Hz, 1H), 4.17 (dd, *J* = 10.9, 3.0 Hz, 1H), 4.08 (dd, *J* = 10.9, 6.7 Hz, 1H), 2.22 (s, 3H), 2.18 (s, 3H). ^13^C NMR (101 MHz, DMSO-*d*_6_) δ 150.05, 146.65, 144.70, 136.28, 135.35, 135.29, 134.57, 131.30, 130.05, 128.47, 128.39, 126.87, 116.20, 114.90, 108.45, 103.85, 69.30, 57.18, 20.99, 19.28. HRMS (+ESI): Found *m*/*z* 347.17541, [M + H]^+^. C_22_H_23_N_2_O_2_ [347.17595].

*3-(4-Fluorophenyl)-4-(4-hydroxyphenyl)-3,4-dihydro-2H-benzo[b][1,4]oxazin-7-ol* (**14g**). Brown gum, yield: 95%. IR (neat) 3401, 1698, 1502 cm^−1^. ^1^H NMR (400 MHz, DMSO-*d*_6_) δ 9.25 (s, 1H), 8.79 (s, 1H), 7.40–7.30 (m, 2H), 7.14–7.04 (m, 2H), 6.97–6.89 (m, 2H), 6.71–6.62 (m, 2H), 6.38 (d, *J* = 8.7 Hz, 1H), 6.24 (d, *J* = 2.6 Hz, 1H), 6.19 (dd, *J* = 8.7, 2.7 Hz, 1H), 4.78 (dd, *J* = 5.0, 2.8 Hz, 1H), 4.31 (dd, *J* = 10.9, 5.0 Hz, 1H), 4.18 (dd, *J* = 10.9, 2.8 Hz, 1H). ^13^C NMR (101 MHz, DMSO-*d*_6_) δ 162.95, 160.53, 154.68, 150.83, 145.16, 138.95, 136.41, 136.38, 129.89, 129.81, 127.32, 127.17, 117.59, 116.34, 115.55, 115.34, 108.84, 103.95, 68.98, 60.63. HRMS (+ESI): Found *m*/*z* 360.10063, [M + Na]^+^. C_20_H_16_FNO_3_Na [360.10119].

*3-(4-Fluorophenyl)-4-(quinolin-3-yl)-3,4-dihydro-2H-benzo[b][1,4]oxazin-7-ol* (**15c**). Brown gum, yield: 87%. IR (neat) 2923, 2798, 1598 cm^−1^. ^1^H NMR (400 MHz, DMSO-*d*_6_) δ 9.12 (s, 1H), 8.84 (d, *J* = 2.7 Hz, 1H), 7.99–7.85 (m, 2H), 7.88–7.78 (m, 1H), 7.60 (ddd, *J* = 8.4, 6.9, 1.5 Hz, 1H), 7.52 (ddd, *J* = 8.2, 6.9, 1.3 Hz, 1H), 7.51–7.42 (m, 2H), 7.21–7.07 (m, 2H), 6.90 (d, *J* = 8.7 Hz, 1H), 6.36–6.26 (m, 2H), 5.24 (d, *J* = 3.1 Hz, 1H), 4.61 (dd, *J* = 11.2, 3.0 Hz, 1H), 4.30 (dd, *J* = 11.2, 2.7 Hz, 1H). ^13^C NMR (101 MHz, DMSO-*d*_6_) δ 163.04, 160.63, 153.02, 147.79, 146.65, 143.82, 141.66, 135.59, 135.56, 129.56, 129.48, 128.86, 128.66, 128.07, 127.65, 127.54, 124.89, 122.10, 120.05, 115.83, 115.62, 109.37, 104.22, 67.68, 59.68. HRMS (+ESI): Found *m*/*z* 373.13476, [M + H]^+^. C_23_H_18_FN_2_O_2_ [373.13523].

## 4. Conclusions

In summary, we developed a facile and efficient approach to synthesise derivatives of a novel 4-aryl-3,4-dihydro-2*H*-1,4-benzoxazine scaffold with anticancer activity. A total of 29 novel analogues were designed and synthesised for preliminary biological screening. Ten of the synthesised molecules showed positive results against the proliferation of MIA PaCa-2, MDA-MB-231 and PC-3 cancer cells. Compound **14f** was identified as a promising lead molecule for further in-depth studies, and subsequent dose-response experiments revealed IC_50_ values of 7.84–16.2 µM against a panel of five cancer cell lines. Preliminary SAR studies suggested that hydroxyl groups on rings A and B and a 4-amino group on ring C were important for anticancer activity. However, it should be noted that the 1,4-benzoxazines were synthesised as racemic mixtures in this study. Future investigations could be conducted to understand the effect of chirality on antiproliferative properties. Additionally, the precise mechanism of the anticancer activity remains to be explored. Investigating the effects of these compounds on particular signalling and metabolic systems, such as estrogen receptor, could provide an explanation for the established SAR and generate suggestions for the further structural improvement of these molecules.

## Figures and Tables

**Table 1:** Cell growth inhibitory effect of the synthesised compounds and their cell growth inhibitory values in different cancer cell lines. Staurosporine (STS) was used as positive control.

Compounds	Cell Growth Inhibition (%)		
	MIA PaCa-2	MDA-MB-231	PC-3
**5a**	21 ± 4.5	9.1 ± 3.8	13 ± 0.5
**5b**	30 ± 8.6	15 ± 0.9	0.8 ± 2.2
**5c**	26 ± 3.9	44 ± 2.1	2.2 ± 0.9
**5d**	21 ± 7.3	23 ± 1.3	0.2 ± 7.7
**5e**	59 ± 6.3	53 ± 3.7	1.0 ± 2.8
**10a**	−4.2 ± 4.4	−7.2 ± 2.4	−2.6 ± 0.6
**10b**	17 ± 2.7	3.4 ± 1.9	7.2 ± 0.4
**11a**	91 ± 1.1	42 ± 11	11 ± 2.5
**11b**	44 ± 4.0	24 ± 0.3	−1.1 ± 1.6
**11d**	48 ± 4.5	22 ± 5.6	−0.6 ± 3.8
**11e**	19 ± 3.2	14 ± 2.7	3.6 ± 2.2
**11f**	33 ± 5.5	19 ± 9.8	−1.3 ± 2.0
**11g**	12 ± 1.8	28 ± 3.9	−4.9 ± 1.1
**12**	37 ± 6.4	24 ± 1.0	−5.6 ± 0.3
**13a**	37 ± 3.8	3.2 ± 3.0	−5.2 ± 0.6
**13b**	25 ± 12	−1.8 ± 2.9	−12 ± 6.9
**13c**	59 ± 4.6	0.5 ± 0.1	25 ± 0.0
**13d**	79 ± 1.5	20 ± 6.7	50 ± 6.2
**13e**	52 ± 0.7	17 ± 1.5	−18 ± 3.2
**14a**	88 ± 0.8	62 ± 8.8	25 ± 6.5
**14b**	57 ± 11	11 ± 0.9	11 ± 3.3
**14c**	75 ± 15	52 ± 1.6	12 ± 1.8
**14d**	94 ± 6.5	45 ± 0.9	1.6 ± 5.0
**14e**	5.6 ± 3.9	−4.8 ± 1.2	−5.3 ± 1.4
**14f**	99 ± 0.5	78 ± 0.4	98 ± 0.8
**14g**	63 ± 7.0	14 ± 4.1	−1.6 ± 5.9
**15a**	72 ± 0.7	36 ± 0.9	12 ± 8.6
**15b**	46 ± 3.7	28 ± 3.9	1.6 ± 0.5
	**IC_50_ (µM)**		
**STS**	0.0221	0.0029	0.0591

**Table 2:** In silico predicted parameters of the compounds.

Compounds	Lipinski Rule				
	MW	Accept HB	Donor HB	Log P_o/w_	Violation
**5a**	287.36	1	0	4.04	0
**5b**	332.35	3	0	3.6	0
**5c**	317.38	2	0	4.03	0
**5d**	362.38	4	0	3.43	0
**5e**	335.37	3	0	4.34	0
**10a**	269.3	4	0	2.96	0
**10b**	257.26	4	0	3.28	0
**11a**	347.41	3	0	4.03	0
**11b**	377.43	4	0	4	0
**11d**	375.46	3	0	4.64	0
**11e**	390.43	4	0	4.08	0
**11f**	365.4	4	0	4.32	0
**11g**	380.37	5	0	3.73	0
**12**	405.44	5	0	4.14	0
**13a**	302.37	1	1	3.48	0
**13b**	332.40	2	1	3.47	0
**13c**	362.42	3	1	3.47	0
**13d**	360.45	2	1	4.13	0
**13e**	350.39	3	1	3.79	0
**14a**	303.35	2	1	3.64	0
**14b**	318.37	2	2	3.08	0
**14c**	319.35	3	2	3.22	0
**14d**	335.35	4	3	2.81	0
**14e**	334.37	3	3	2.66	0
**14f**	346.42	2	2	3.71	0
**14g**	337.34	4	2	3.52	0
**15a**	396.48	3	0	4.94	0
**15b**	386.42	4	0	4.61	0

**Description: Lipinski rule**—Number of violations of Lipinski’s rule of five. The rules are as follows: MW < 500, accept HB ≤ 10, donor HB < 5, Log P_o/w_ < 5 and maximum 4 violations.

## Data Availability

The data presented in this study are available in article and supplementary material.

## Supplementary Materials

The following supporting information can be downloaded at: https://www.mdpi.com/article/10.3390/molecules29010166/s1, ^1^H and ^13^C NMR spectra of the synthesised compounds, and information about the cell lines used.
