# Supplementary material for: Rational Design, Synthesis, and Anti-Proliferative Evaluation of Novel 4-Aryl-3,4-Dihydro-2H-1,4-Benzoxazines
Source: Molecules. 2023 Dec 27;29(1):166. doi: 10.3390/molecules29010166 (PMC10780242; doi:10.3390/molecules29010166)
Supplement: Supplementary file 1 [file molecules-29-00166-s001.zip › molecules-2719772-supplementary.pdf]

*2-(2-nitrophenoxy)-1-phenylethan-1-one (**3a**)*

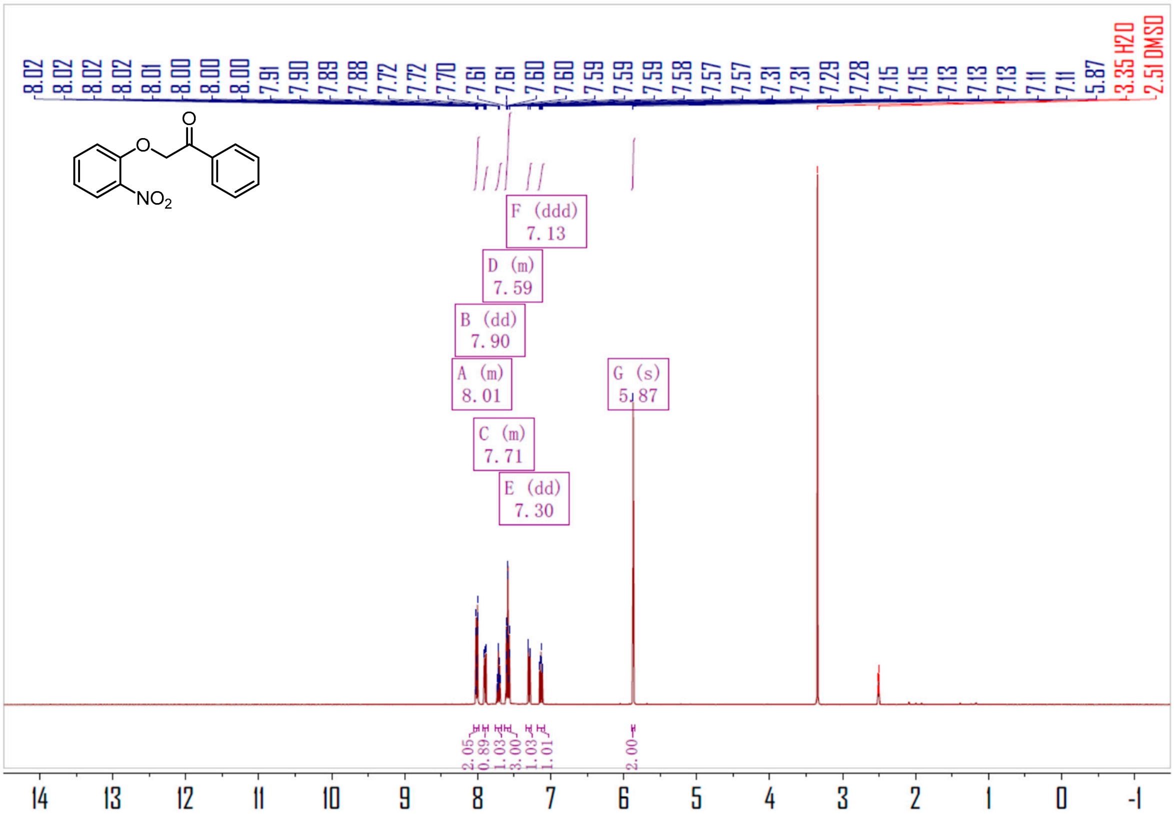

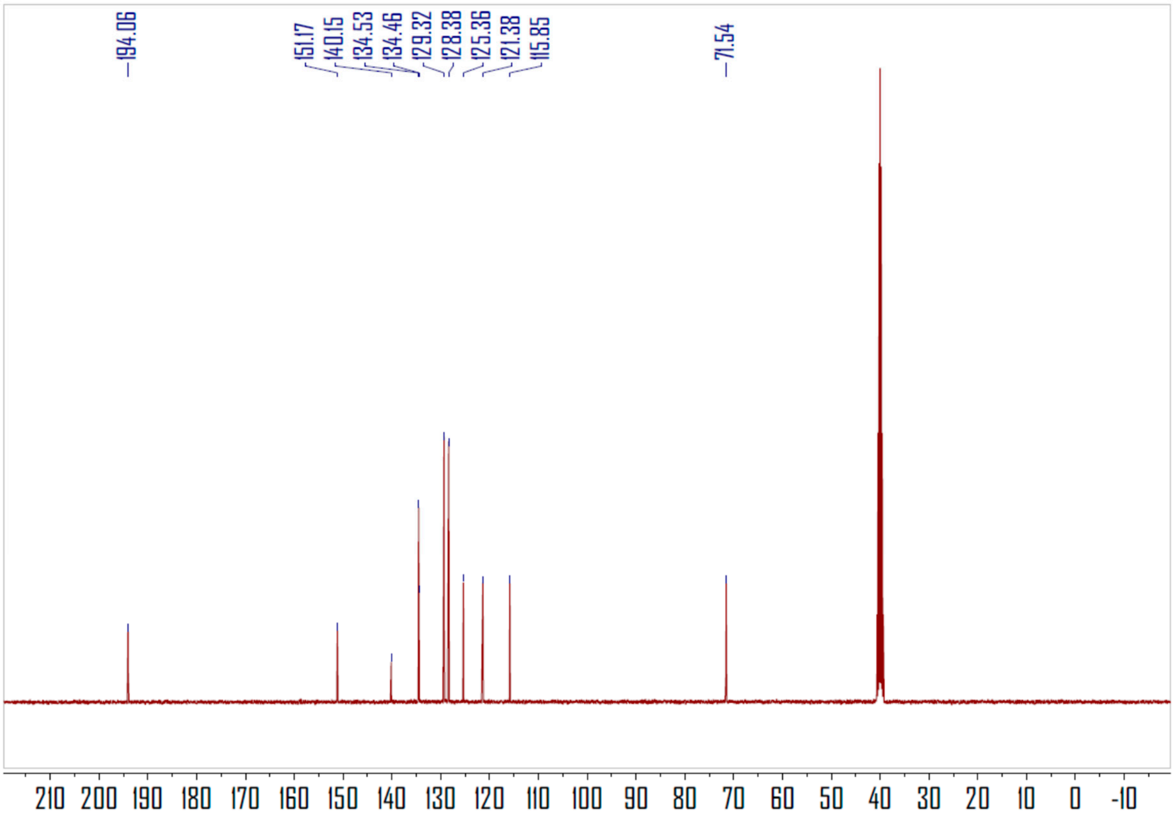

*1-(4-methoxyphenyl)-2-(2-nitrophenoxy)ethan-1-one (**3b**)*

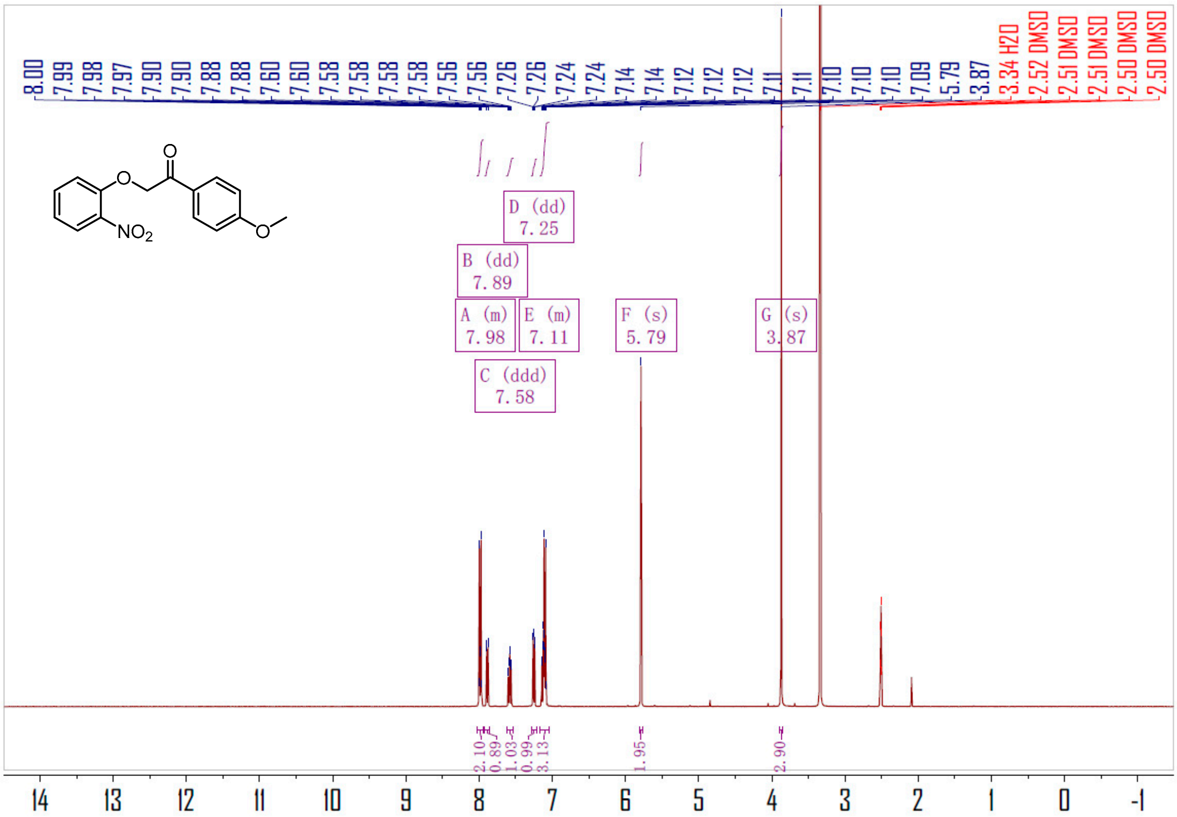

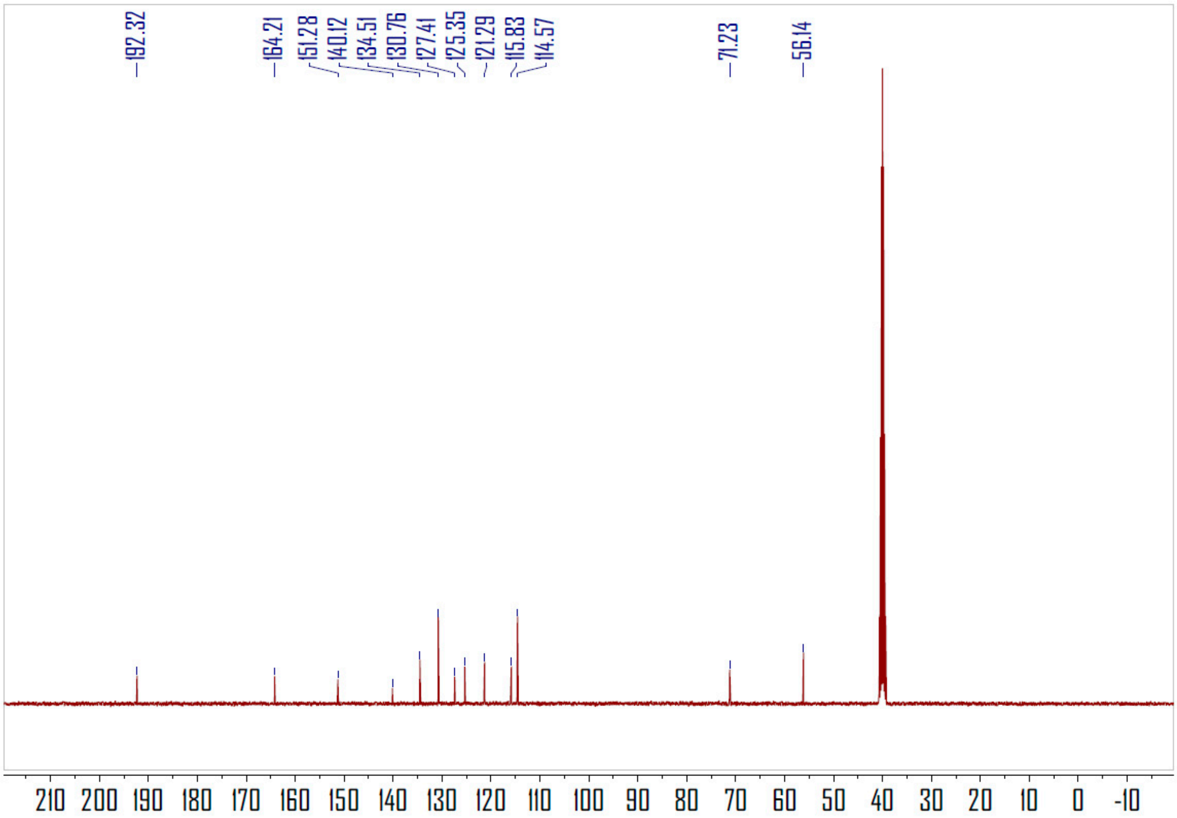

*2-(5-methoxy-2-nitrophenoxy)-1-(4-methoxyphenyl)ethan-1-one (**8a**)*

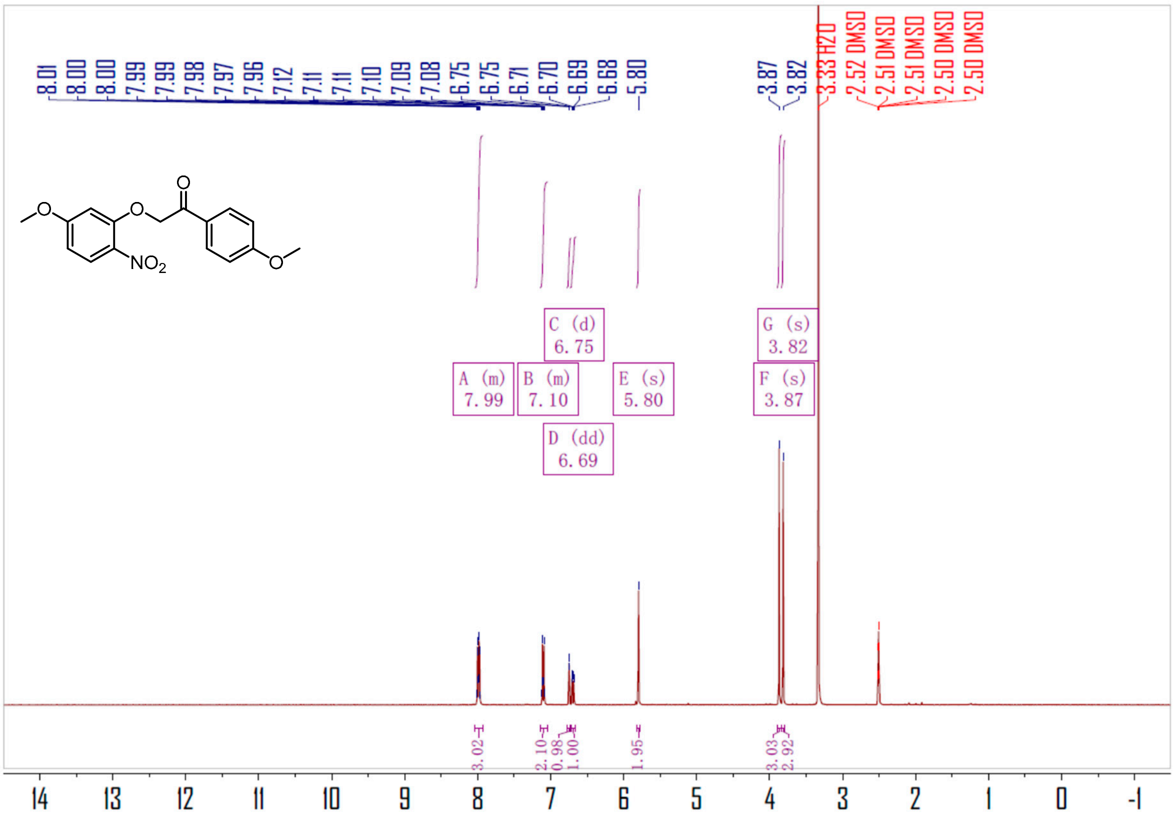

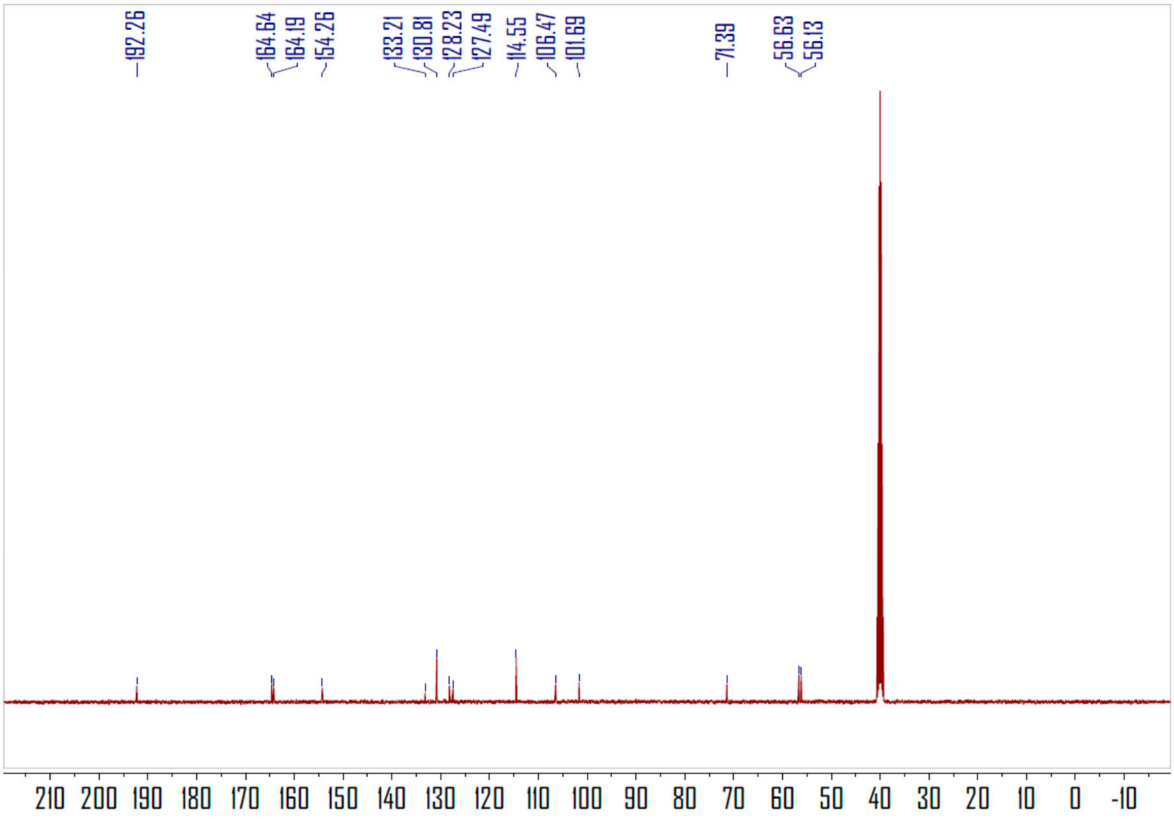

*1-(2,4-dimethylphenyl)-2-(5-methoxy-2-nitrophenoxy)ethan-1-one (**8b**)*

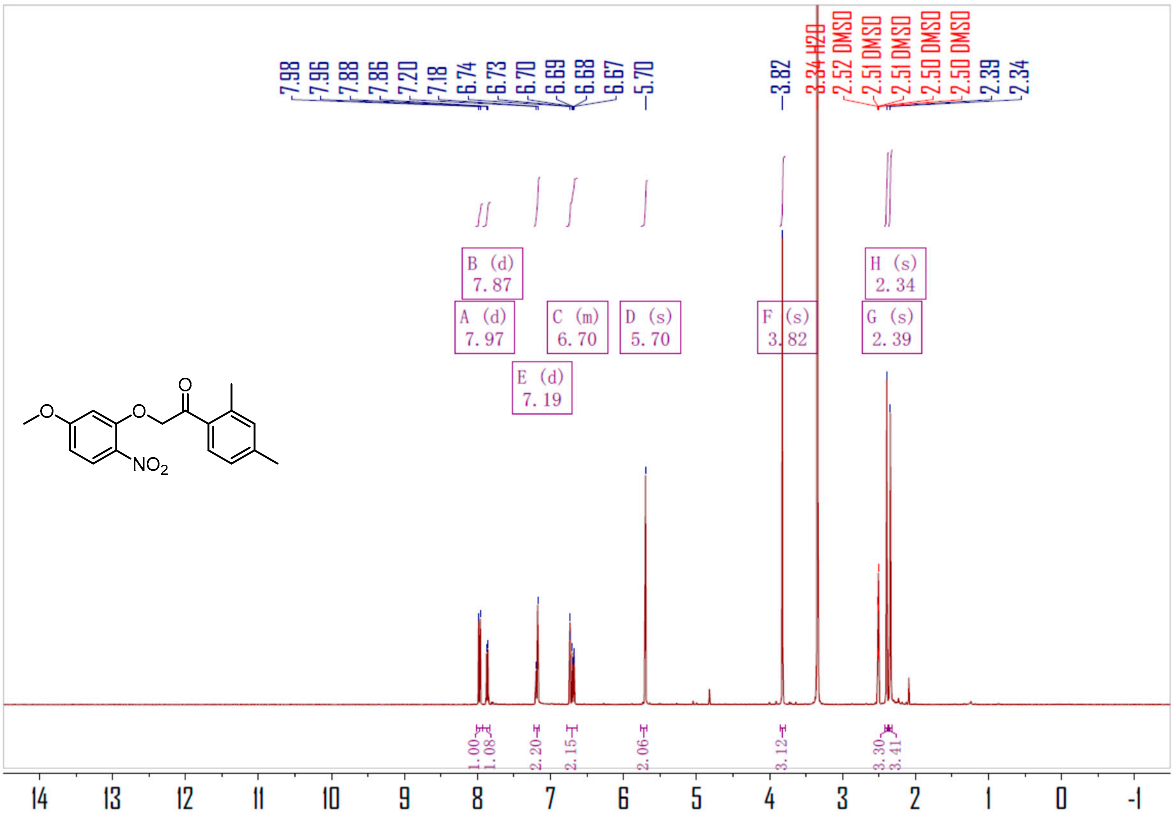

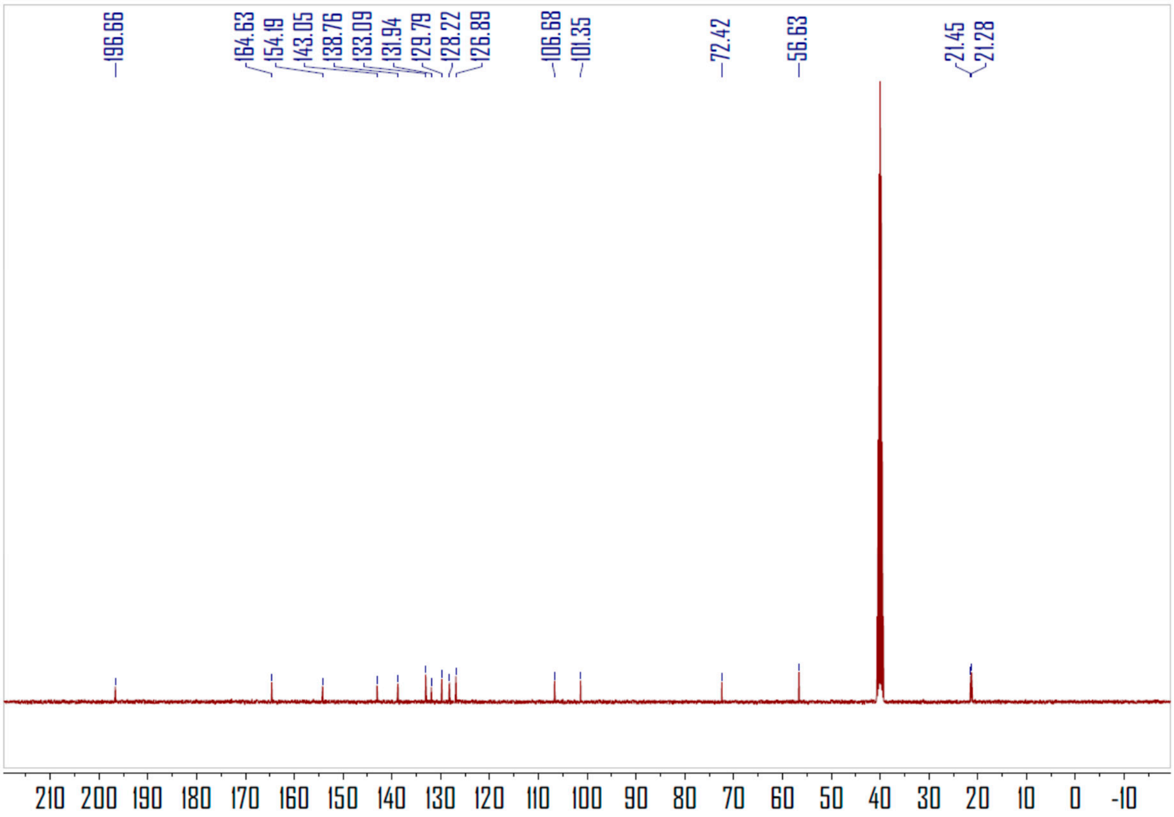

*1-(4-fluorophenyl)-2-(5-methoxy-2-nitrophenoxy)ethan-1-one (**8c**)*

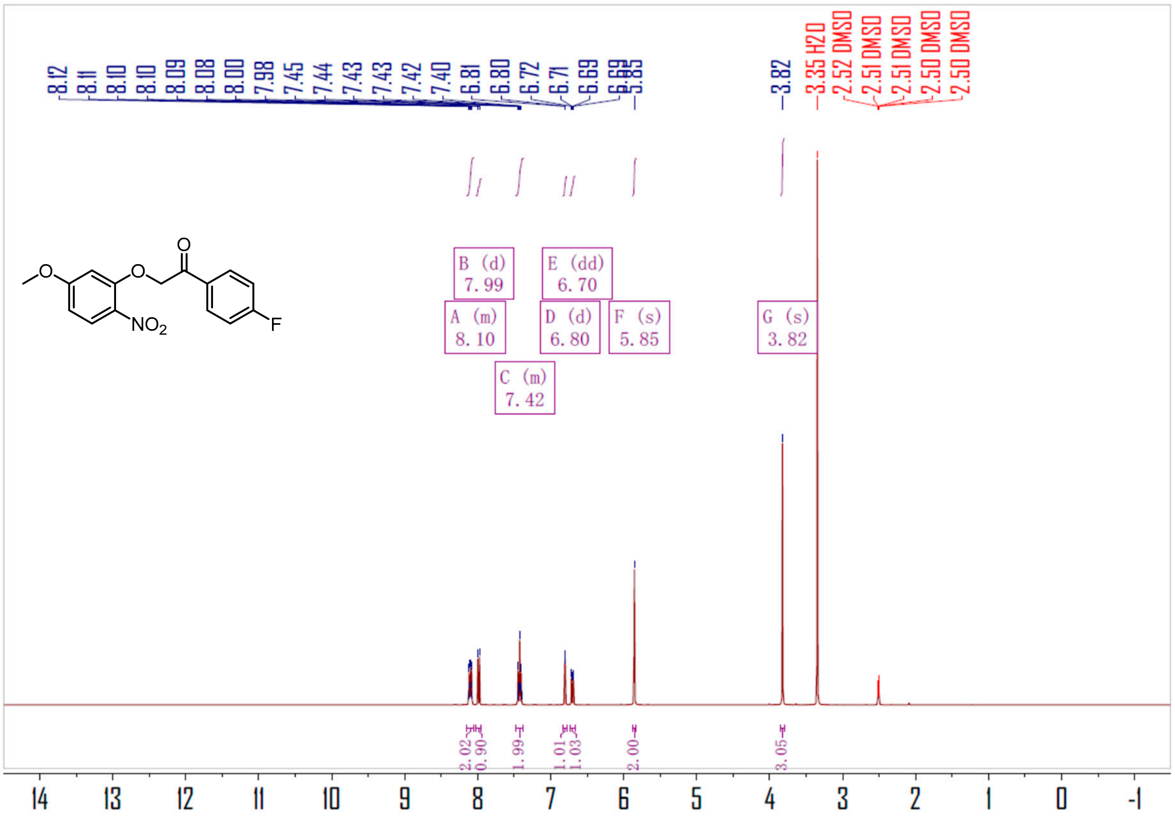

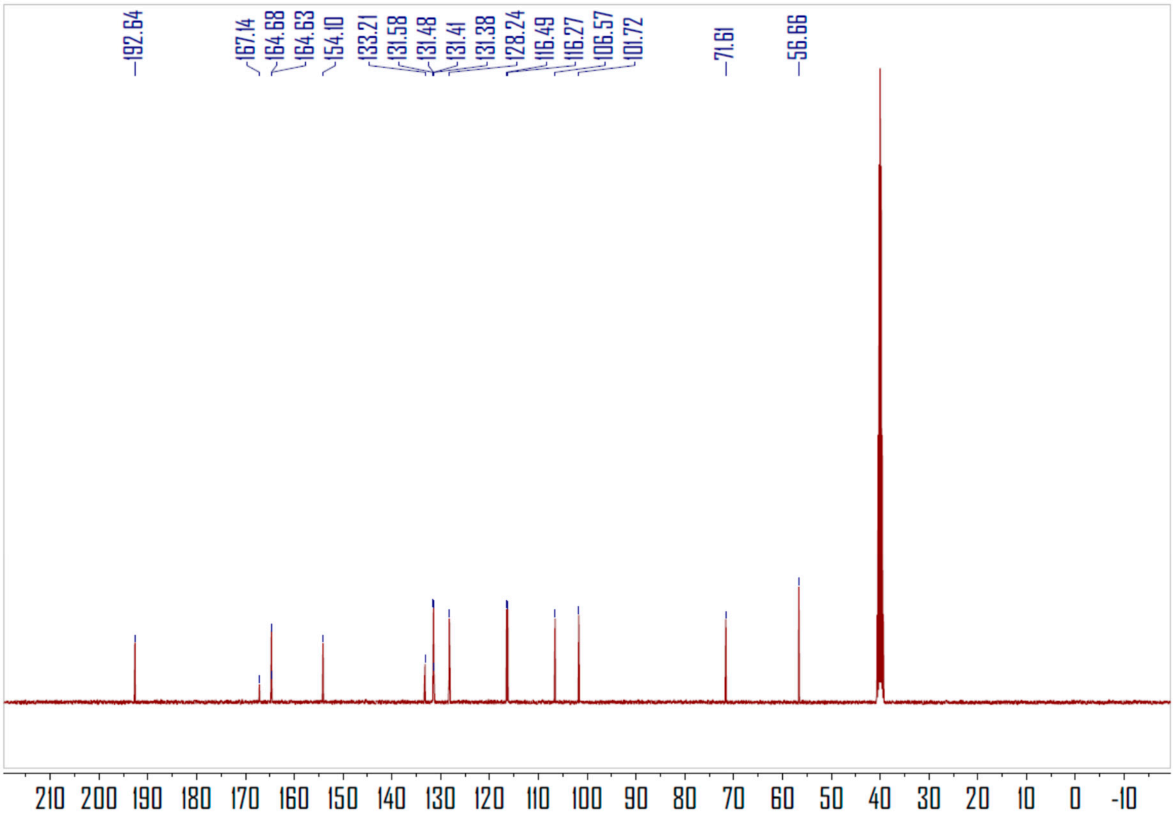

*3-phenyl-3,4-dihydro-2H-benzo[b][1,4]oxazine (**4a**)*

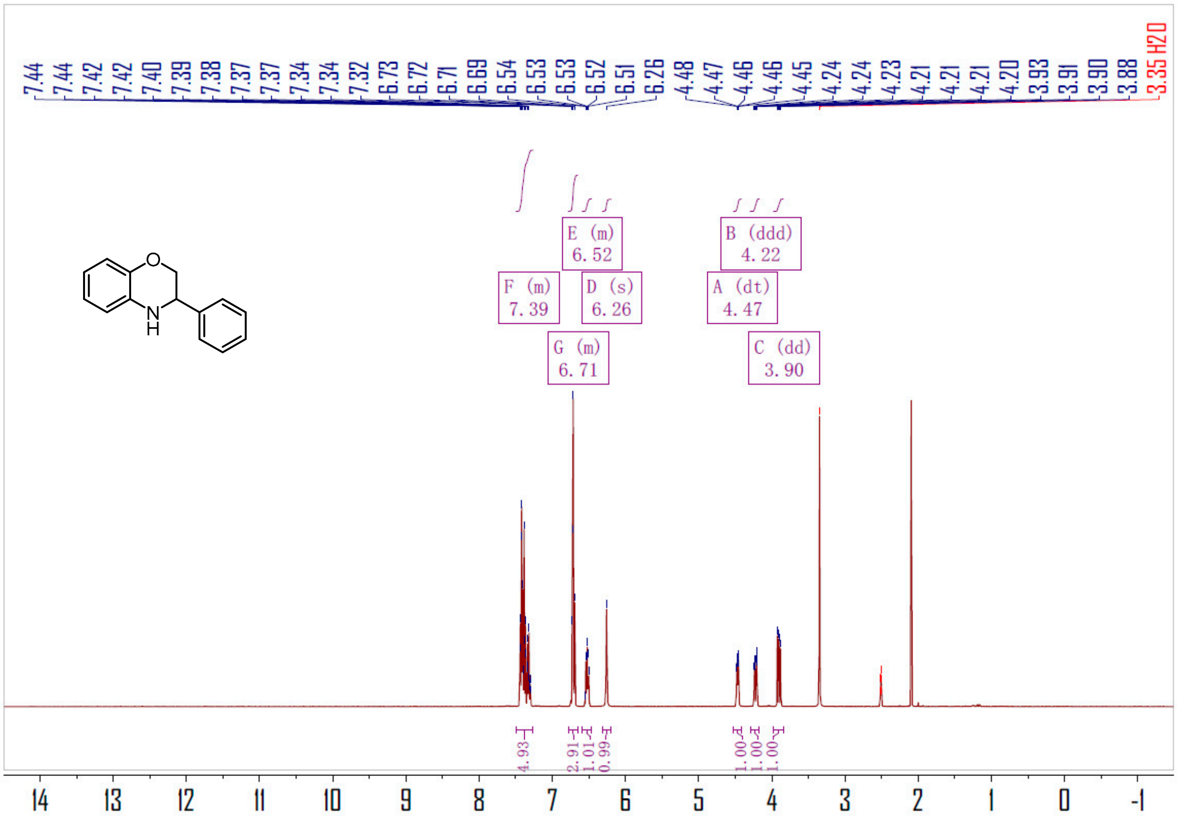

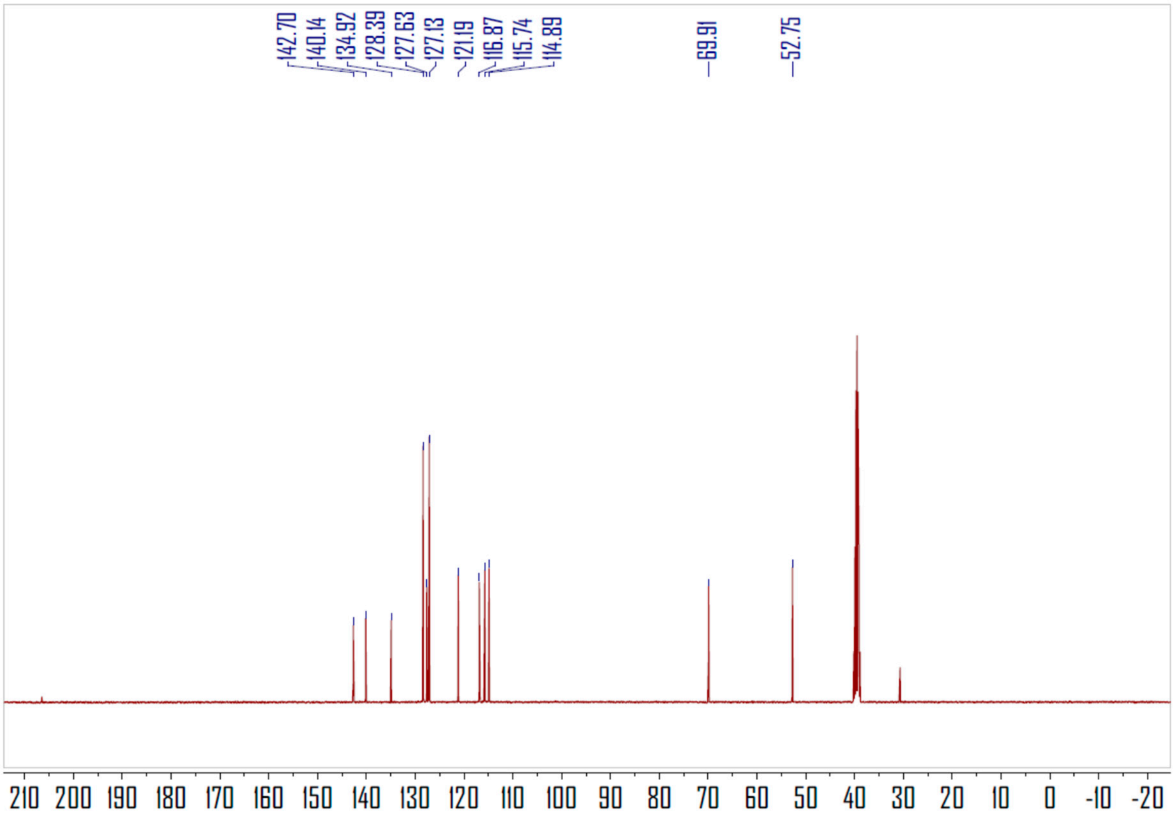

*3-(4-methoxyphenyl)-3,4-dihydro-2H-benzo[b][1,4]oxazine (**4b**)*

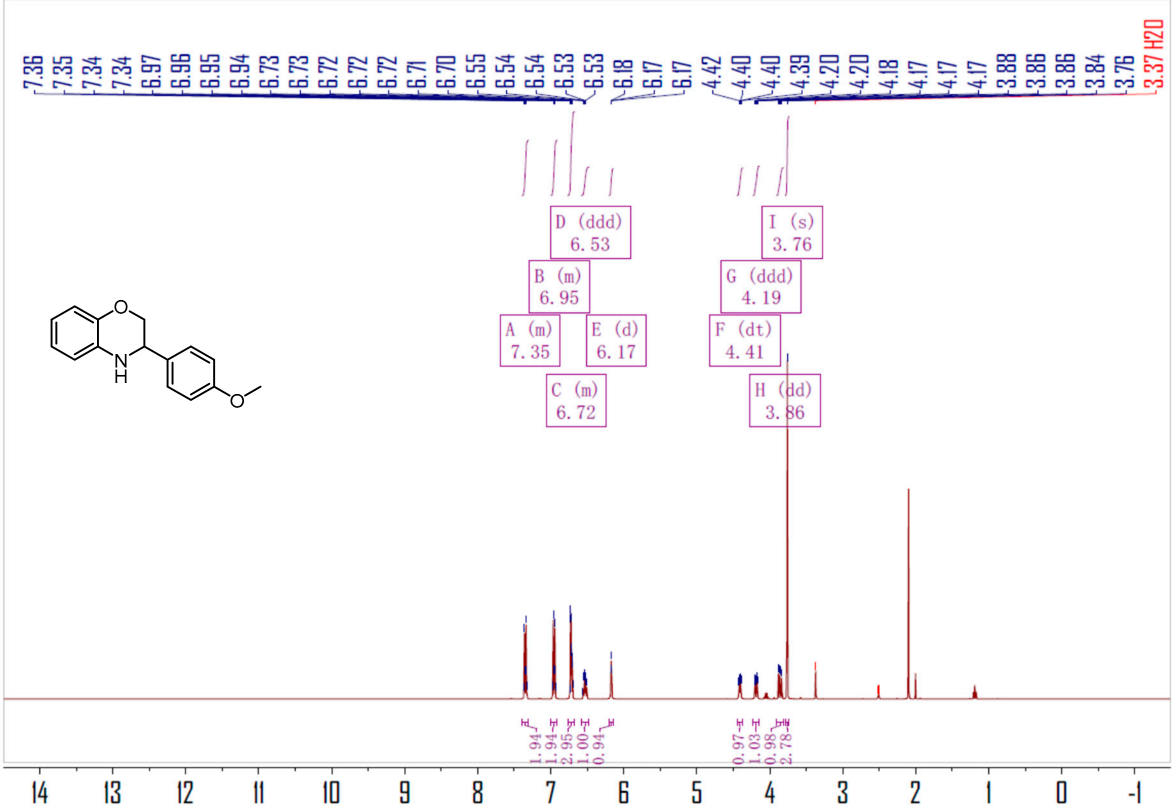

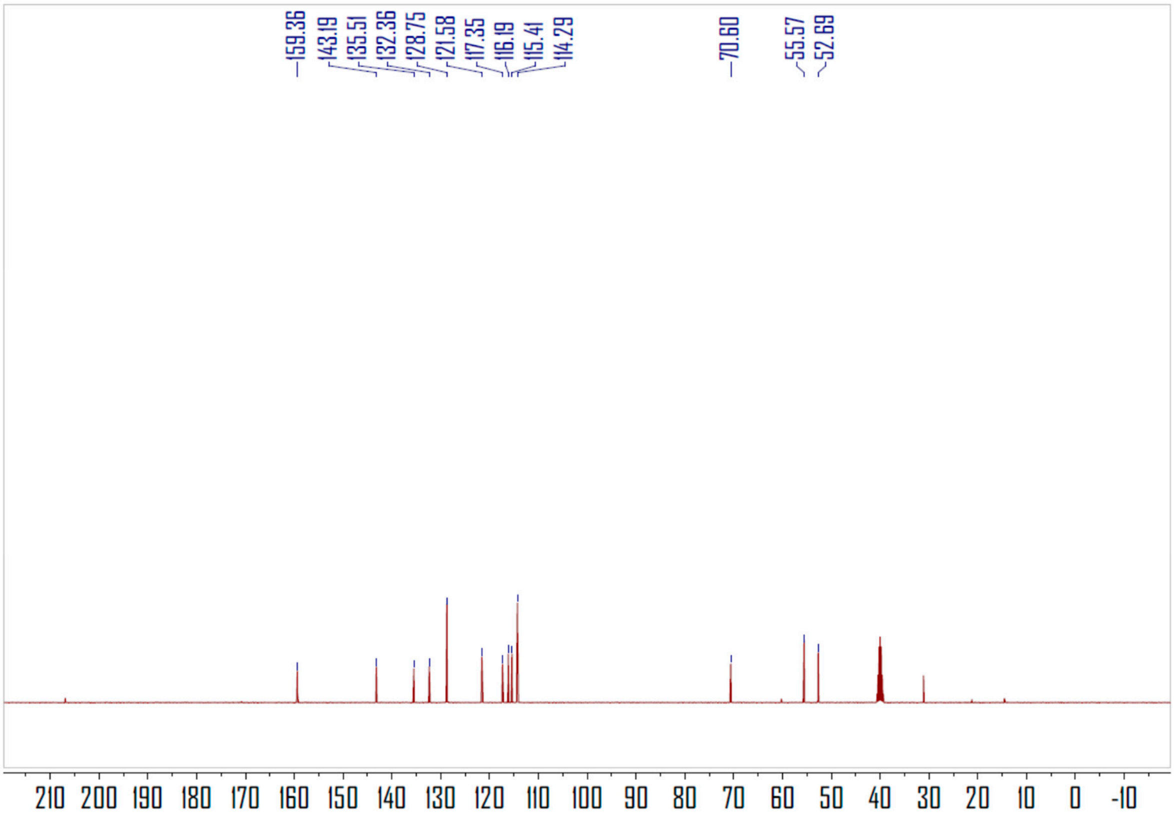

*7-methoxy-3-(4-methoxyphenyl)-3,4-dihydro-2H-benzo[b][1,4]oxazine (**9a**)*

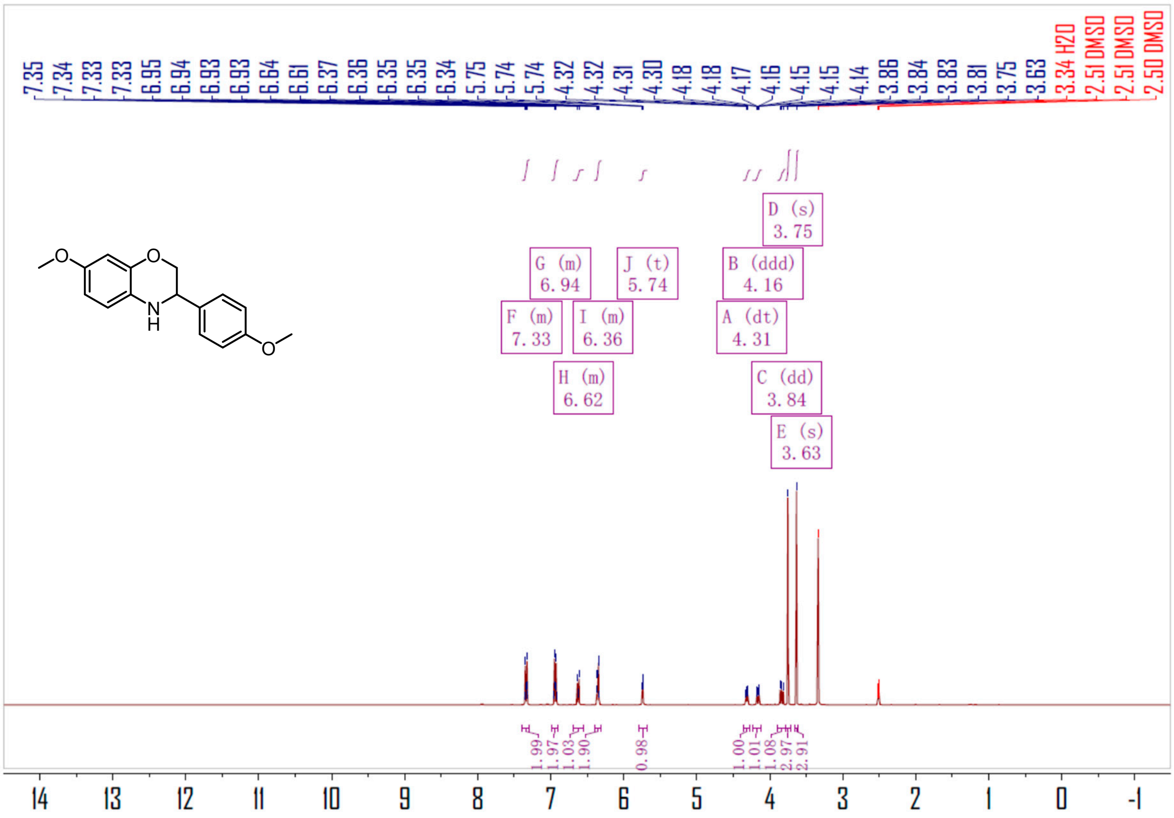

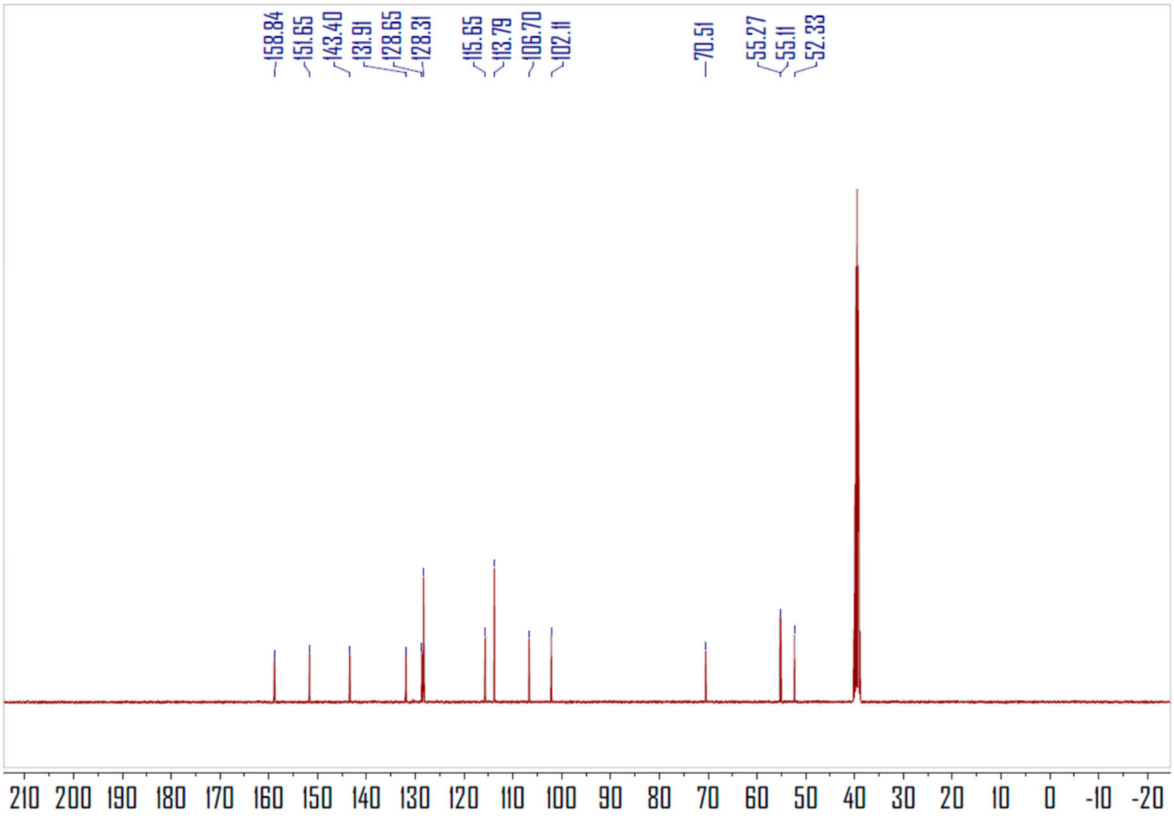

*3-(2,4-dimethylphenyl)-7-methoxy-3,4-dihydro-2H-benzo[b][1,4]oxazine (**9b**)*

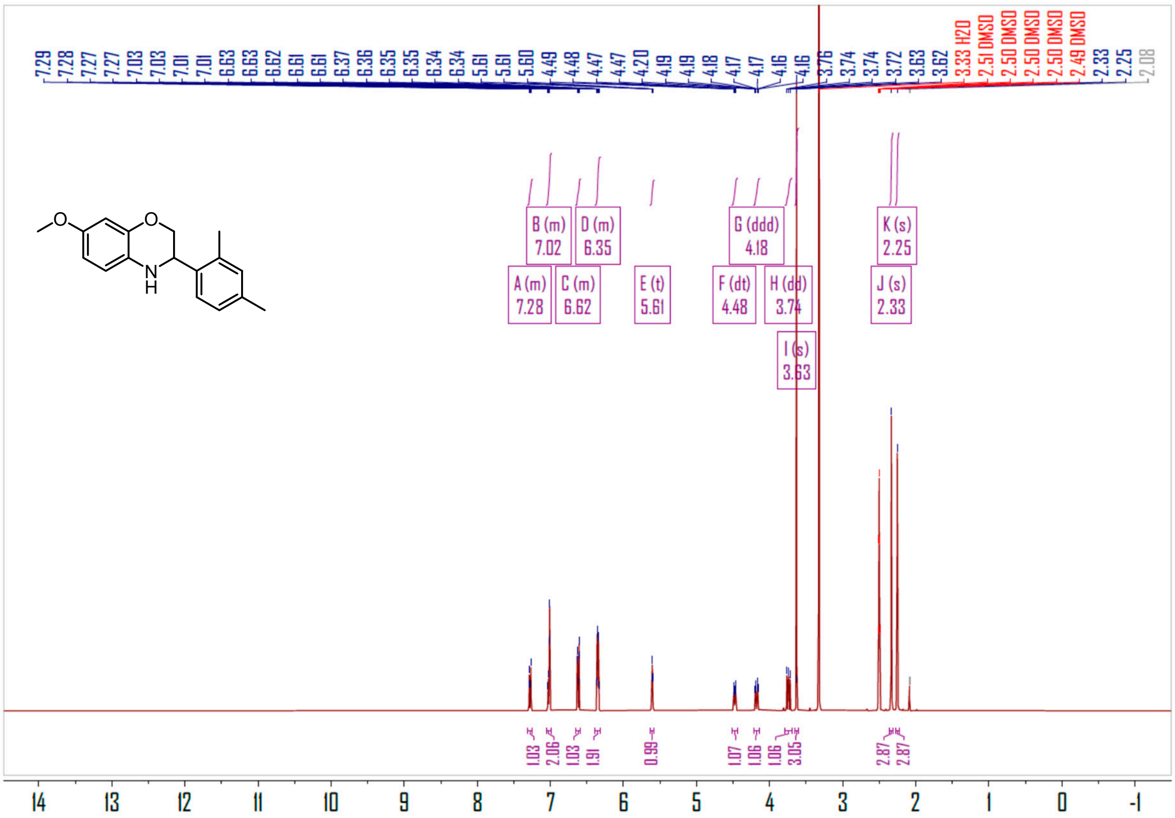

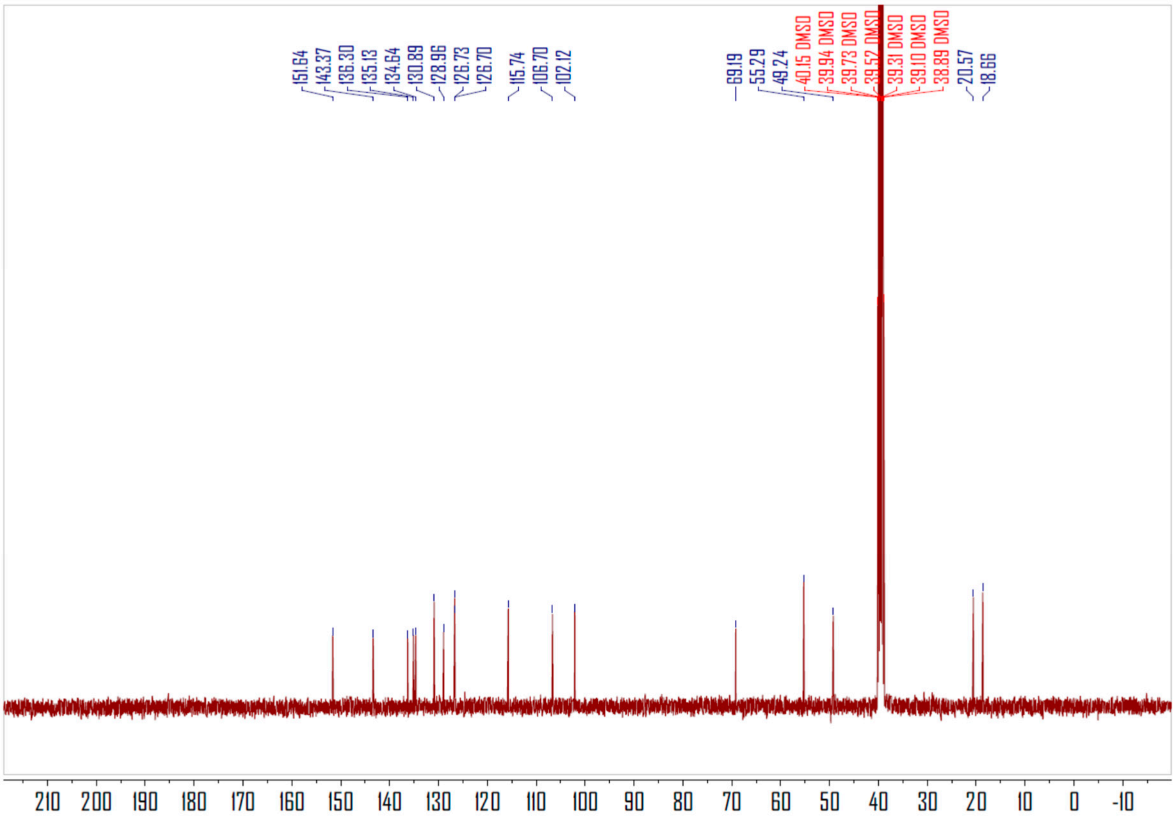

*3-(4-fluorophenyl)-7-methoxy-3,4-dihydro-2H-benzo[b][1,4]oxazine (**9c**)*

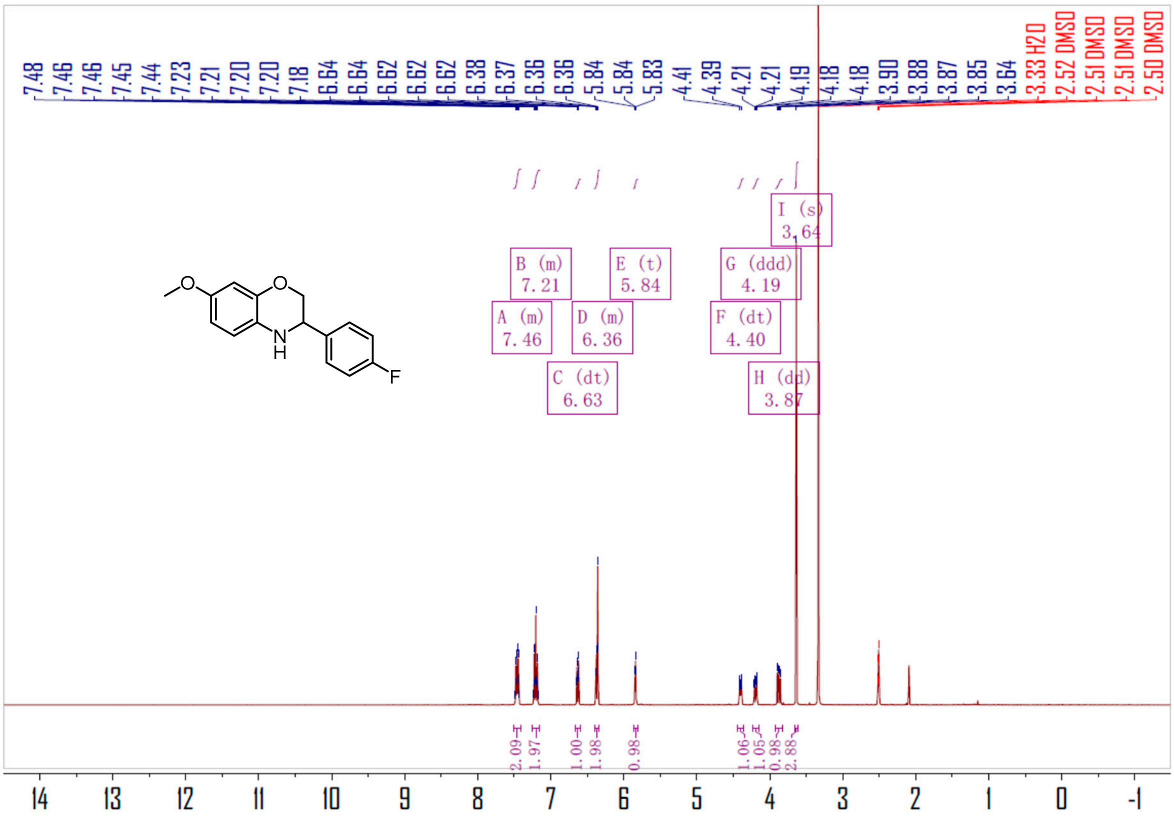

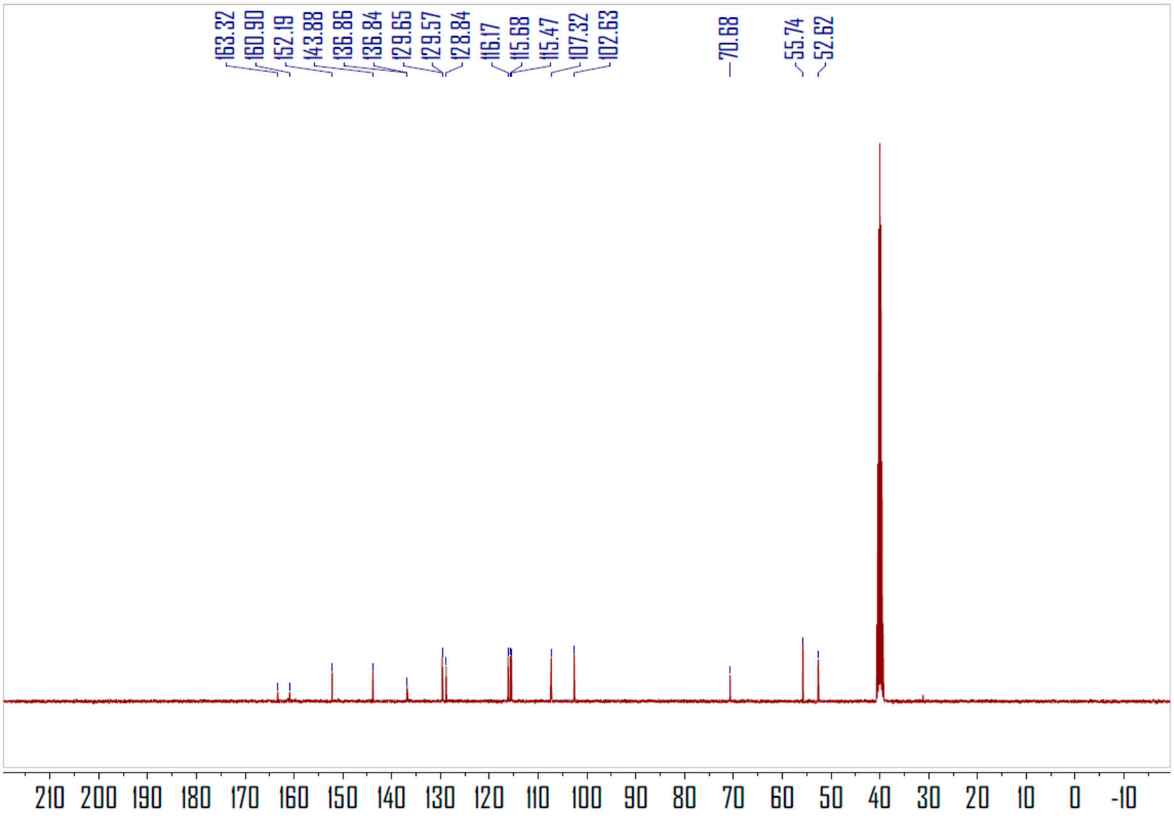

*7-methoxy-3-(4-methoxyphenyl)-2H-benzo[b][1,4]oxazine (**10a**)*

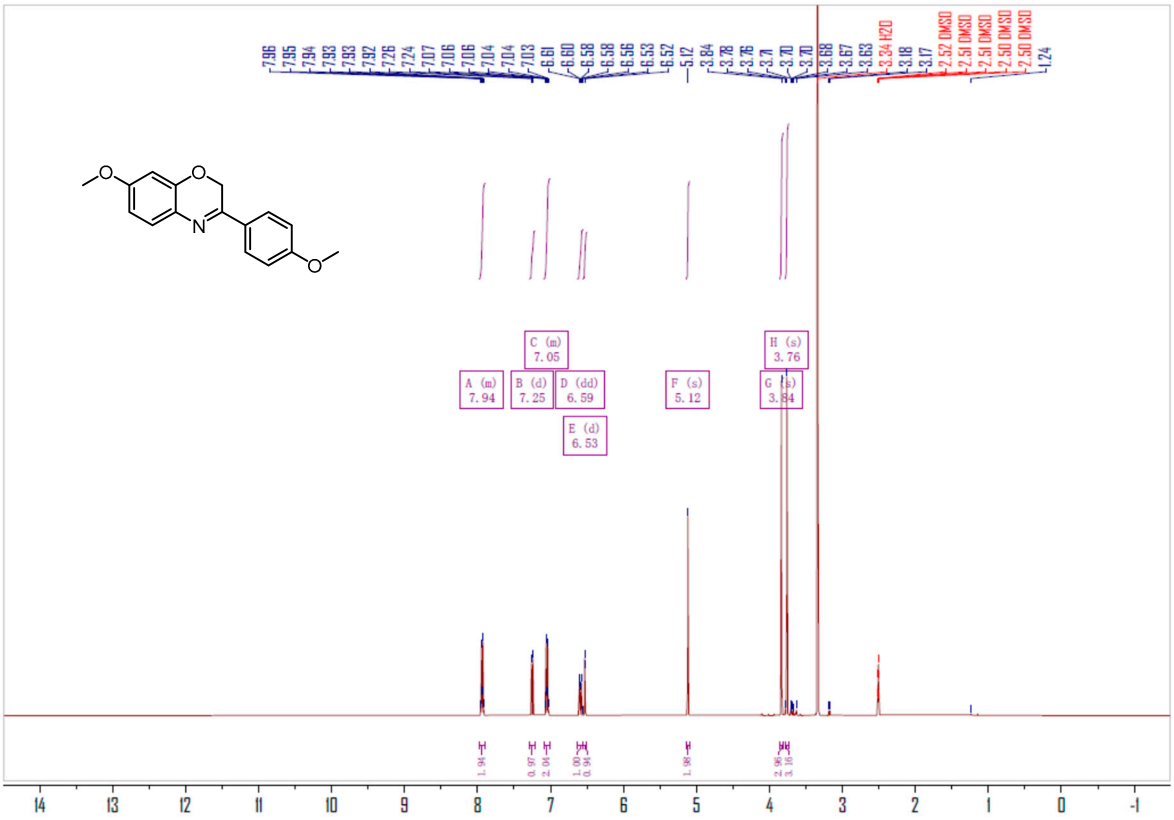

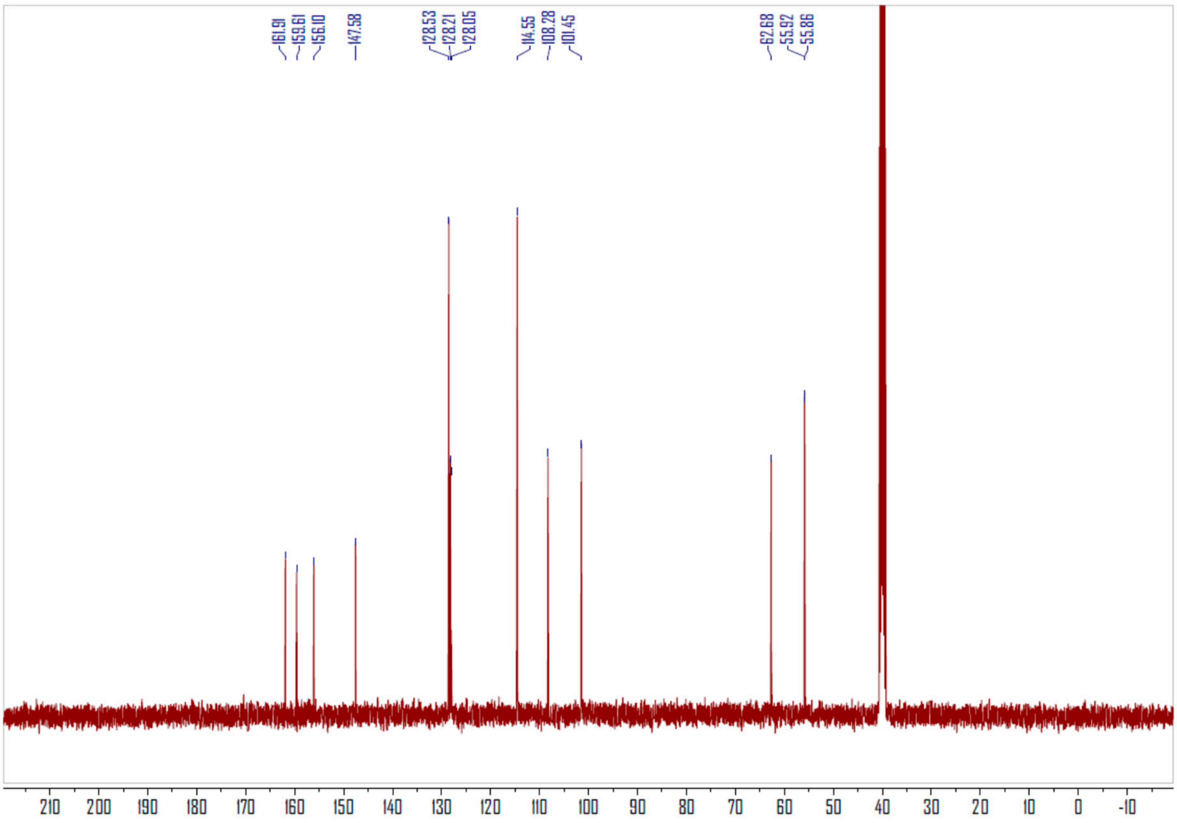

*3-(4-fluorophenyl)-7-methoxy-2H-benzo[b][1,4]oxazine (**10b**)*

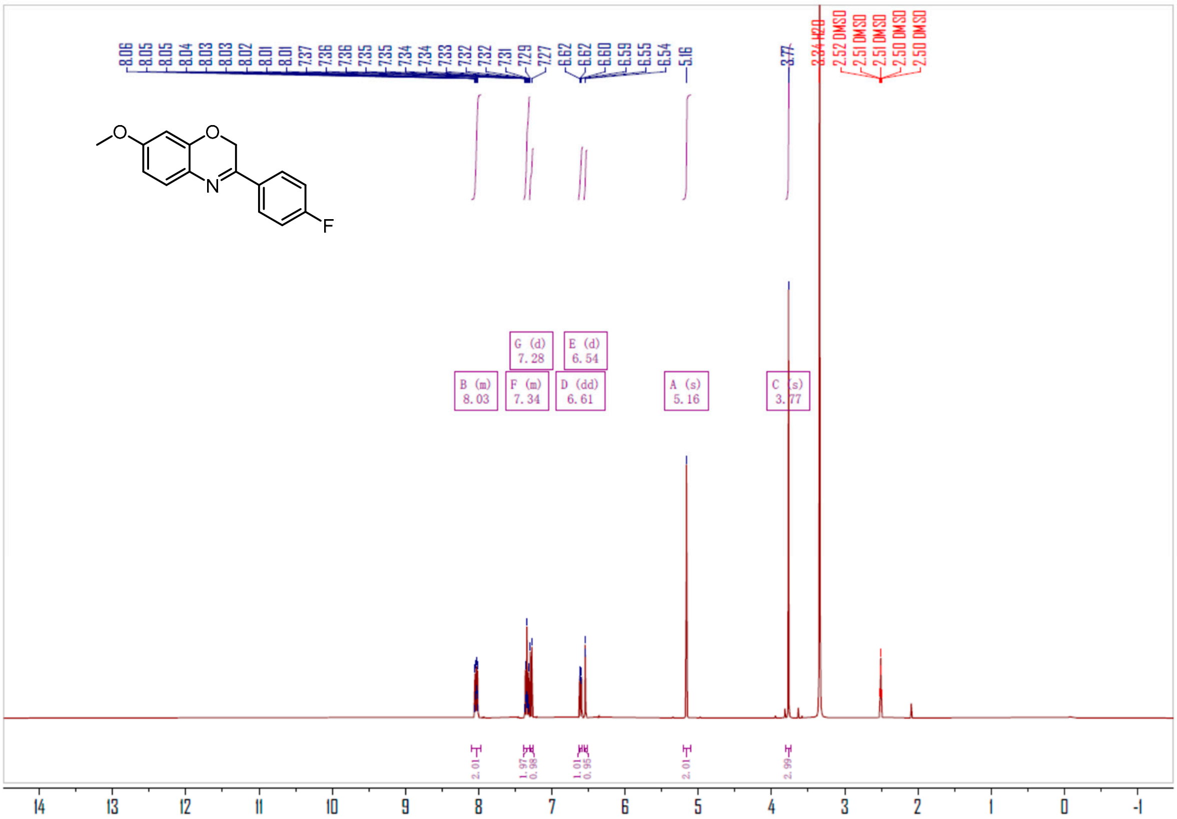

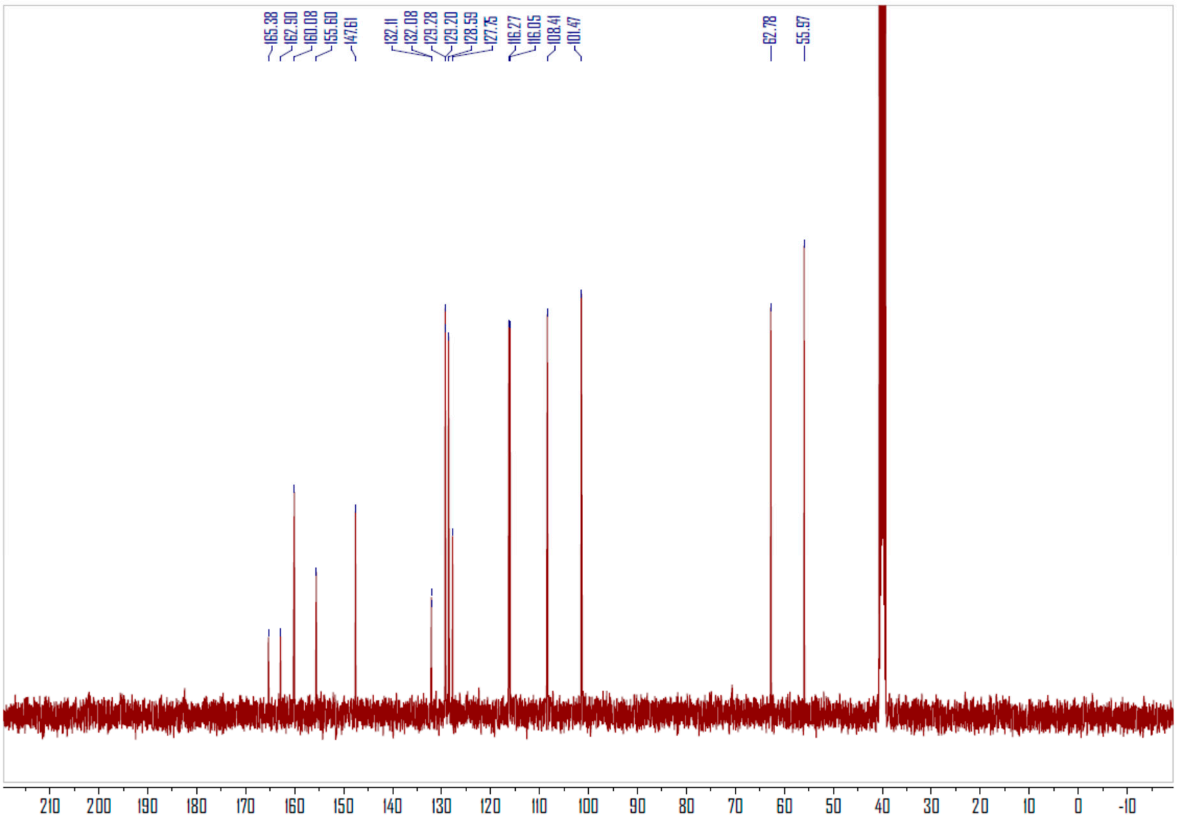

*3,4-diphenyl-3,4-dihydro-2H-benzo[b][1,4]oxazine (**5a**)*

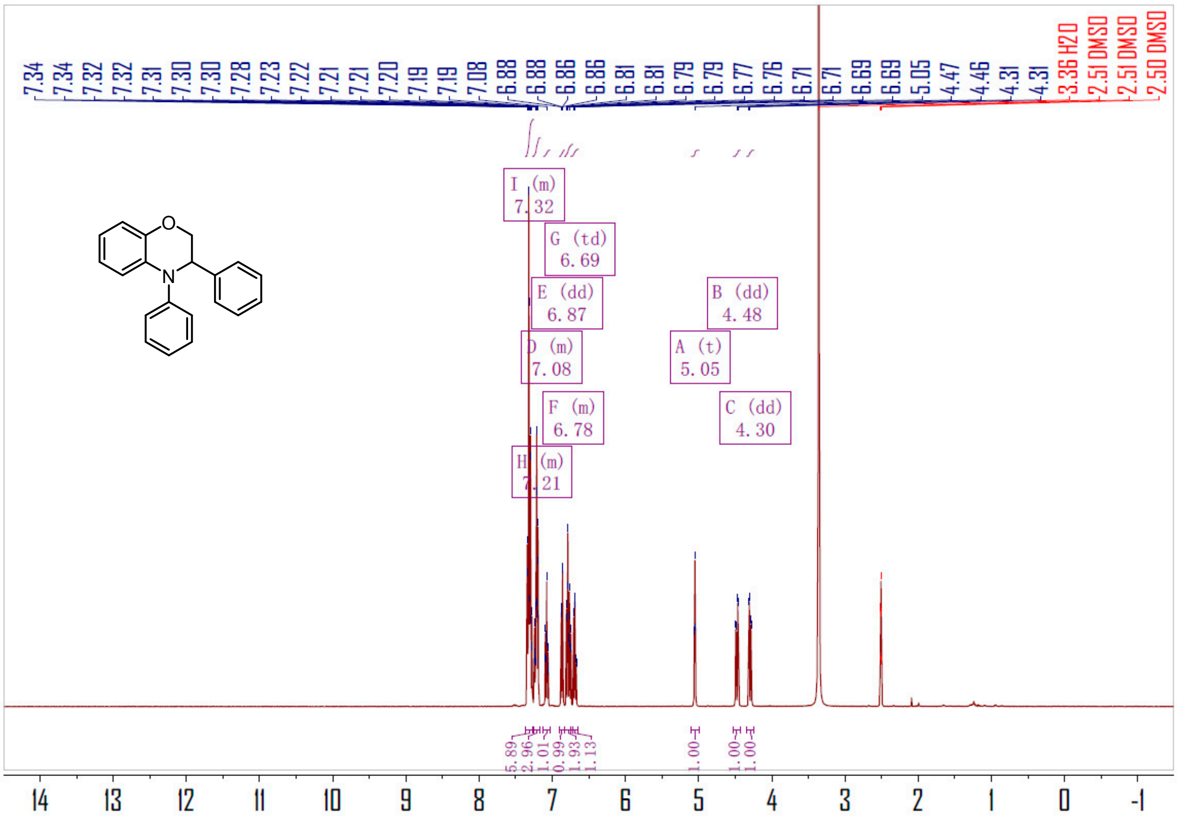

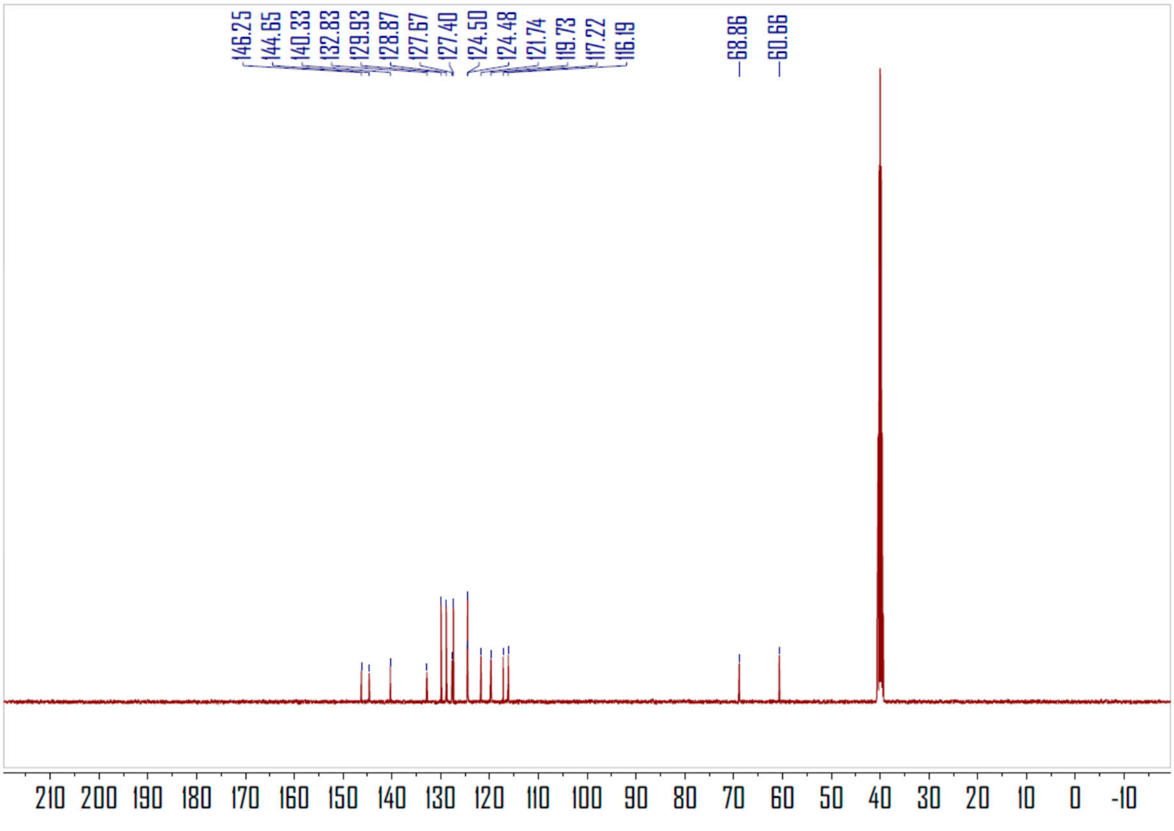

*4-(4-nitrophenyl)-3-phenyl-3,4-dihydro-2H-benzo[b][1,4]oxazine (**5b**)*

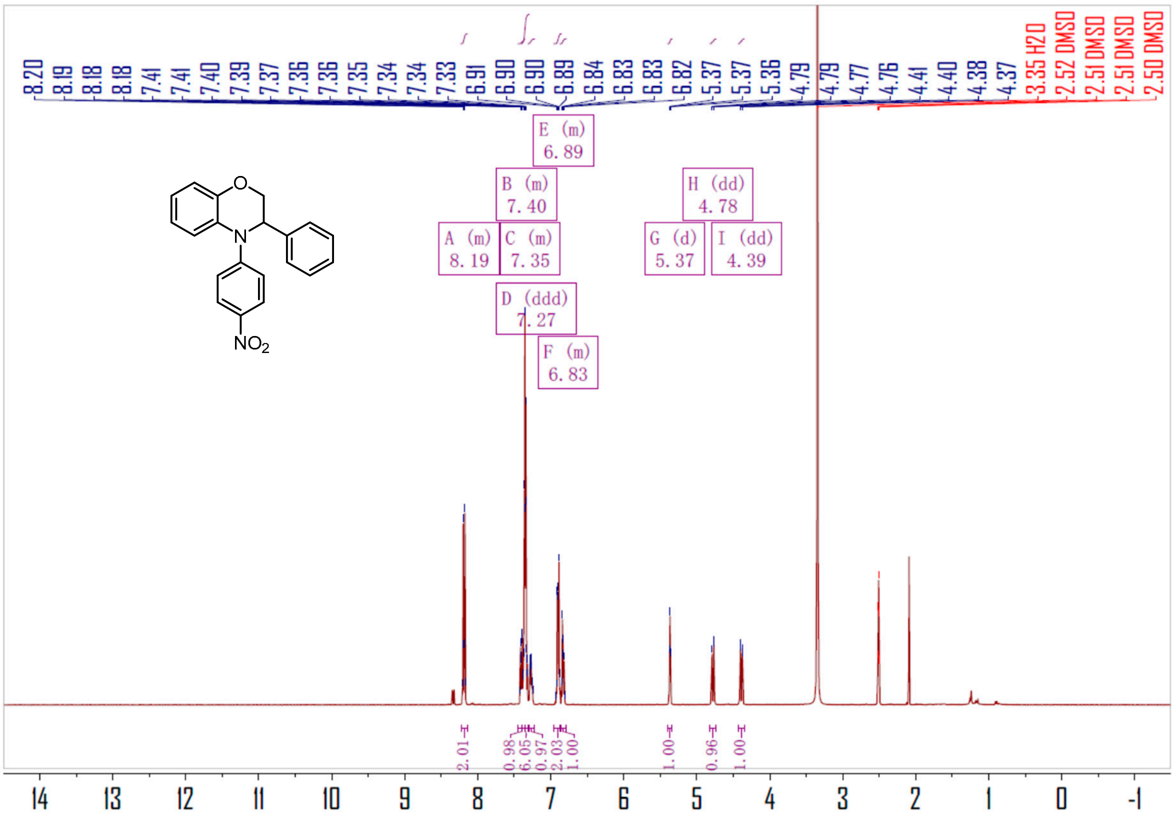

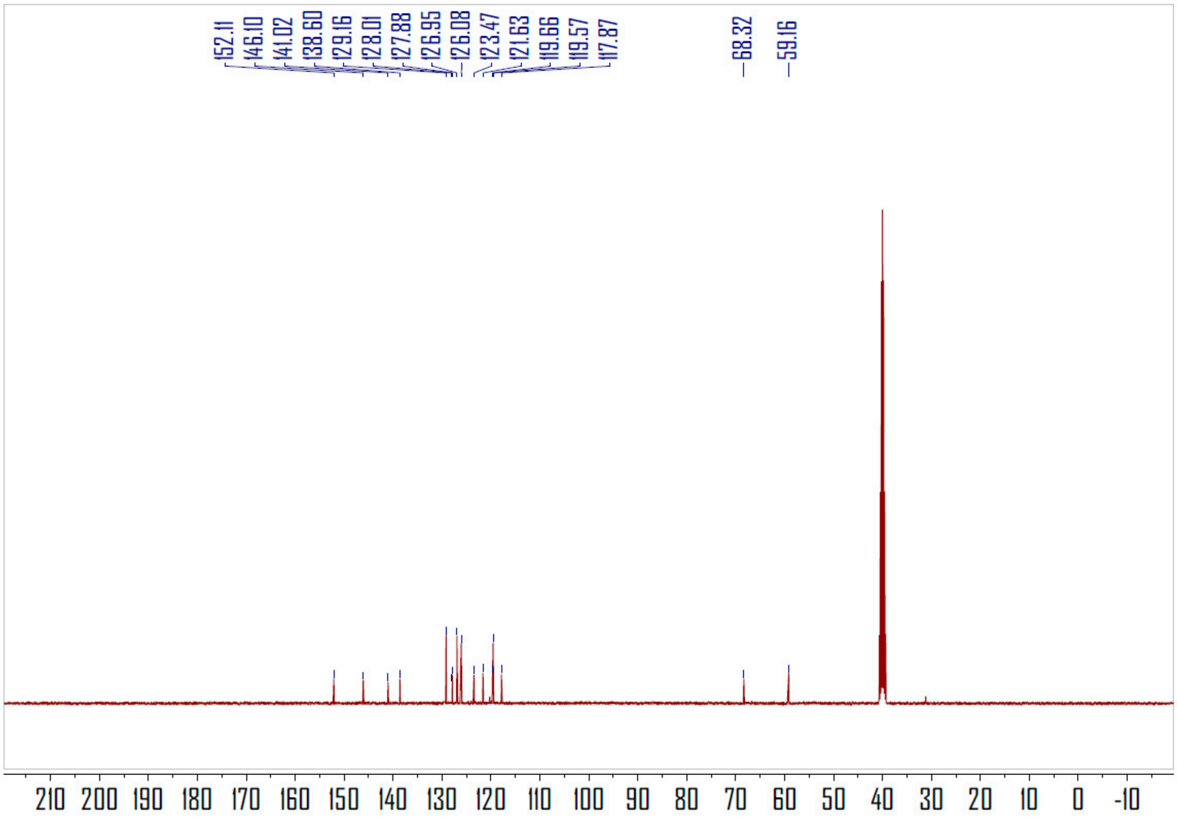

*3-(4-methoxyphenyl)-4-phenyl-3,4-dihydro-2H-benzo[b][1,4]oxazine (**5c**)*

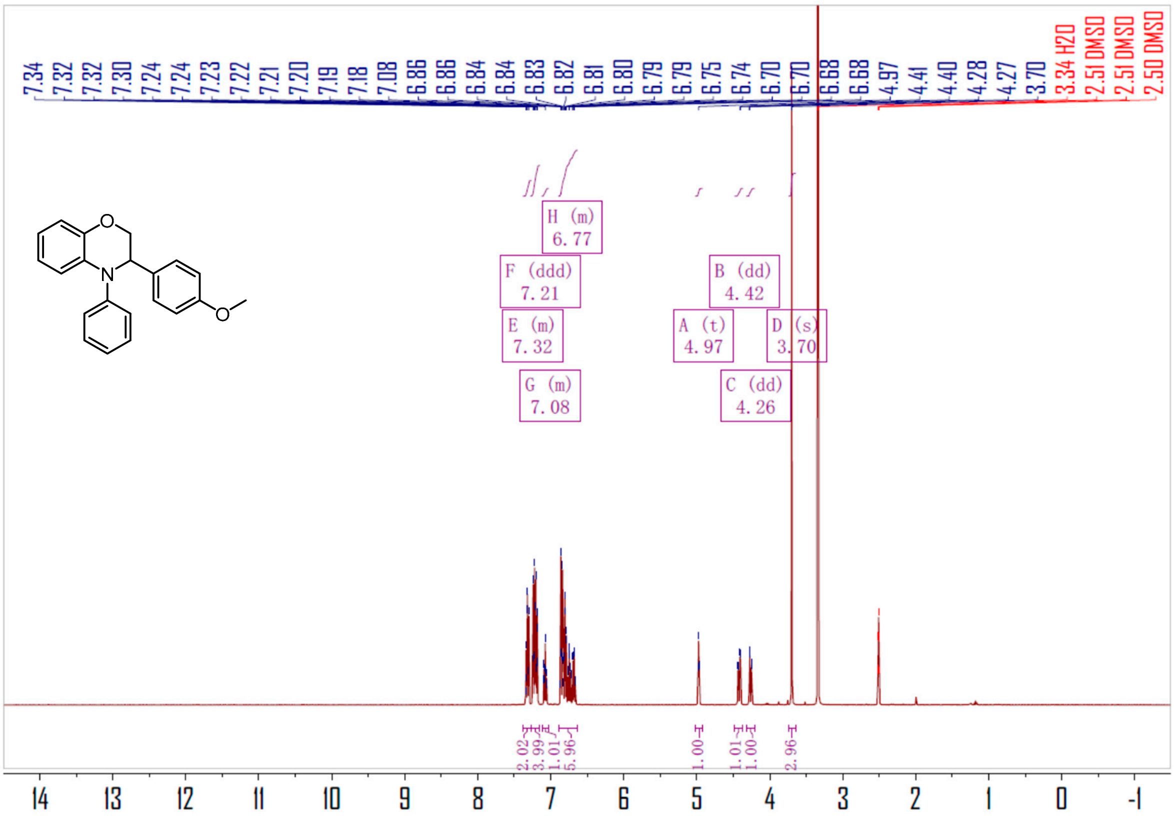

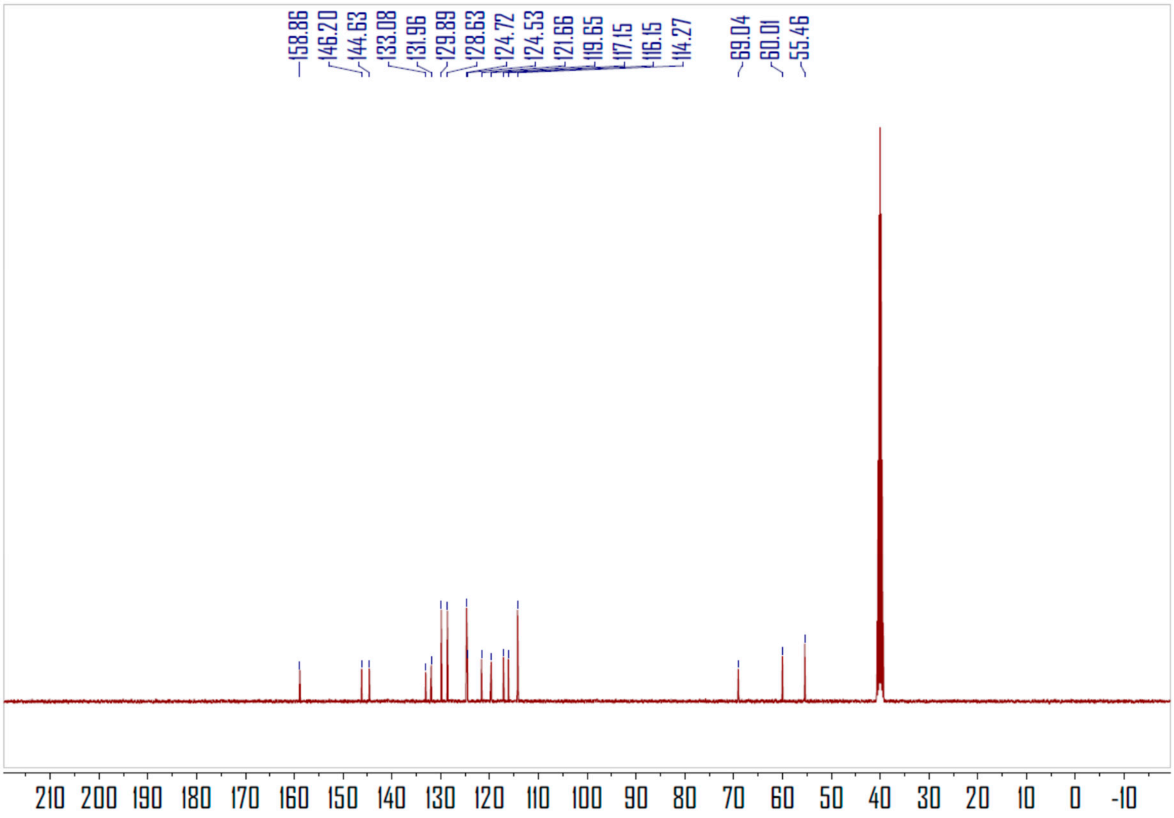

*3-(4-methoxyphenyl)-4-(4-nitrophenyl)-3,4-dihydro-2H-benzo[b][1,4]oxazine (**5d**)*

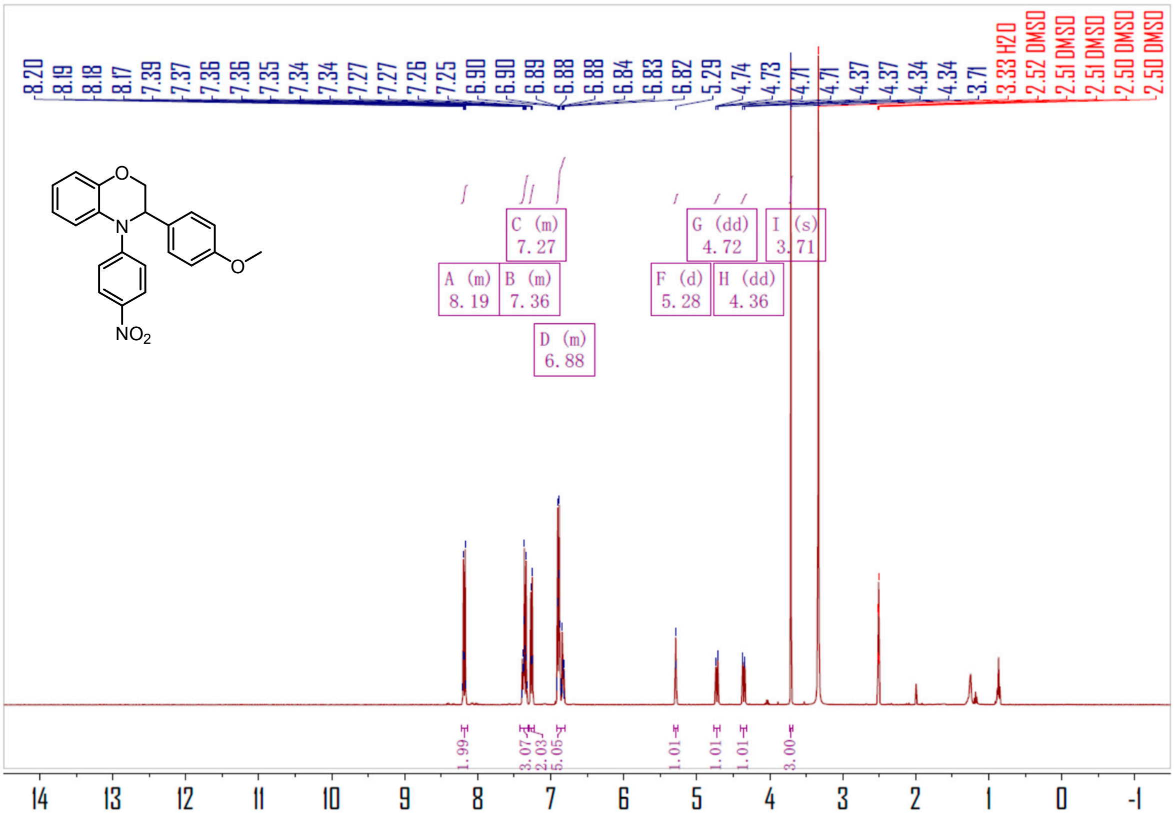

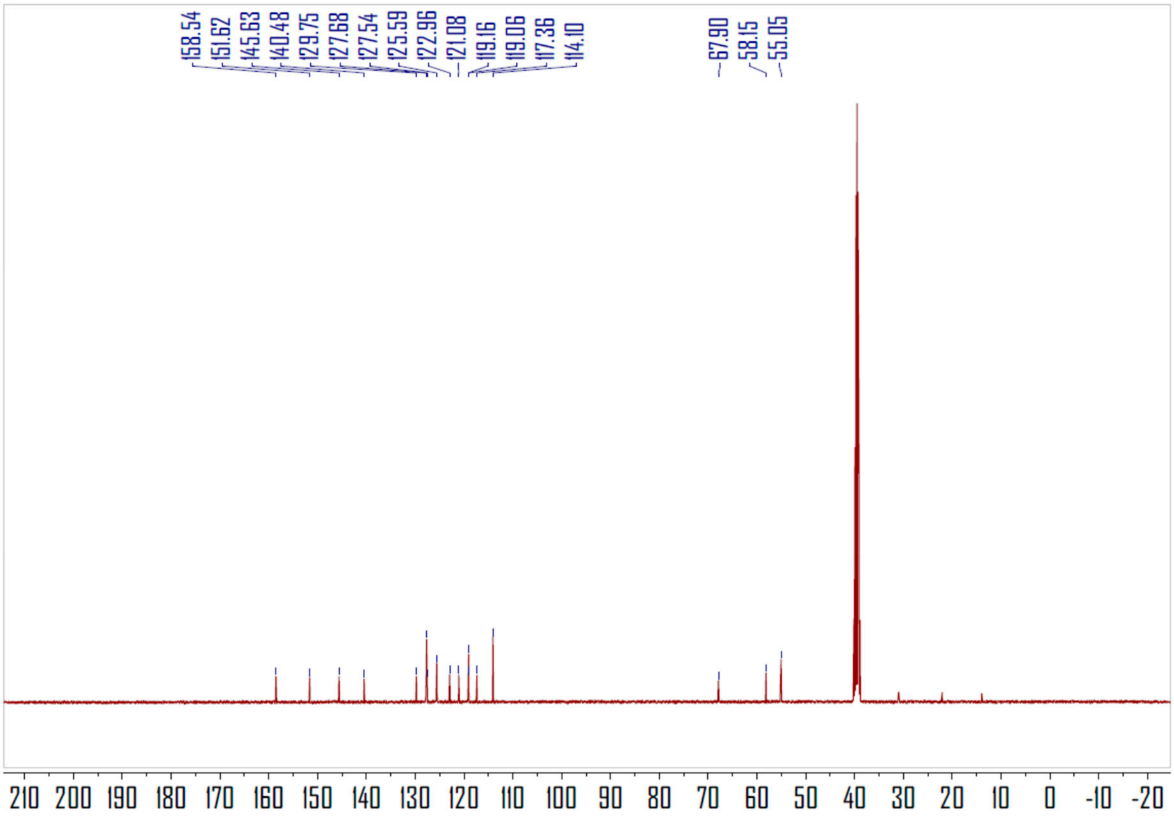

*4-(4-fluorophenyl)-3-(4-methoxyphenyl)-3,4-dihydro-2H-benzo[b][1,4]oxazine (**5e**)*

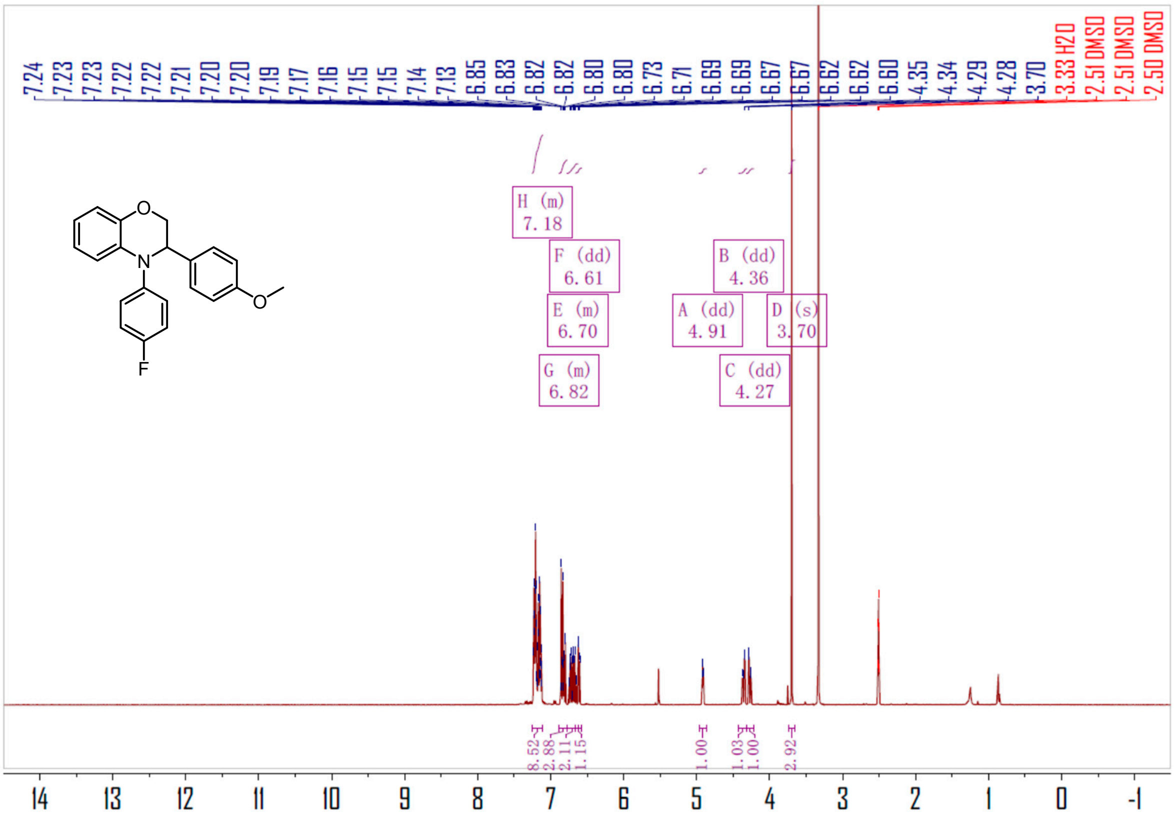

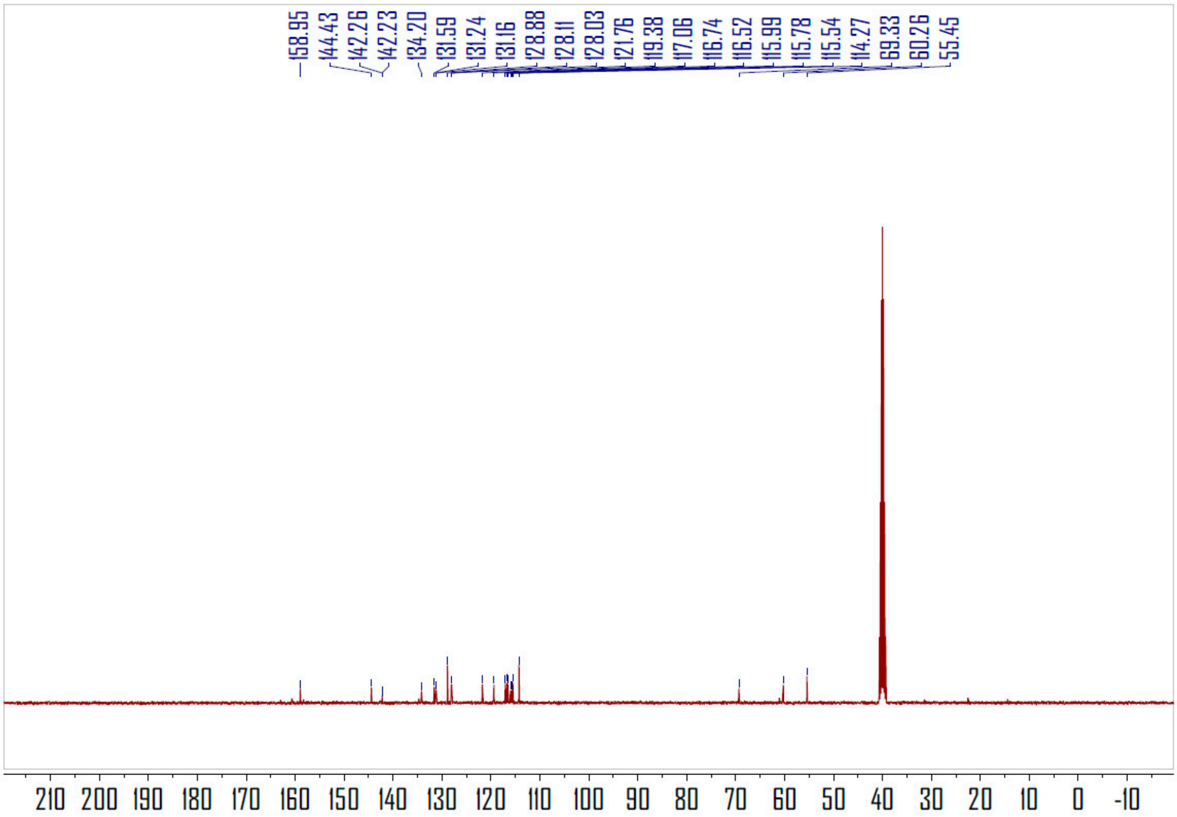

7-methoxy-3-(4-methoxyphenyl)-4-phenyl-3,4-dihydro-2H-benzo[b][1,4]oxazine (**11a**)

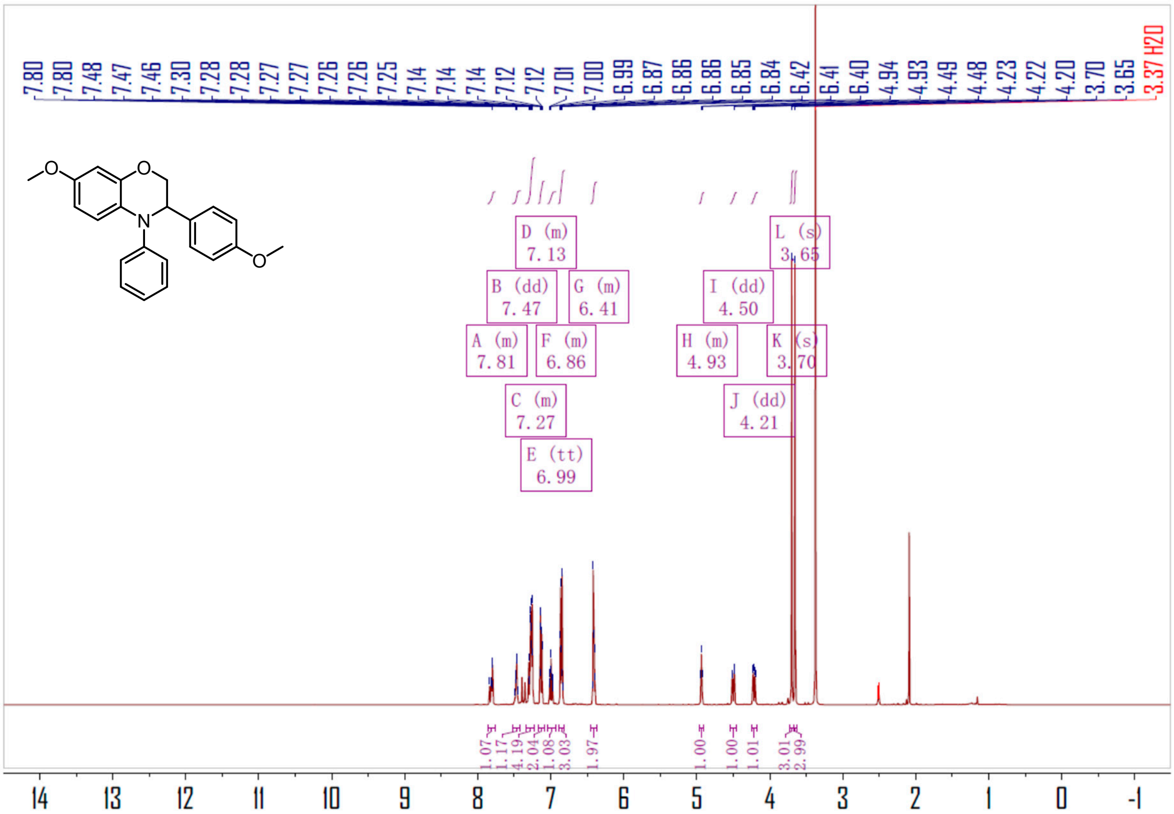

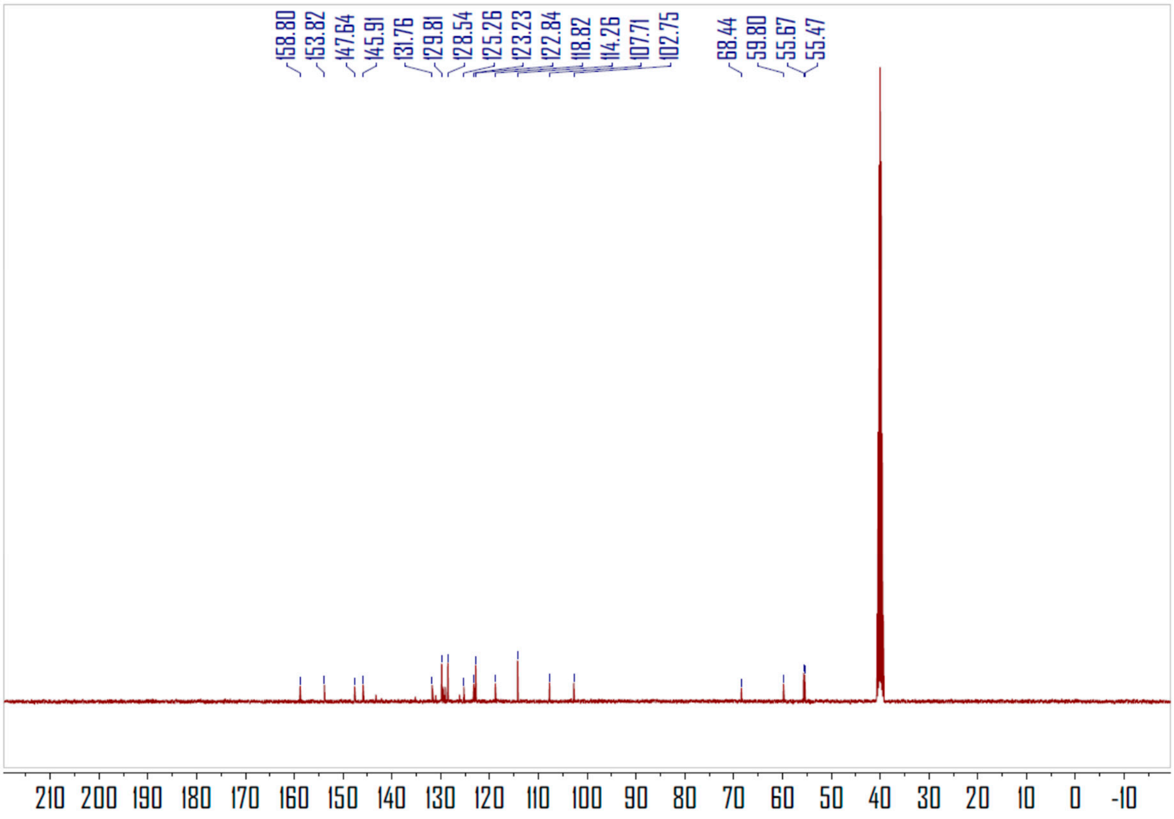

*7-methoxy-3,4-bis(4-methoxyphenyl)-3,4-dihydro-2H-benzo[b][1,4]oxazine (**11b**).*

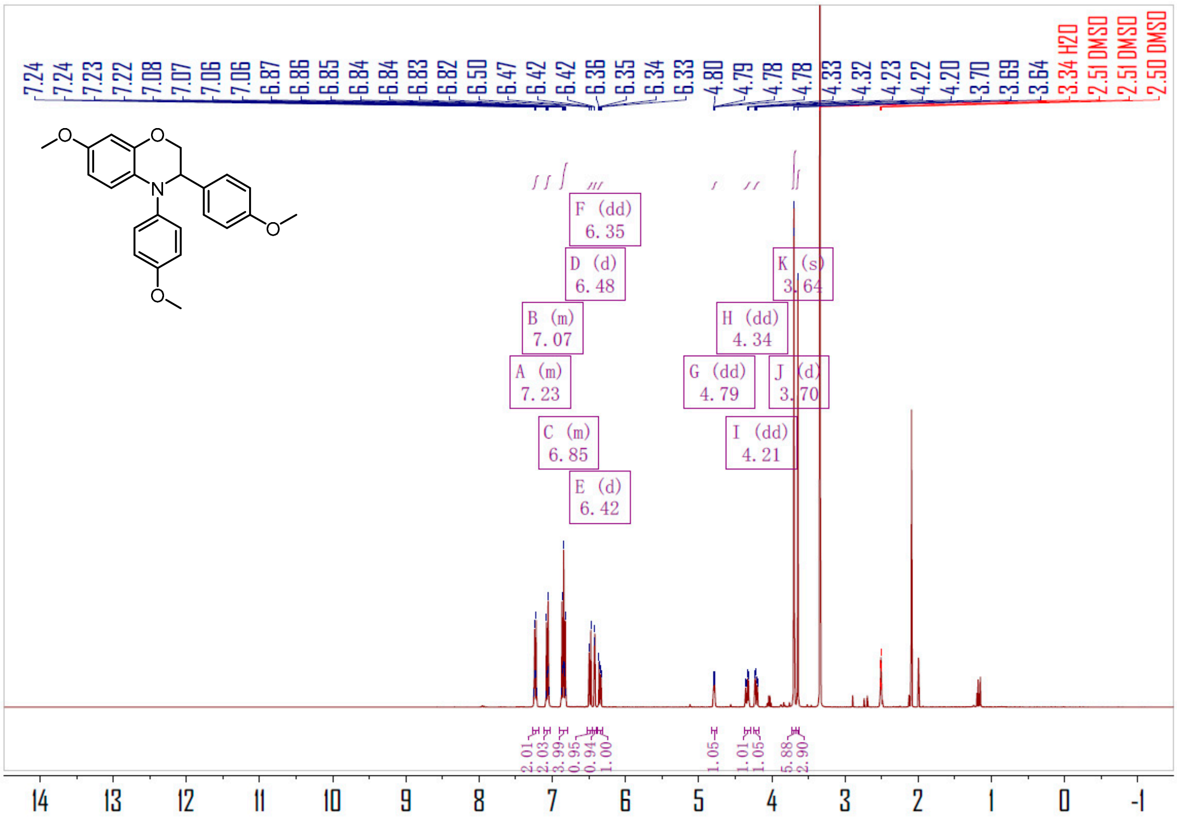

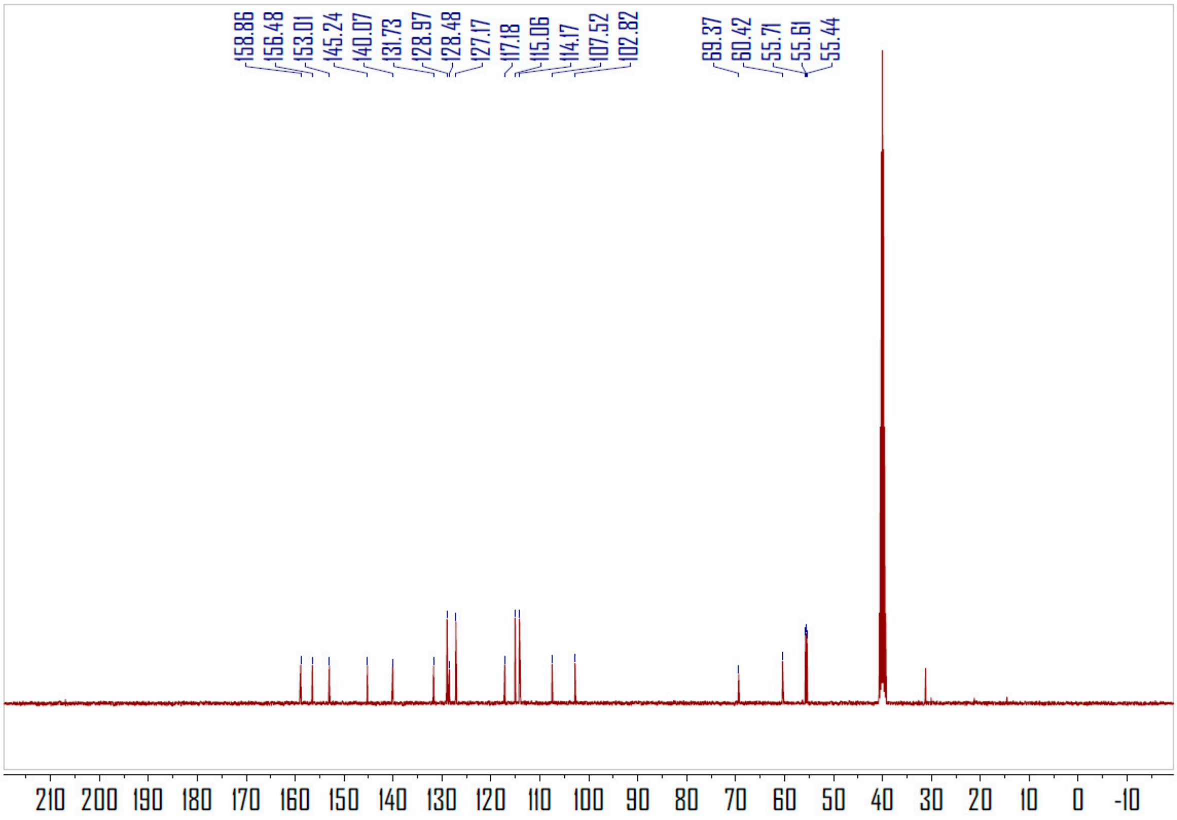

*7-methoxy-3-(4-methoxyphenyl)-4-(4-nitrophenyl)-3,4-dihydro-2H-benzo[b][1,4]oxazine (**11c**)*

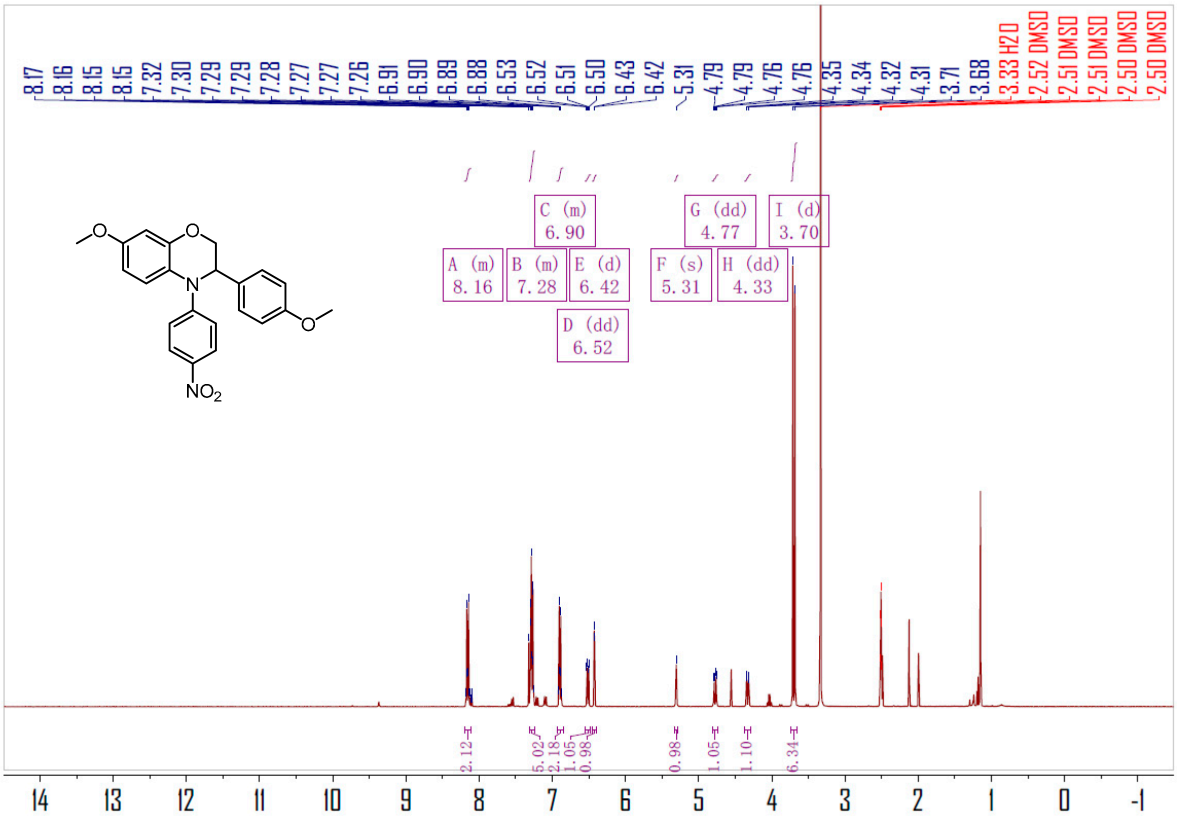

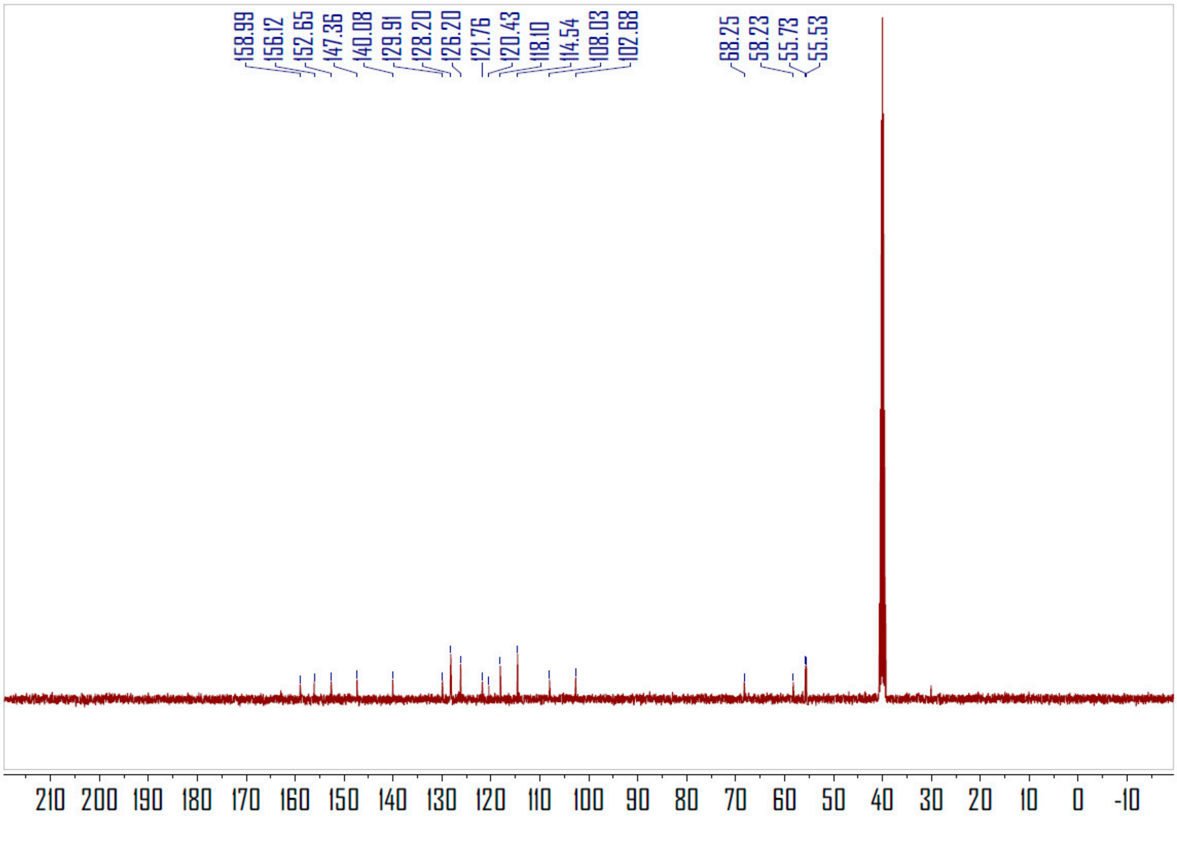

*3-(2,4-dimethylphenyl)-7-methoxy-4-(4-methoxyphenyl)-3,4-dihydro-2H-benzo[b][1,4]oxazine (**11d**)*

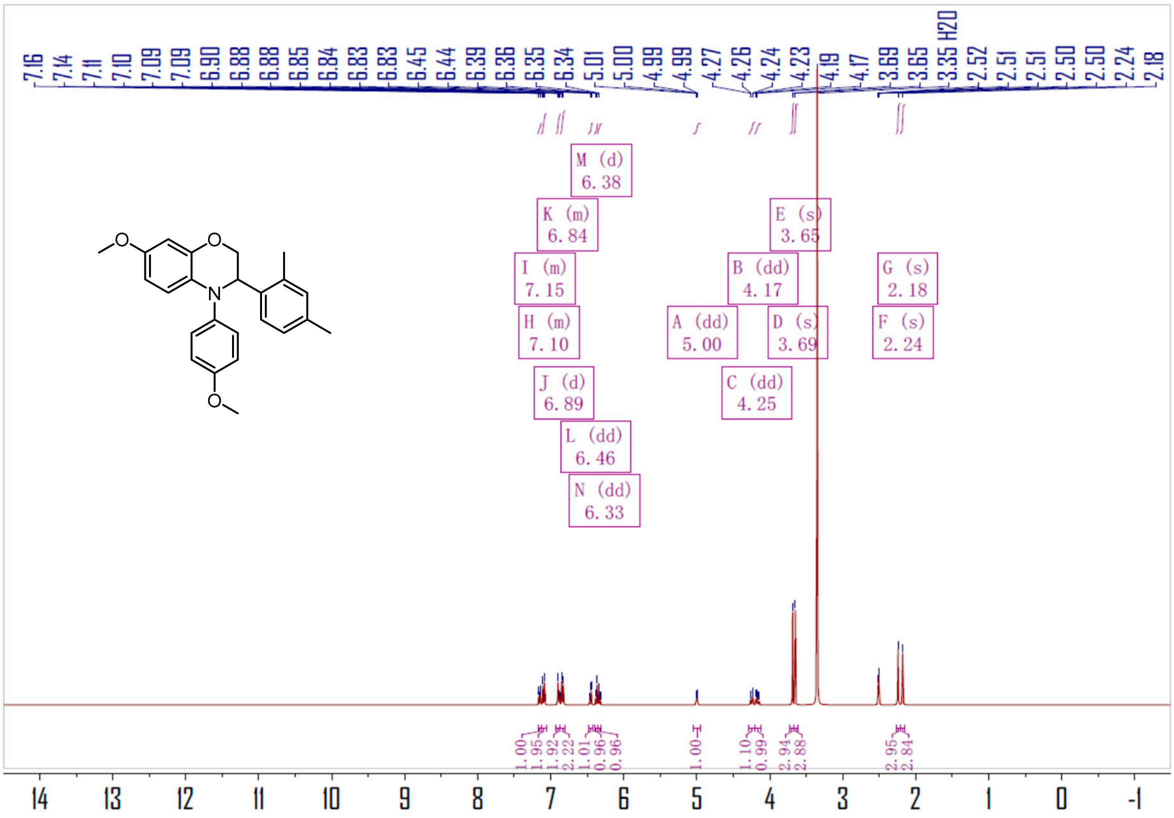

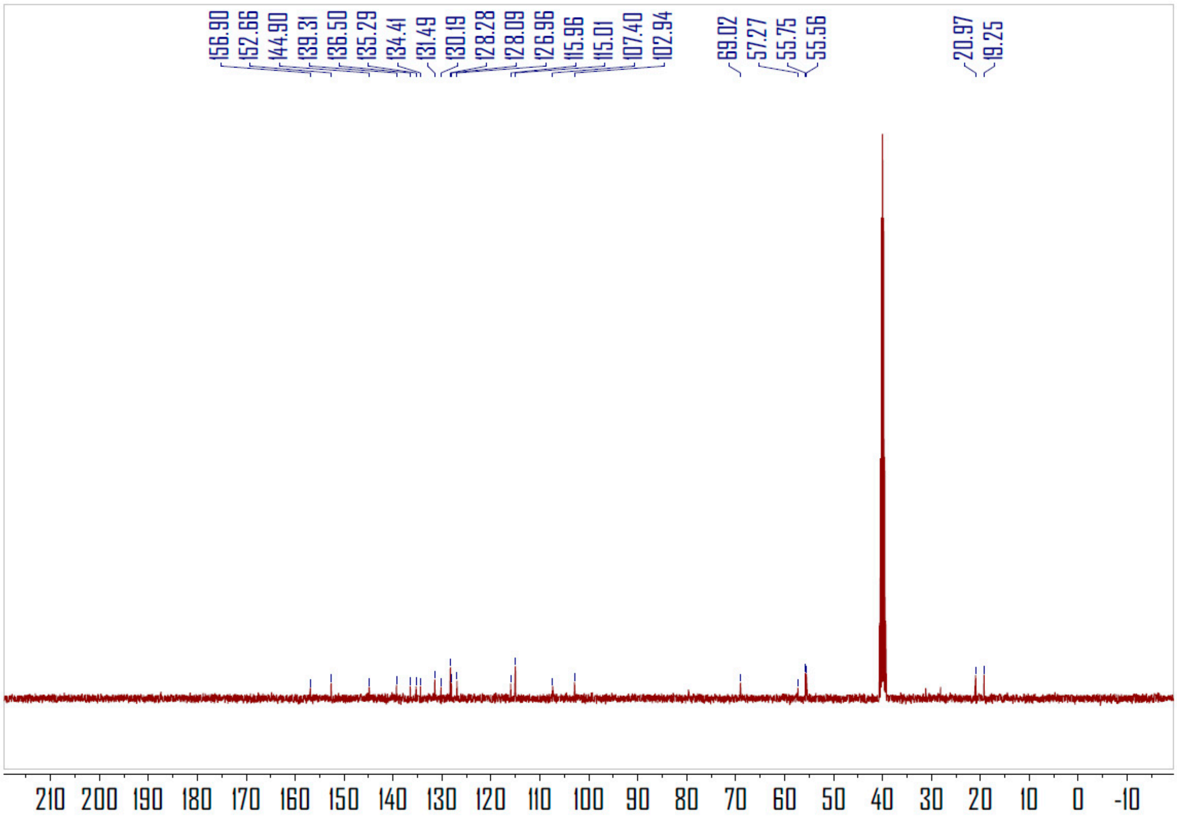

*3-(2,4-dimethylphenyl)-7-methoxy-4-(4-nitrophenyl)-3,4-dihydro-2H-benzo[b][1,4]oxazine (**11e**)*

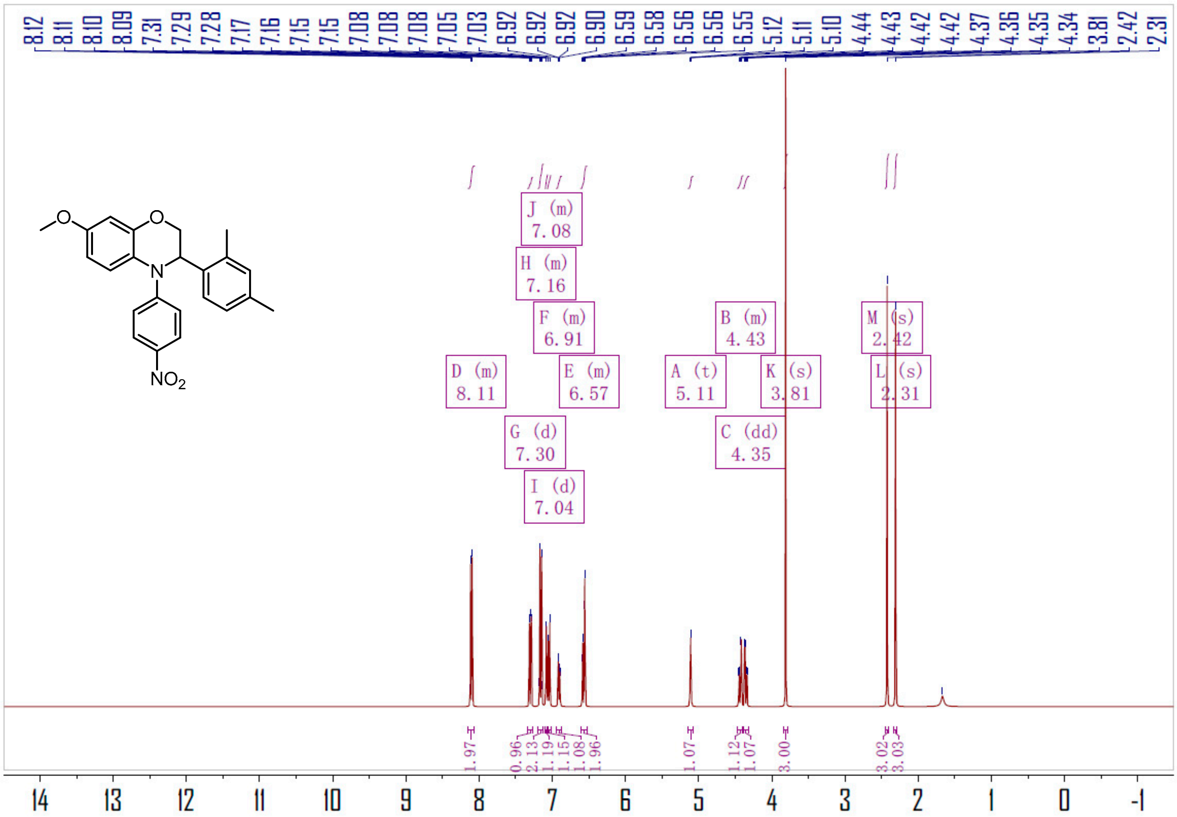

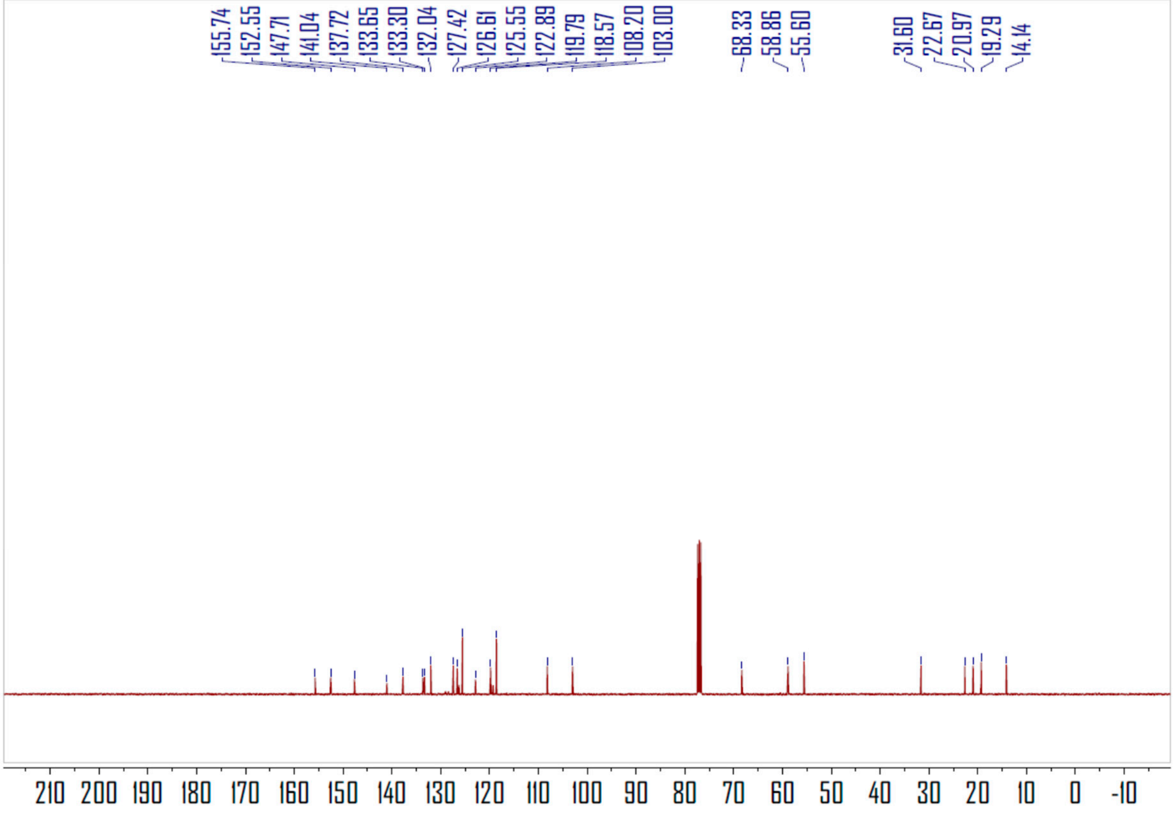

*4-(2,4-dimethoxyphenyl)-7-methoxy-3-(4-methoxyphenyl)-4H-benzo[b][1,4]oxazine (**12**)*

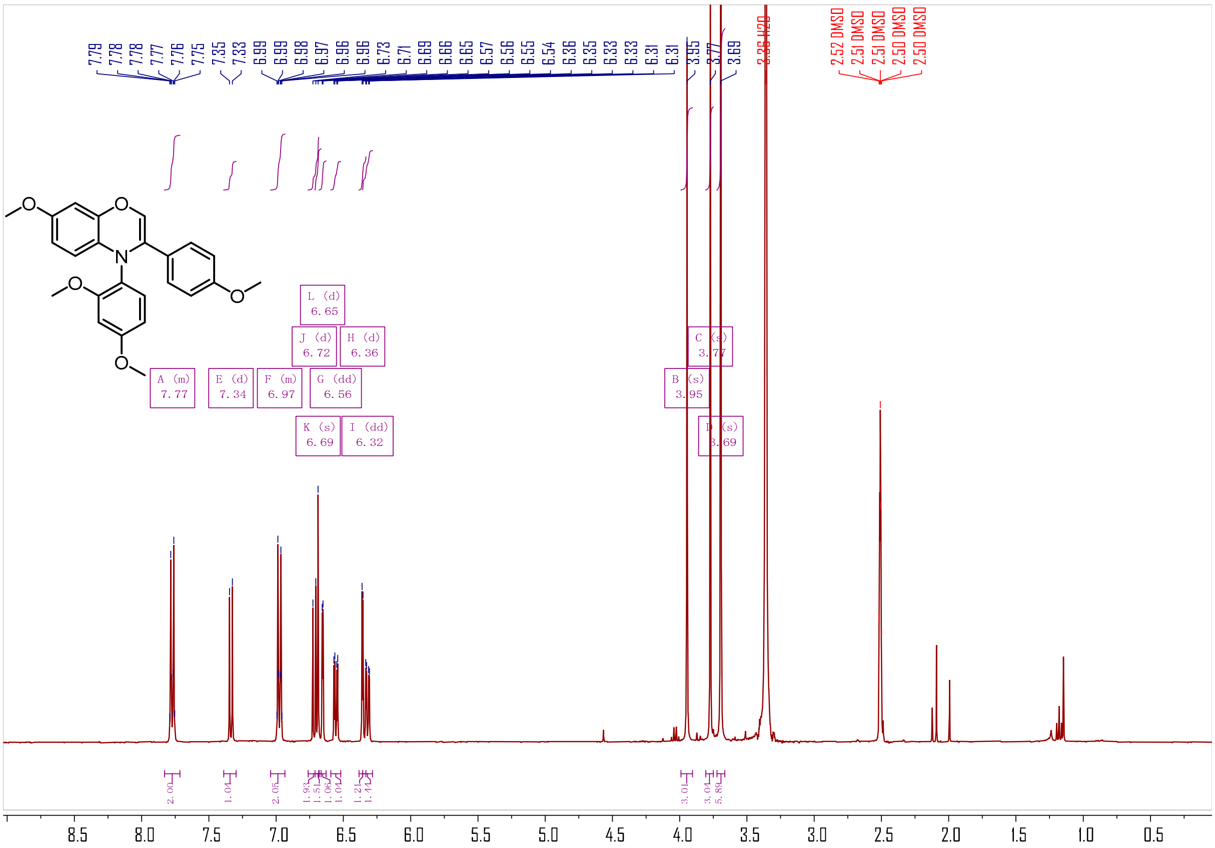

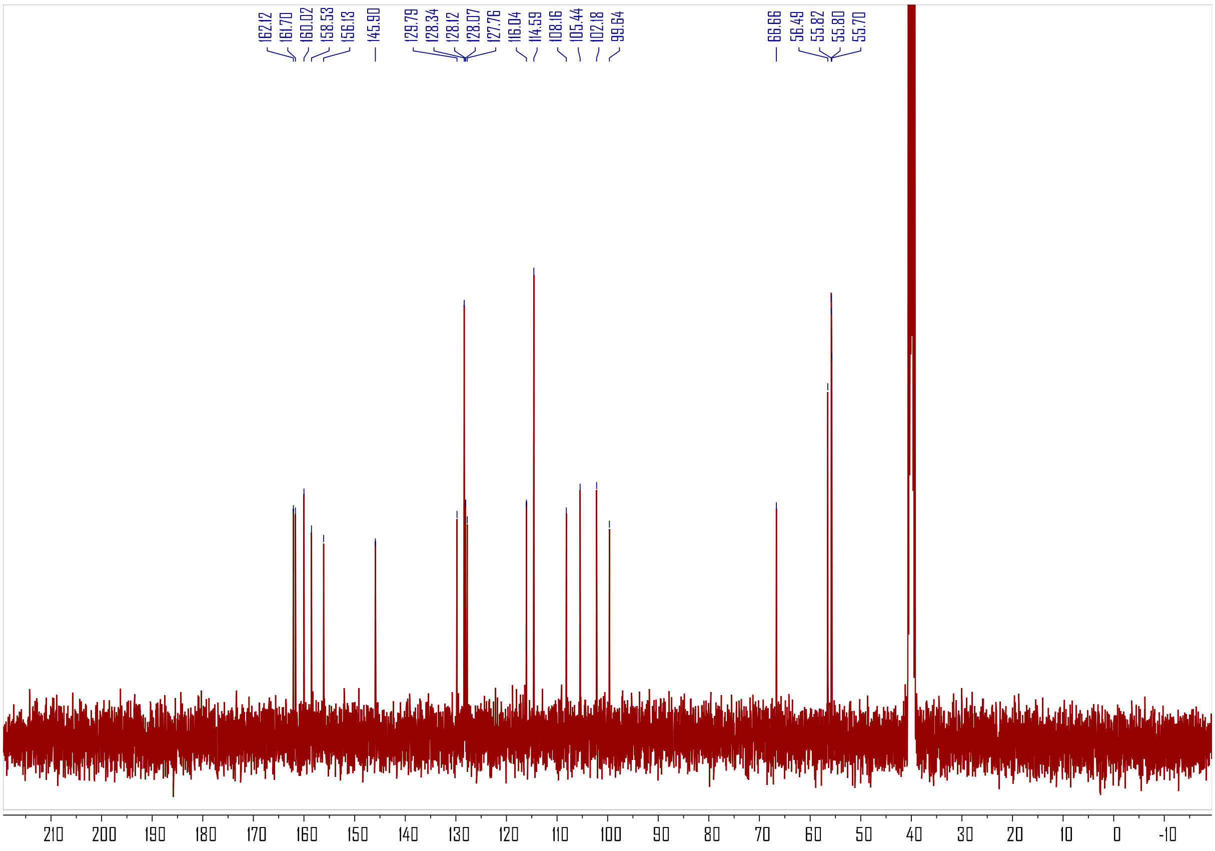

3-(2,4-dimethylphenyl)-7-methoxy-4-(quinolin-3-yl)-3,4-dihydro-2H-benzo[b][1,4]oxazine (***15a***)

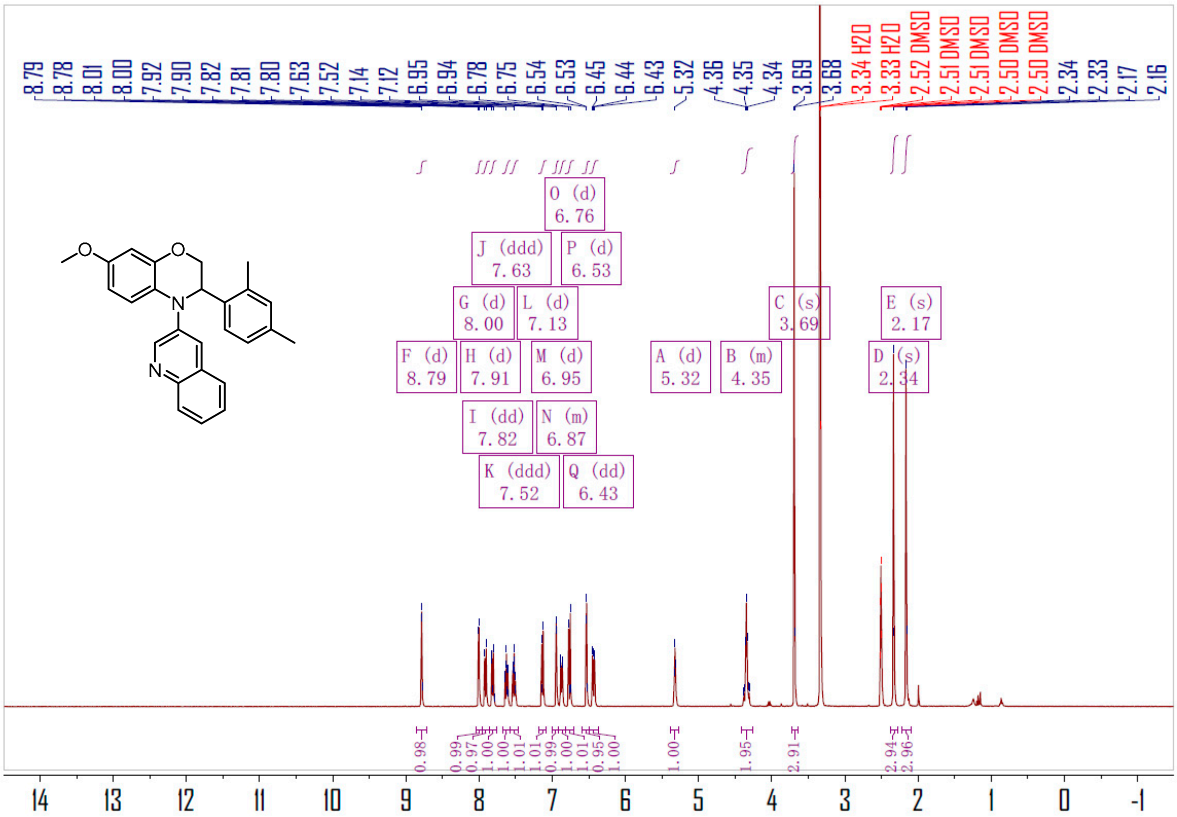

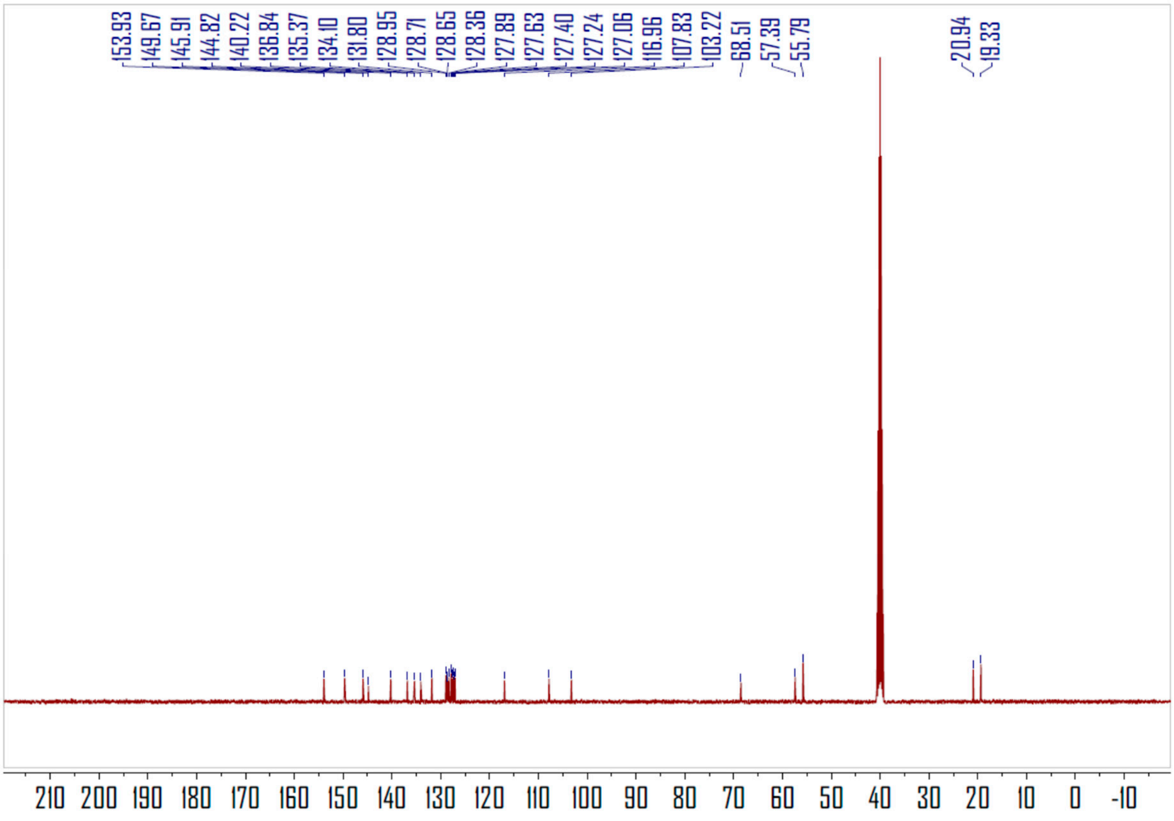

*3-(4-fluorophenyl)-7-methoxy-4-(4-methoxyphenyl)-3,4-dihydro-2H-benzo[b][1,4]oxazine (**11f**)*

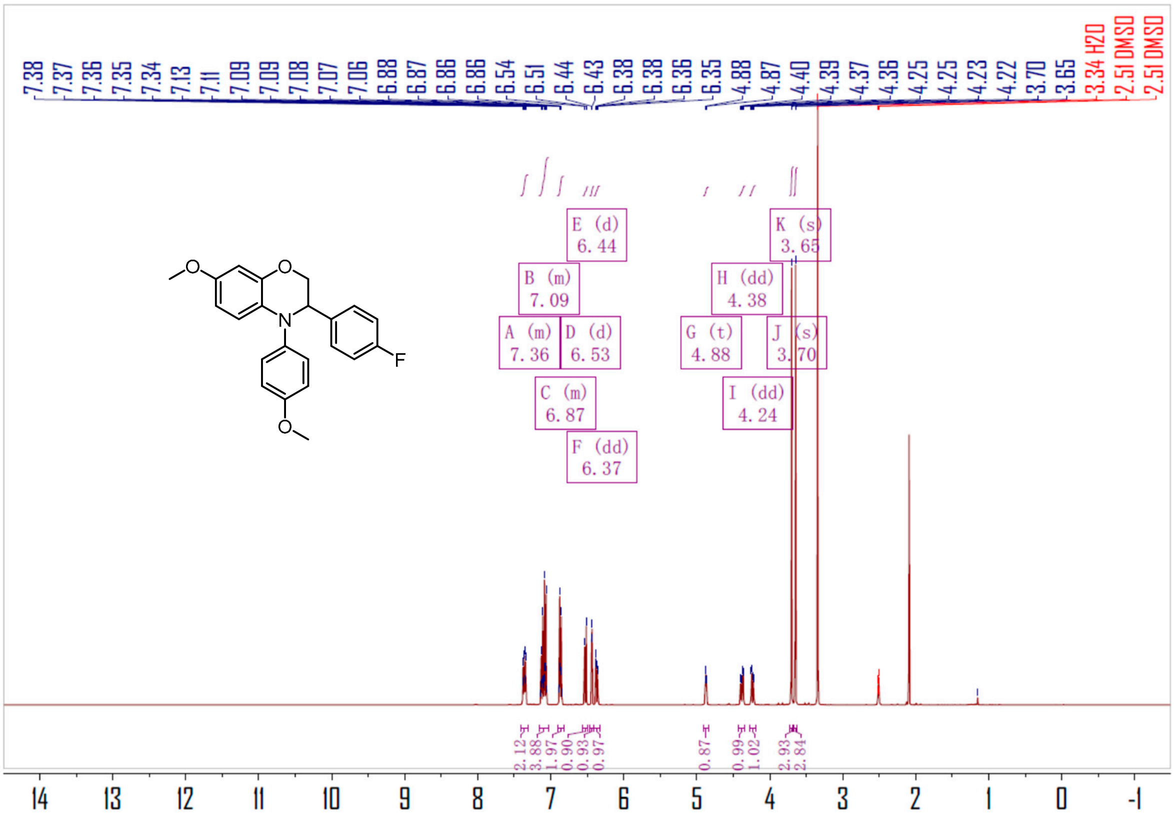

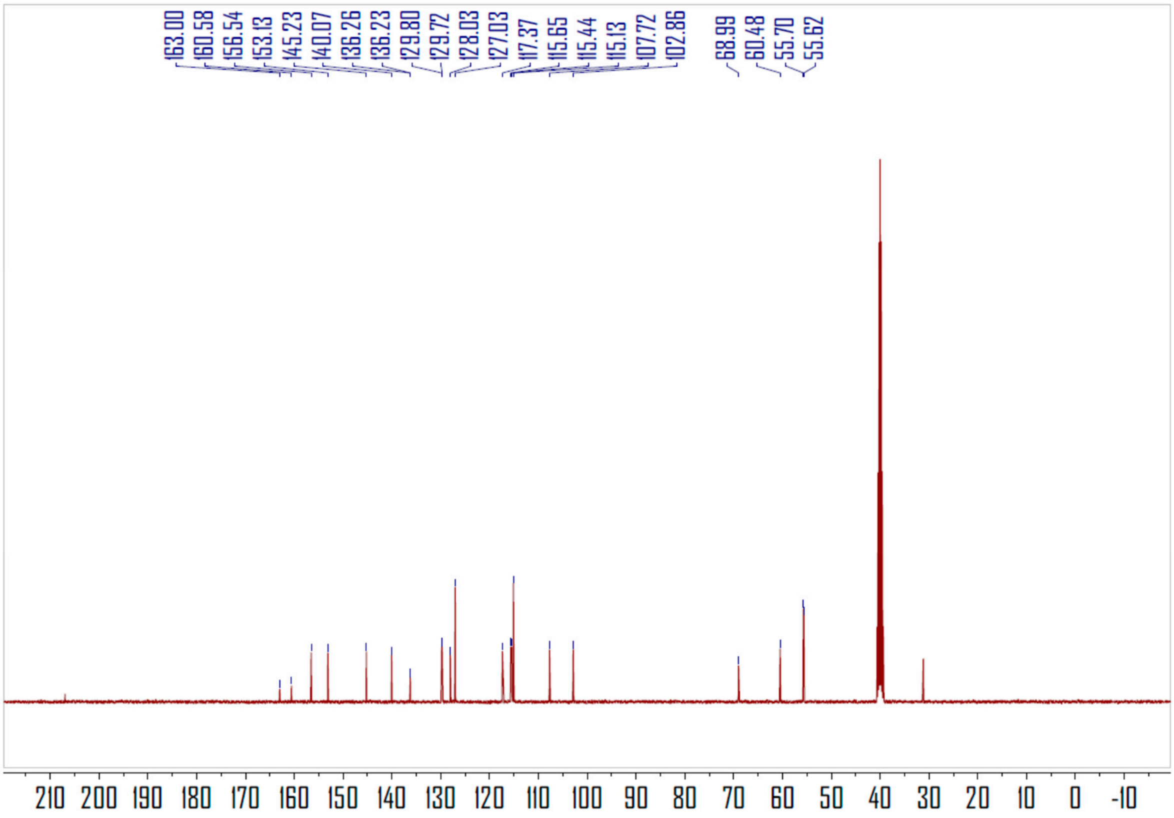

*3-(4-fluorophenyl)-7-methoxy-4-(4-nitrophenyl)-3,4-dihydro-2H-benzo[b][1,4]oxazine (**11g**)*

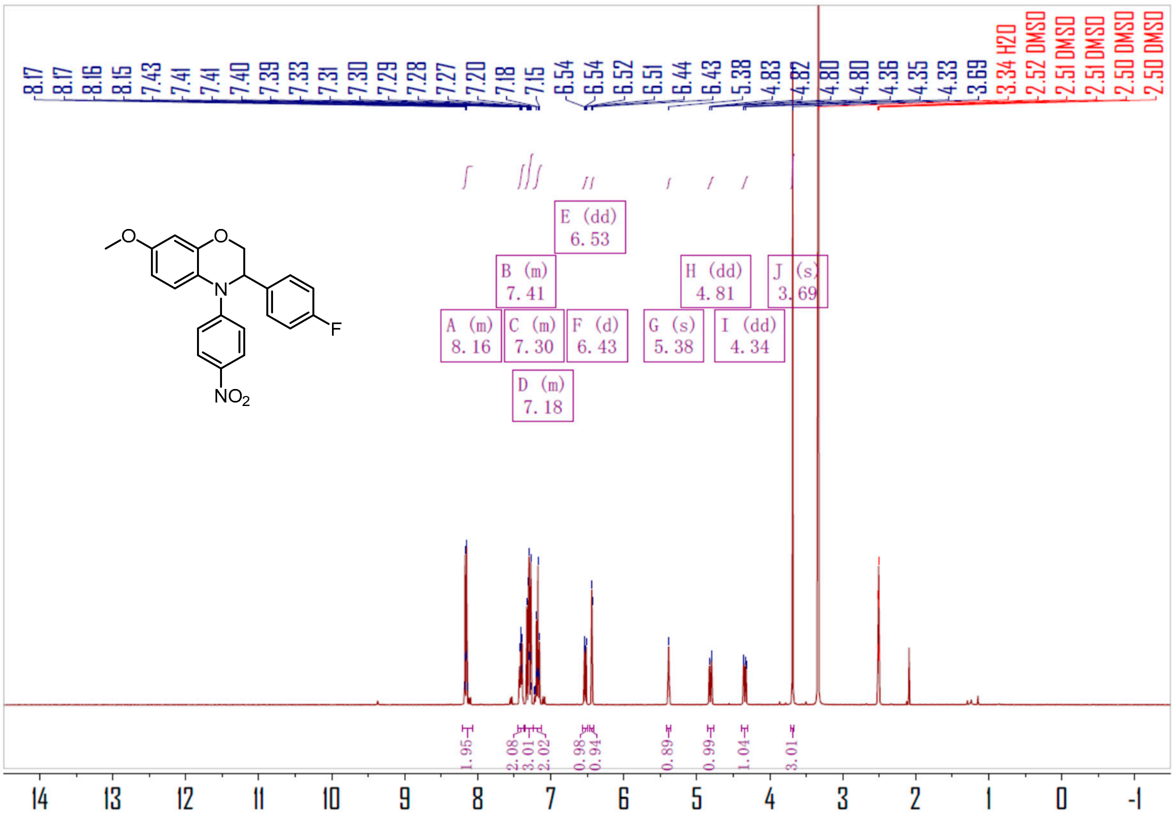

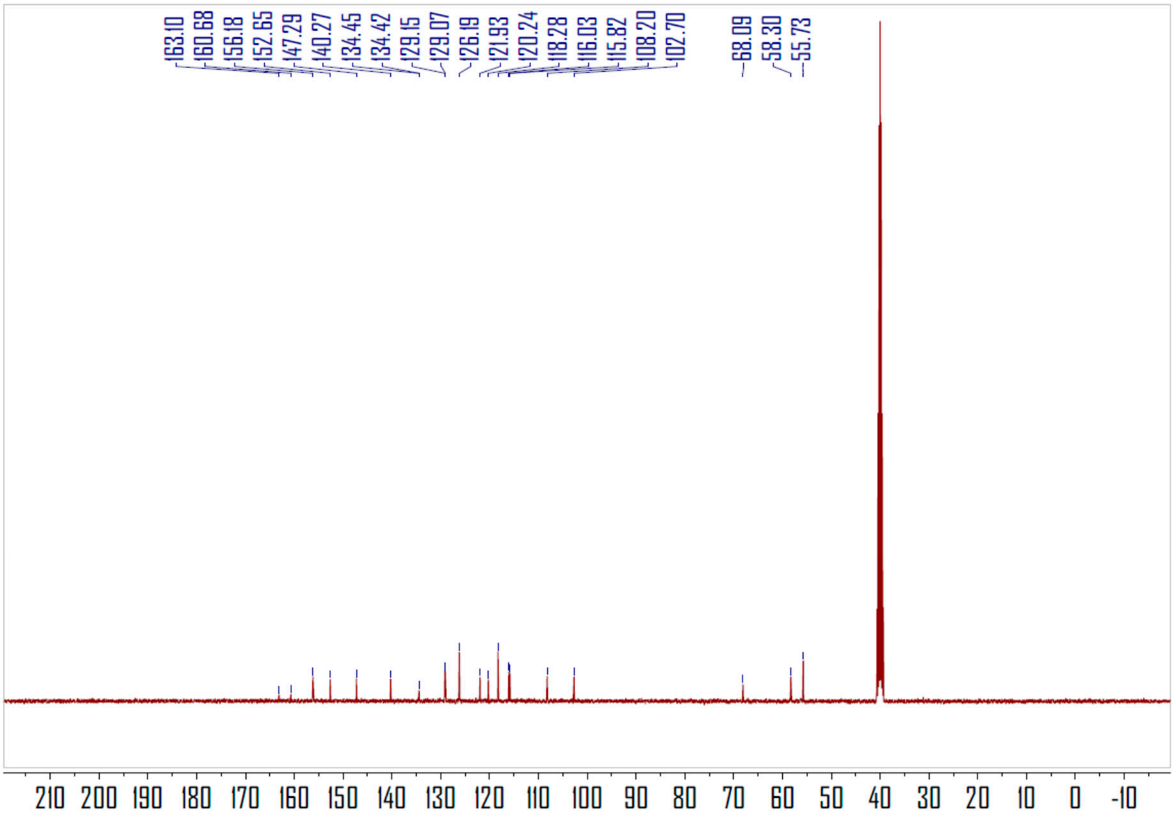

*3-(4-fluorophenyl)-7-methoxy-4-(quinolin-3-yl)-3,4-dihydro-2H-benzo[b][1,4]oxazine (**15b**)*

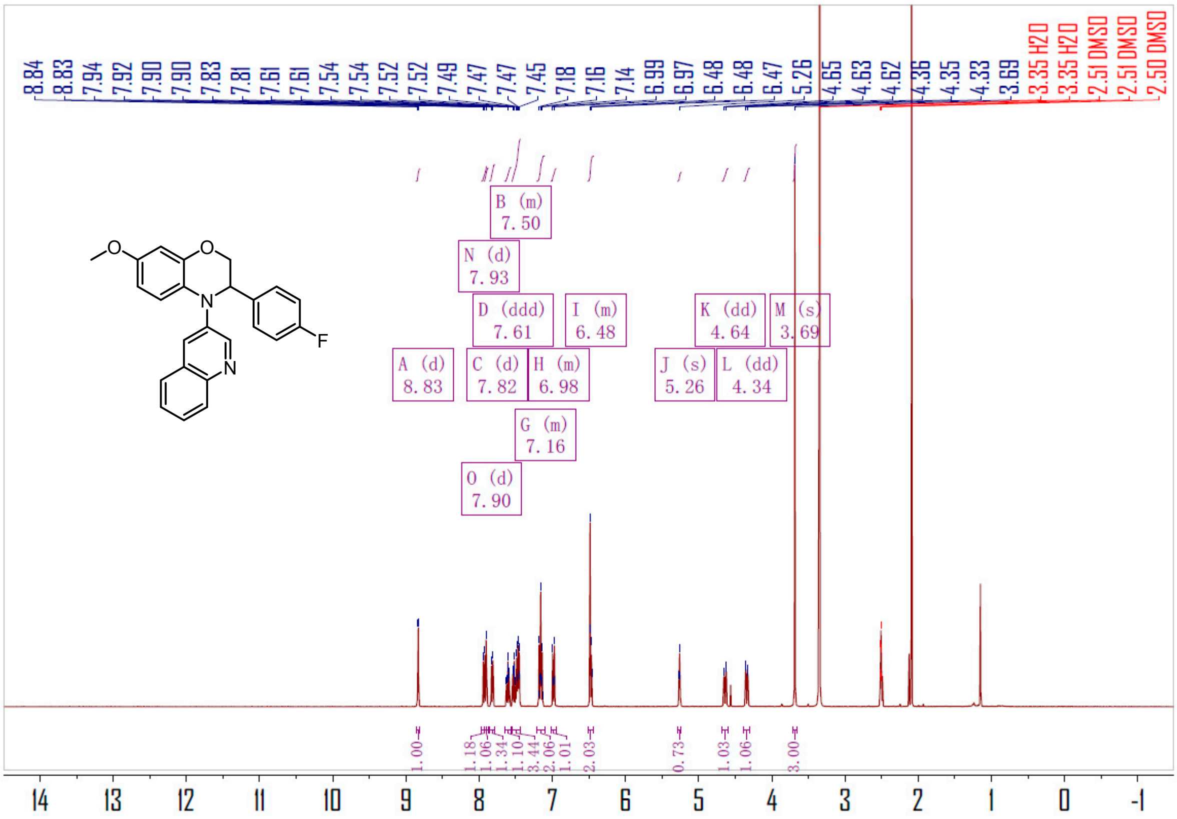

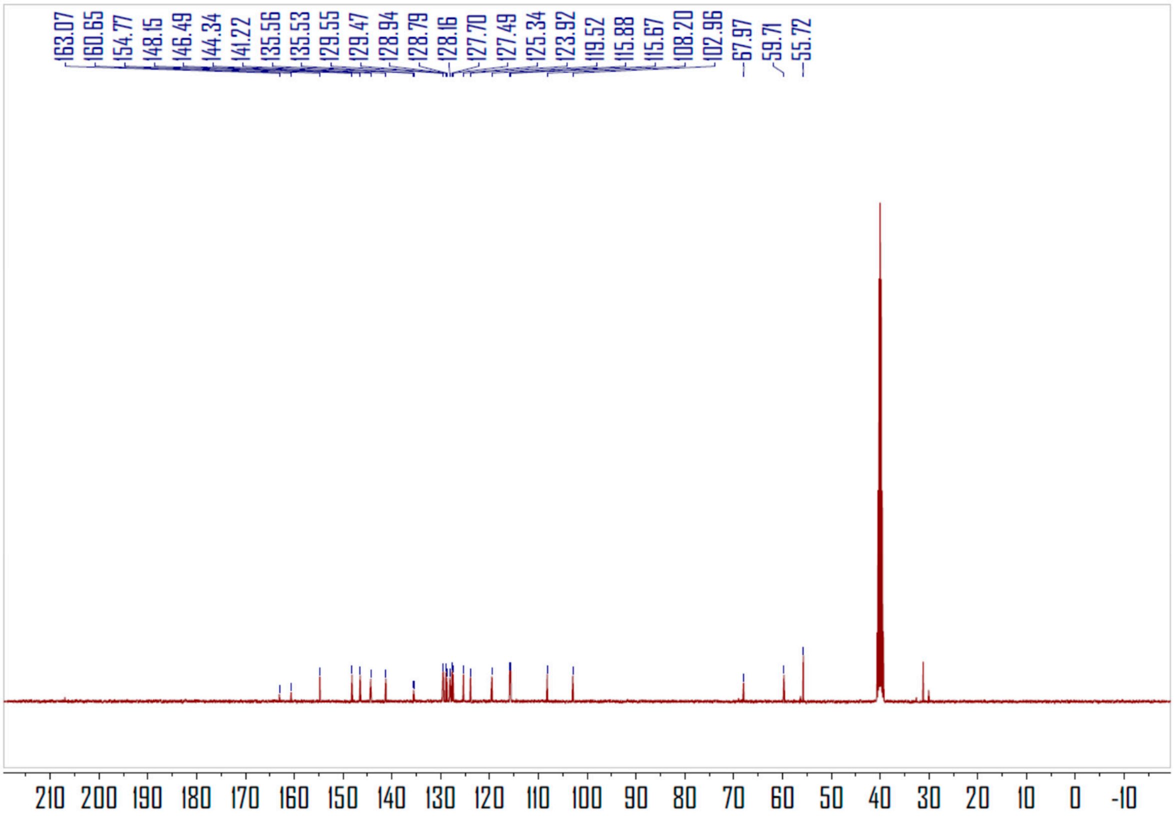

*4-(3-phenyl-2,3-dihydro-4H-benzo[b][1,4]oxazin-4-yl)aniline (**13a**)*

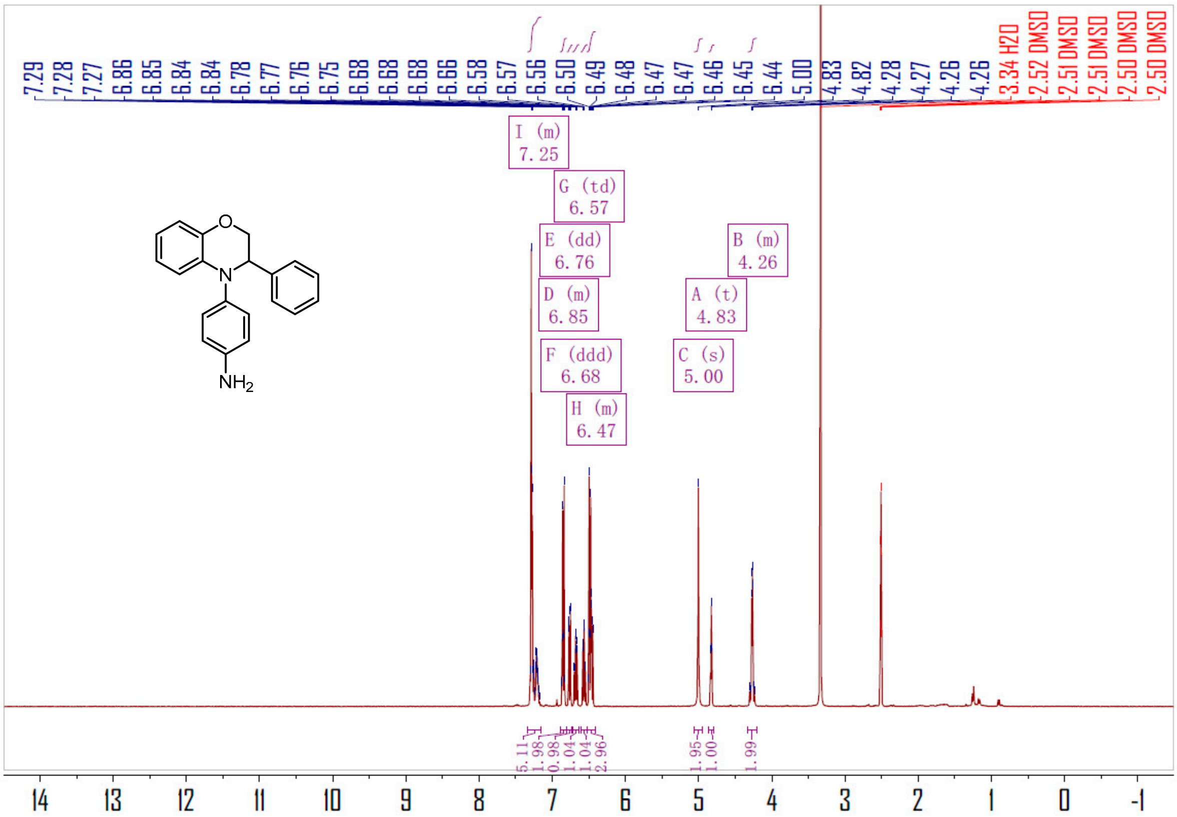

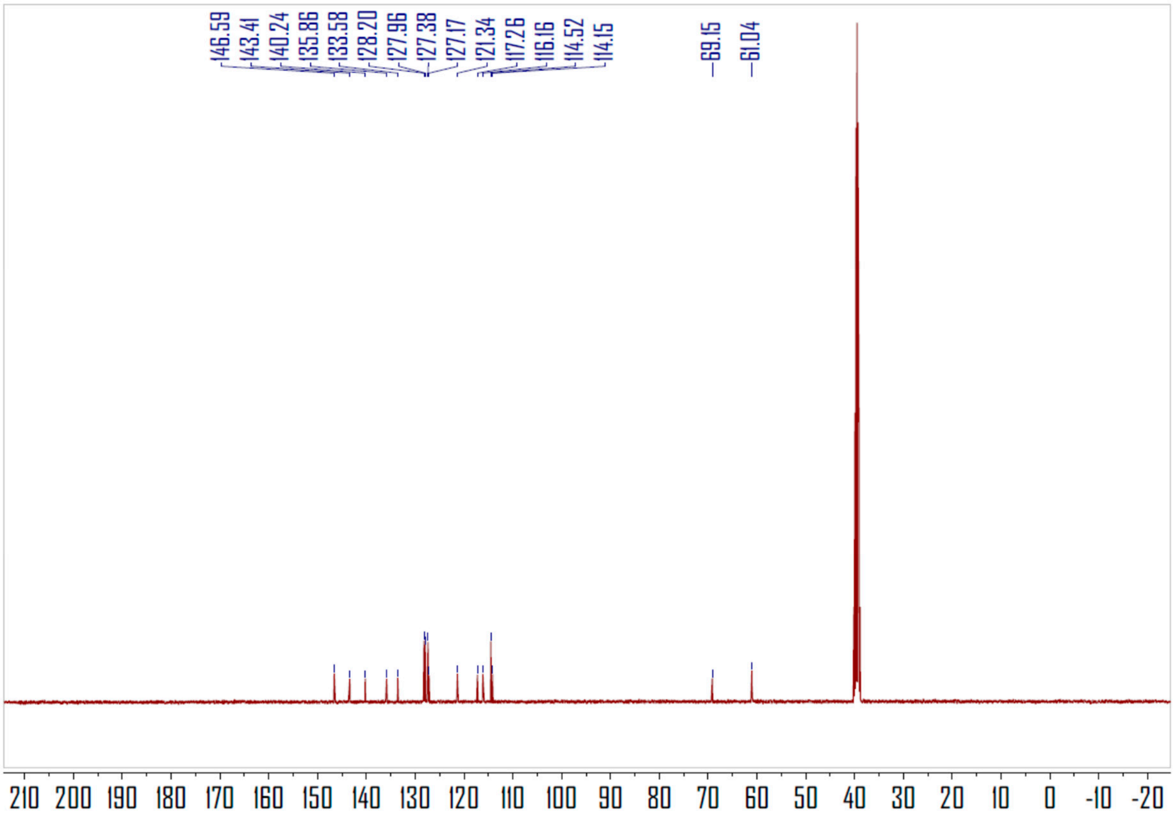

*4-(3-(4-methoxyphenyl)-2,3-dihydro-4H-benzo[b][1,4]oxazin-4-yl)aniline (**13b**)*

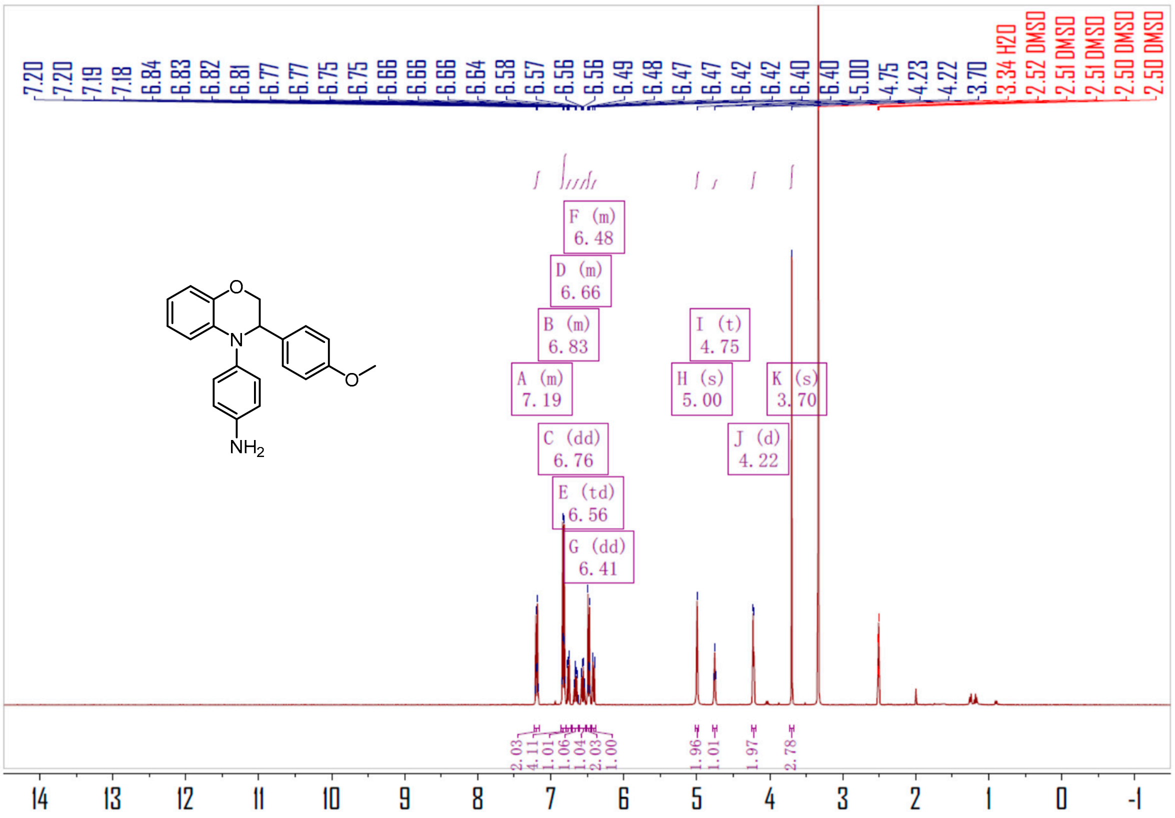

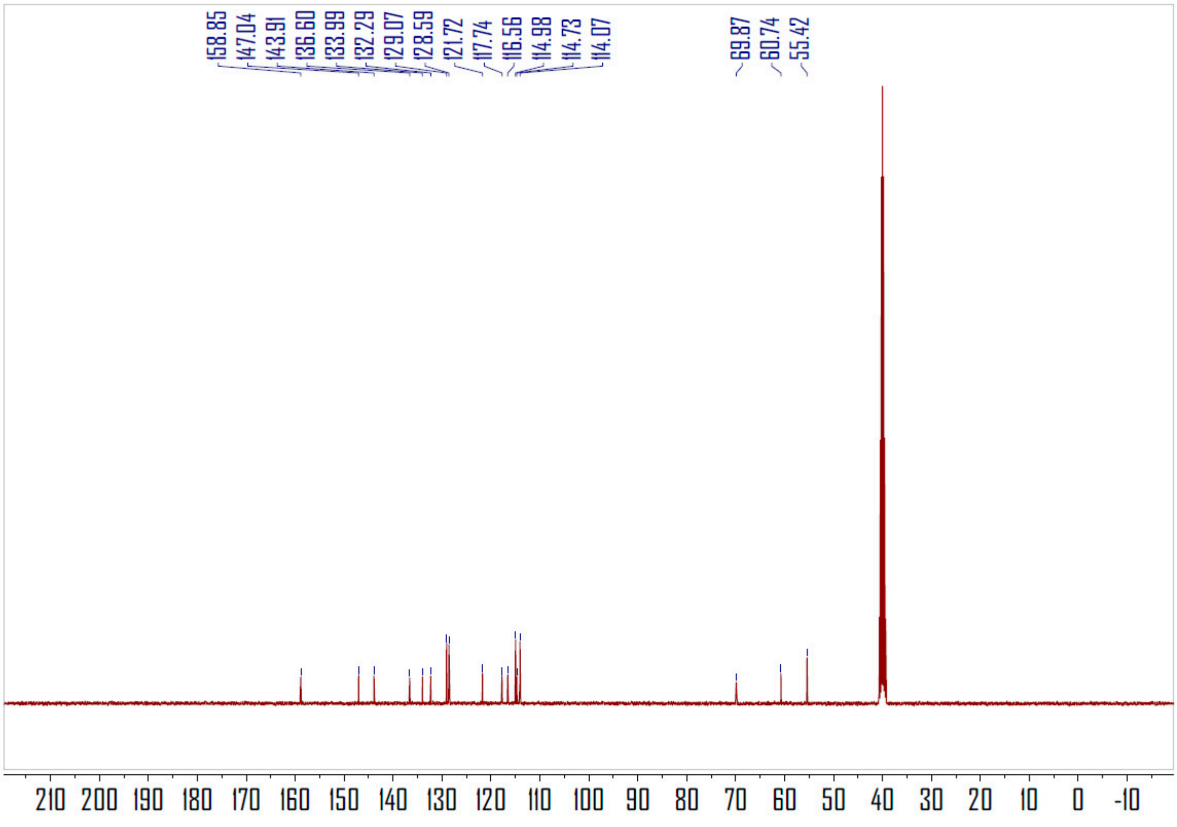

4-(7-methoxy-3-(4-methoxyphenyl)-2,3-dihydro-4H-benzo[b][1,4]oxazin-4-yl)aniline (**13c**)

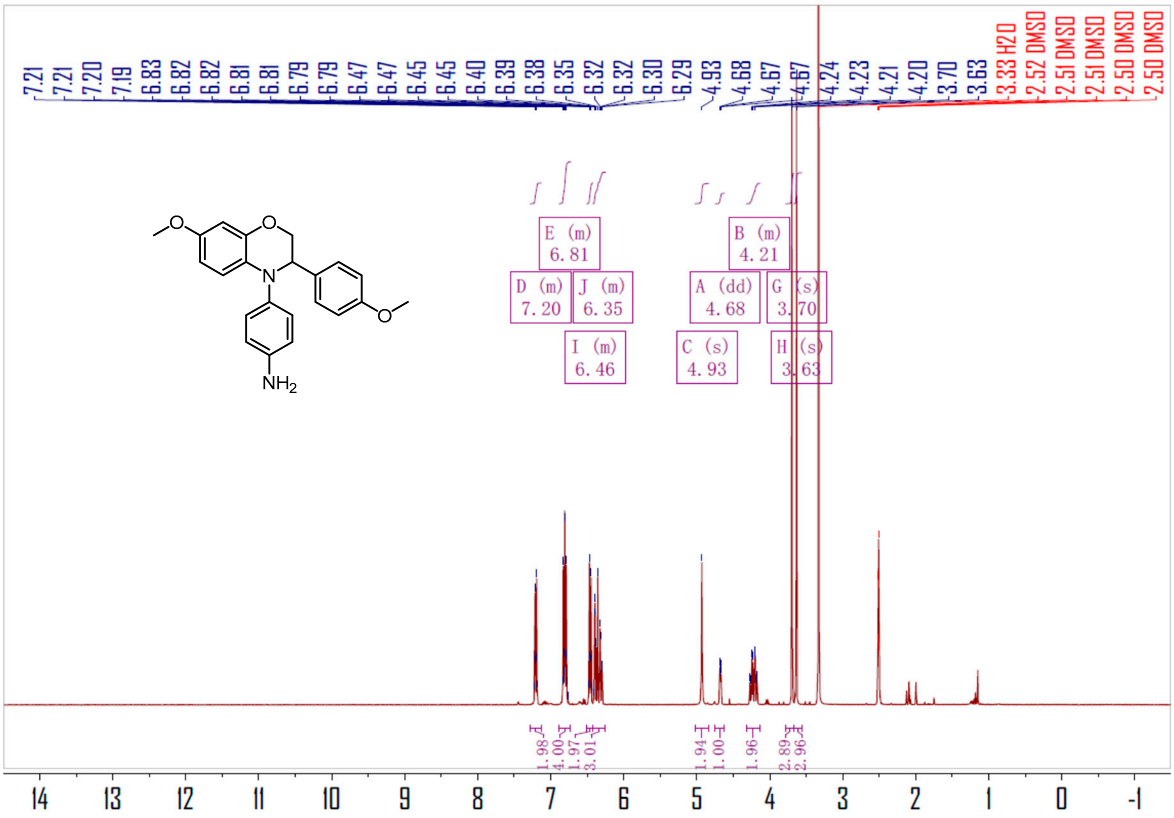

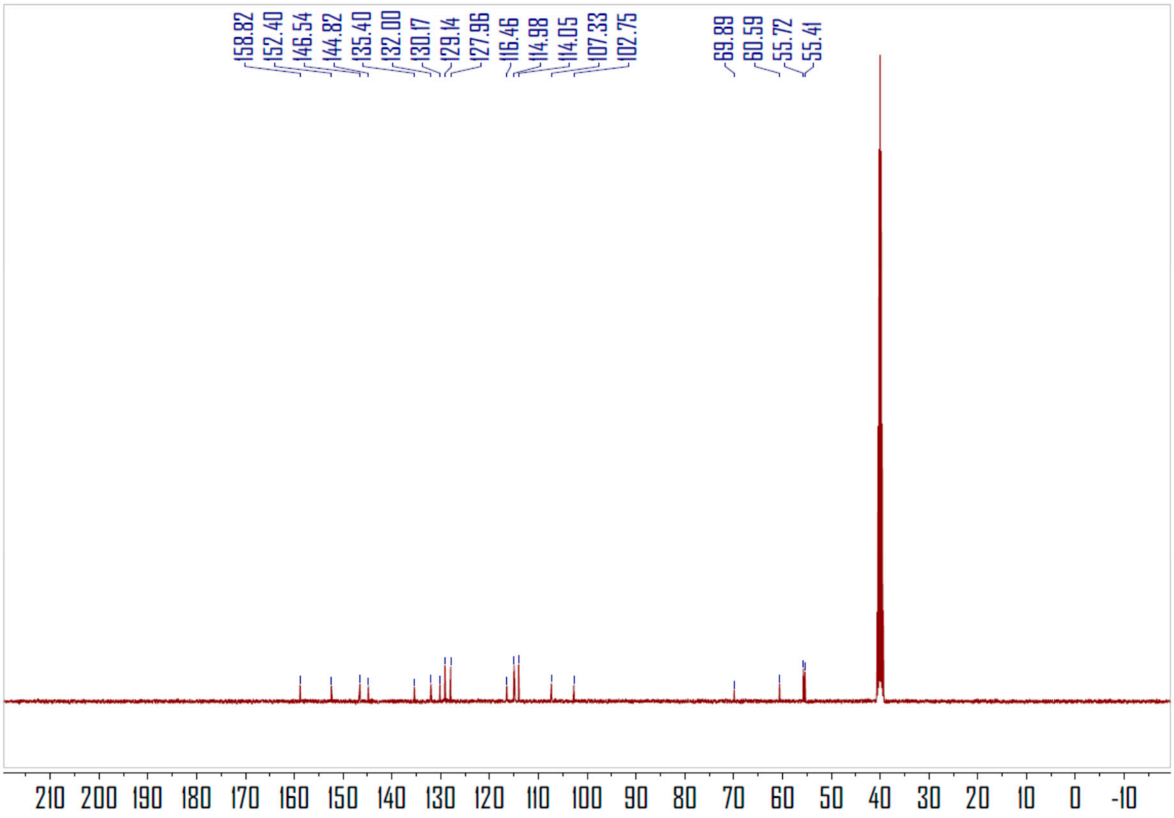

*4-(3-(2,4-dimethylphenyl)-7-methoxy-2,3-dihydro-4H-benzo[b][1,4]oxazin-4-yl)aniline (**13d**)*

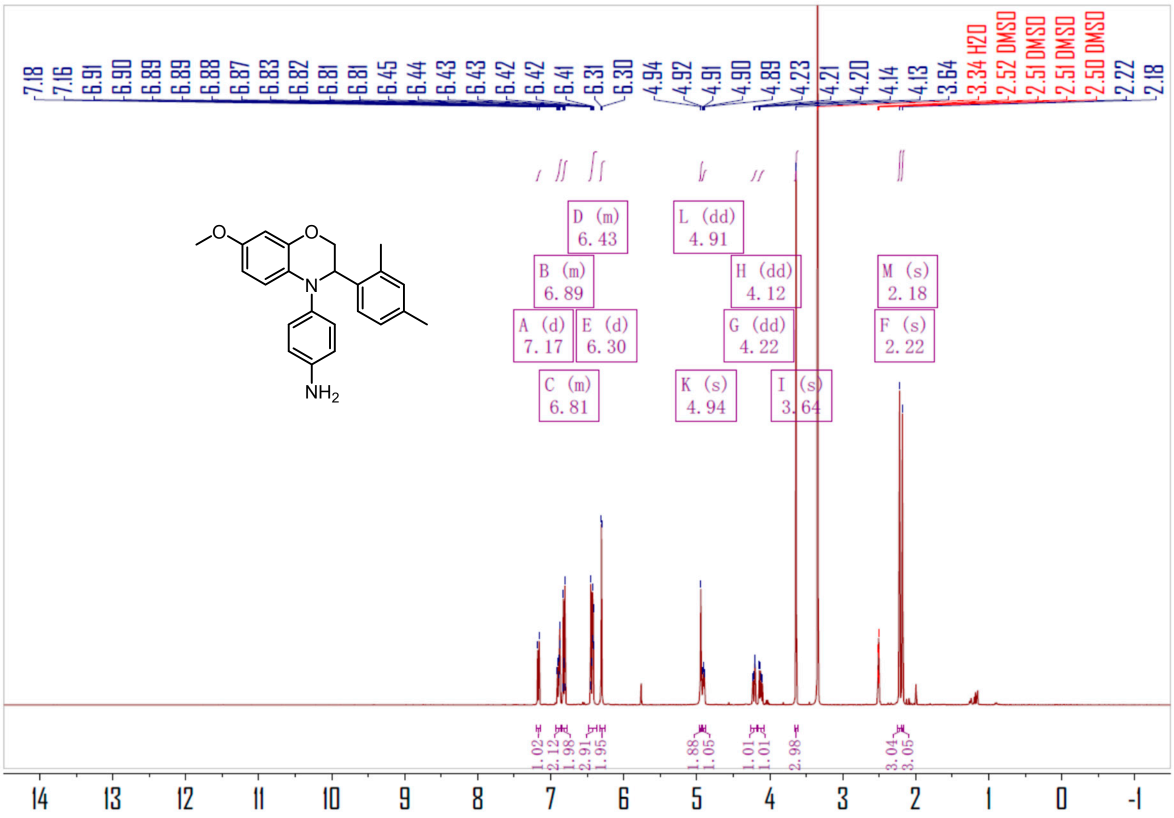

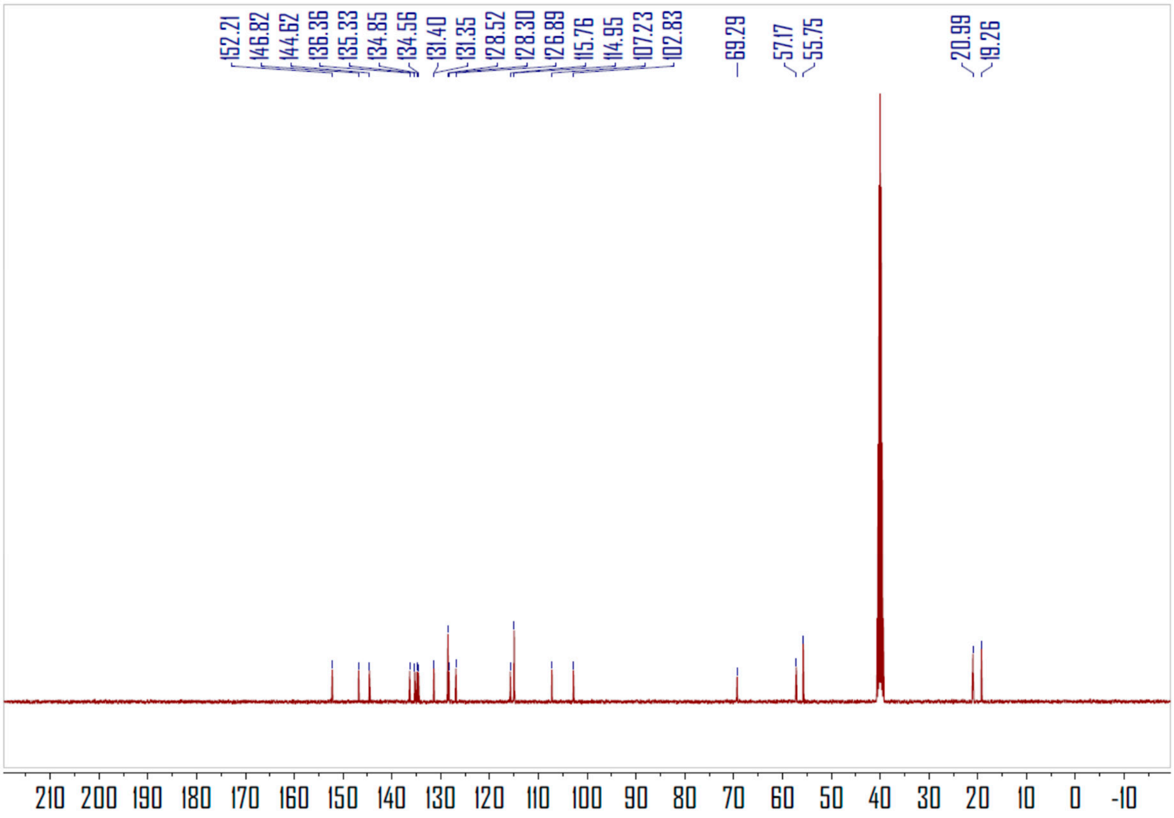

*4-(3-(4-fluorophenyl)-7-methoxy-2,3-dihydro-4H-benzo[b][1,4]oxazin-4-yl)aniline (**13e**)*

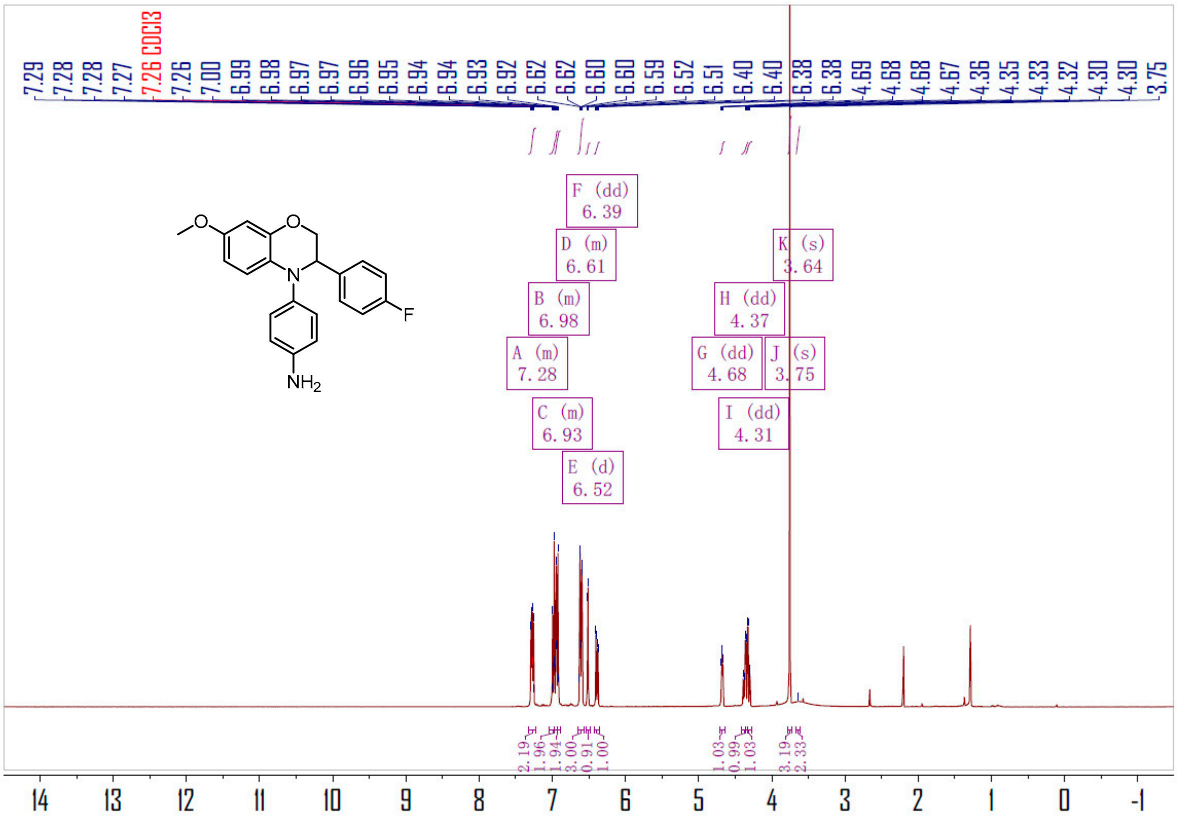

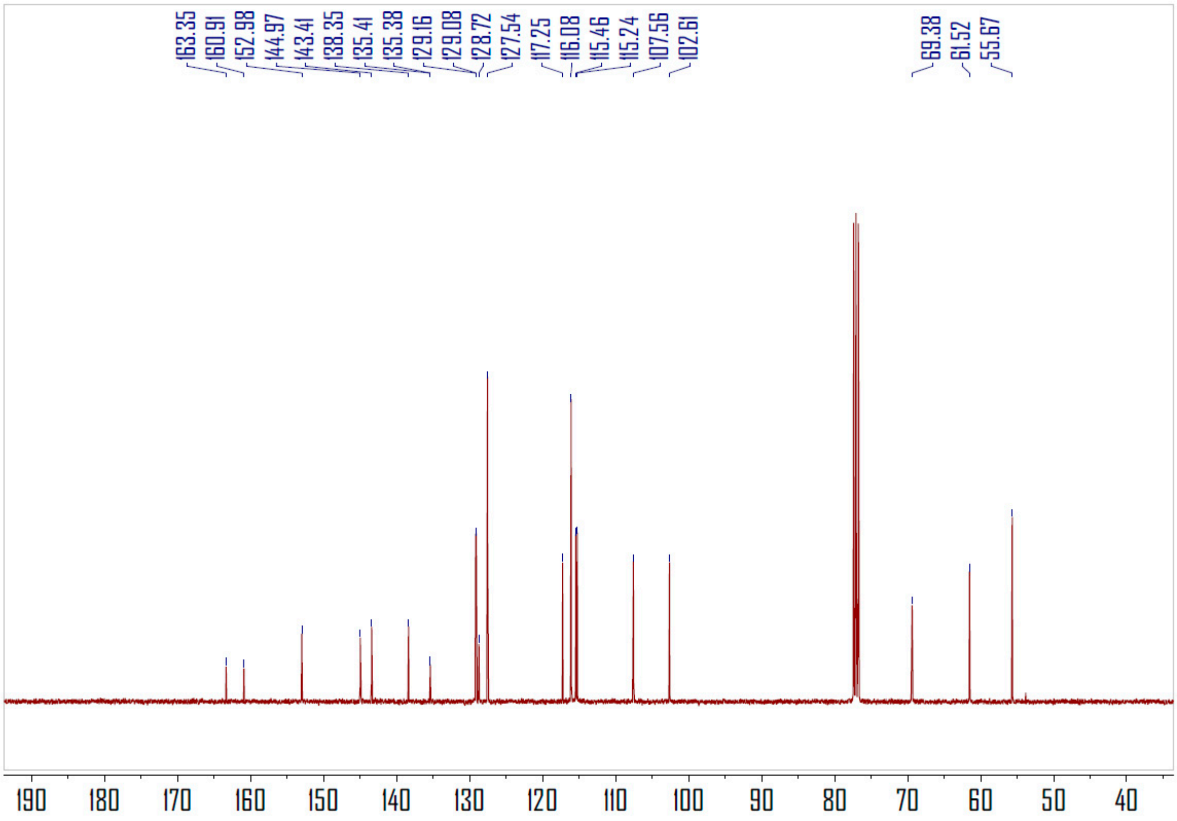

*4-(4-phenyl-3,4-dihydro-2H-benzo[b][1,4]oxazin-3-yl)phenol (**14a**)*

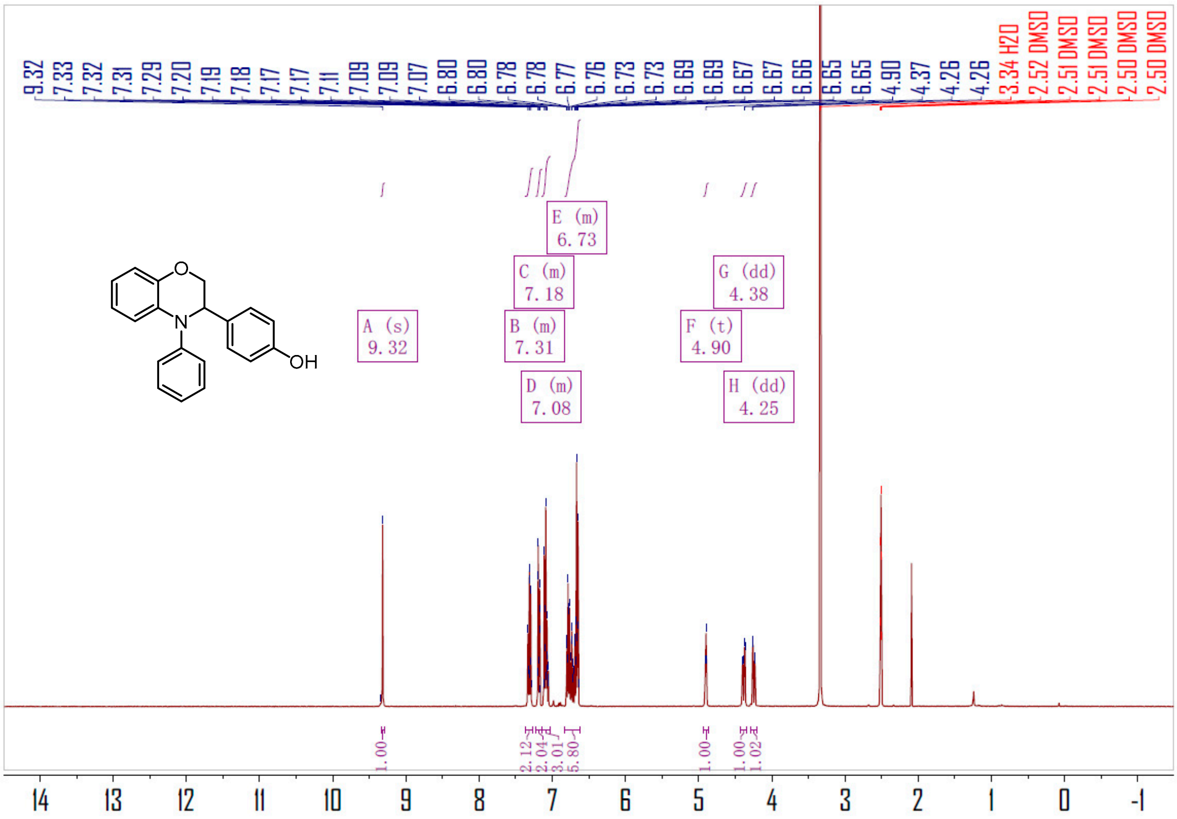

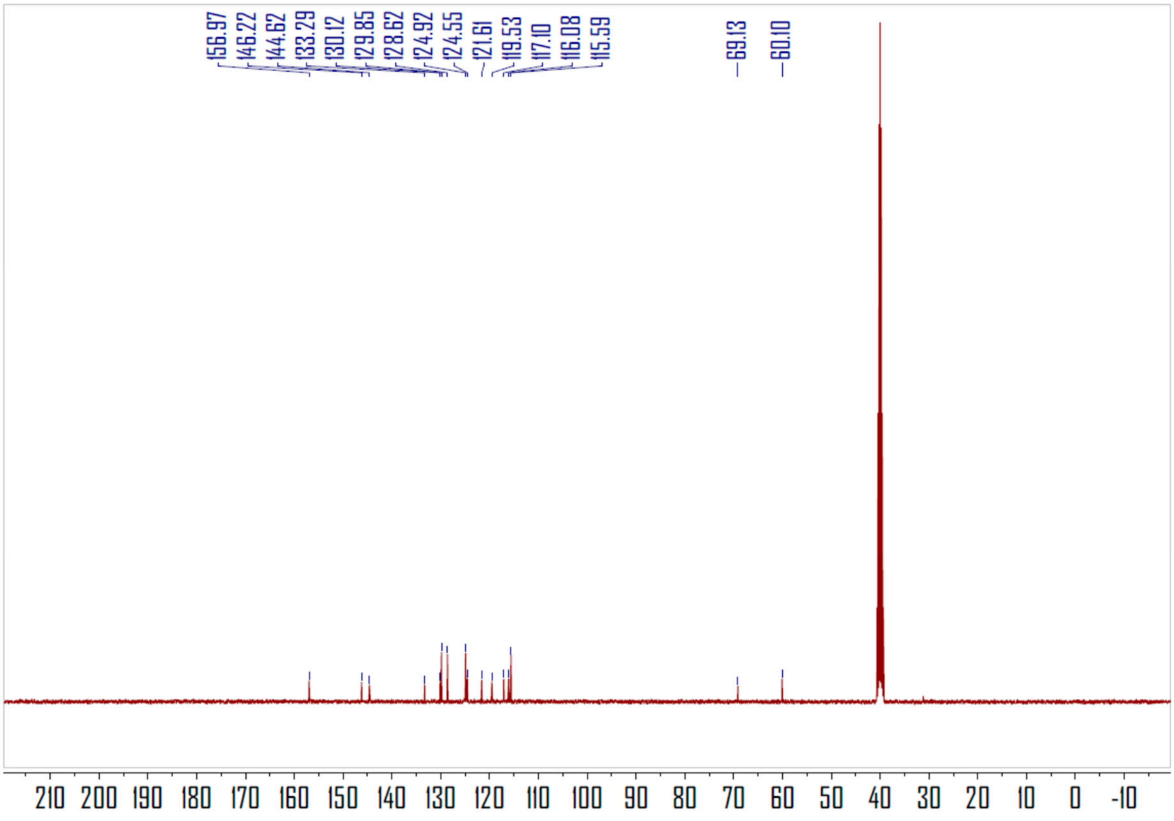

*4-(4-(4-aminophenyl)-3,4-dihydro-2H-benzo[b][1,4]oxazin-3-yl)phenol (**14b**)*

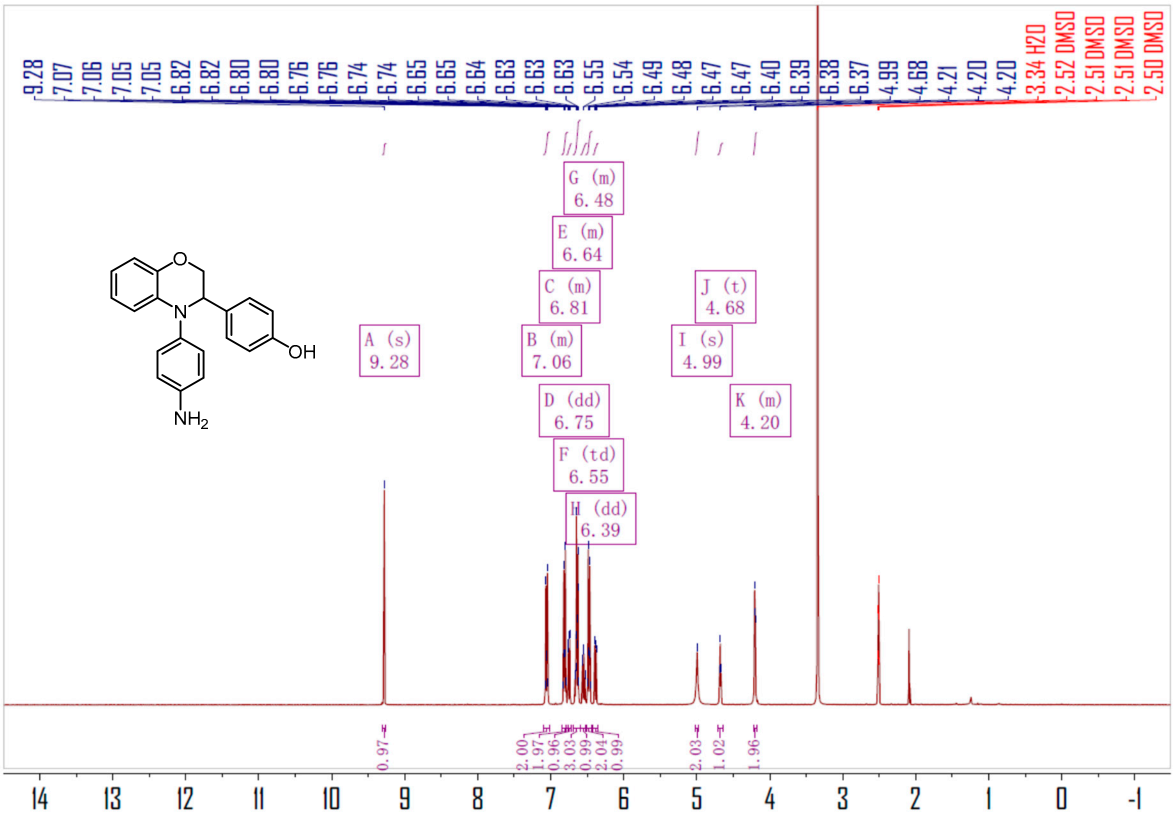

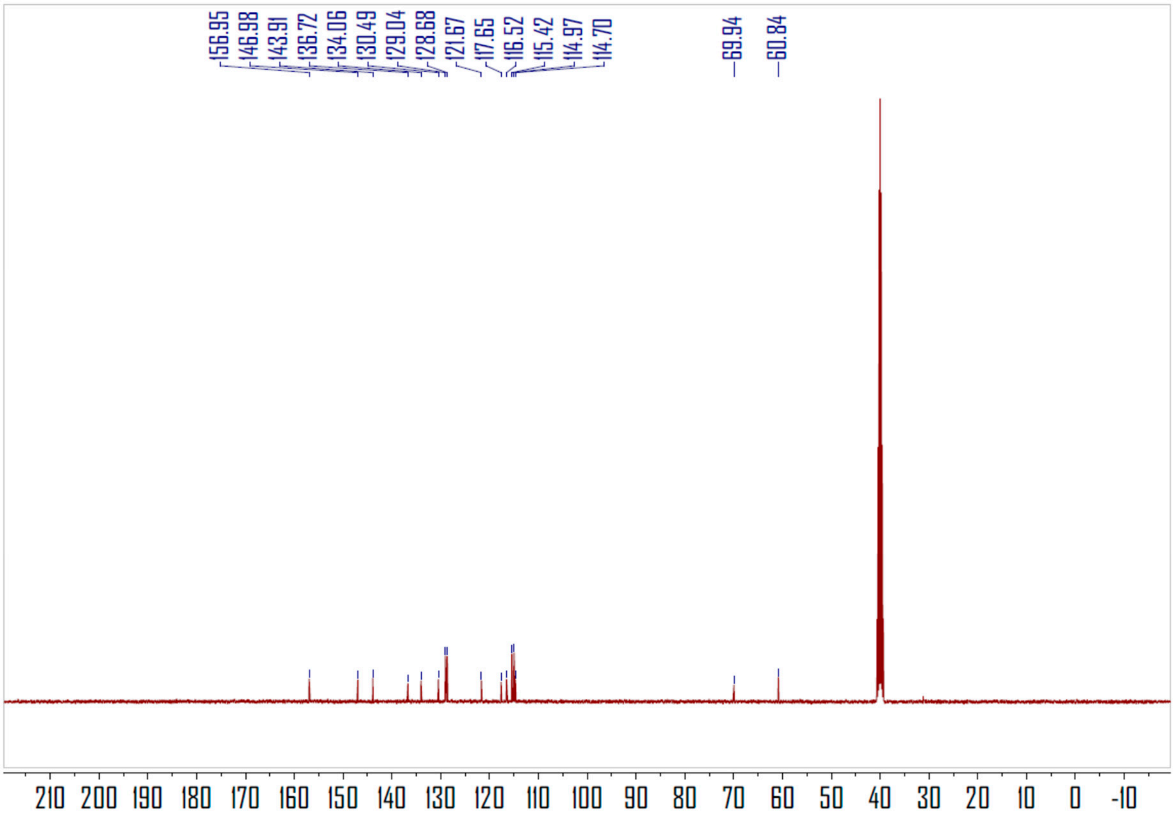

*3-(4-hydroxyphenyl)-4-phenyl-3,4-dihydro-2H-benzo[b][1,4]oxazin-7-ol (**14c**)*

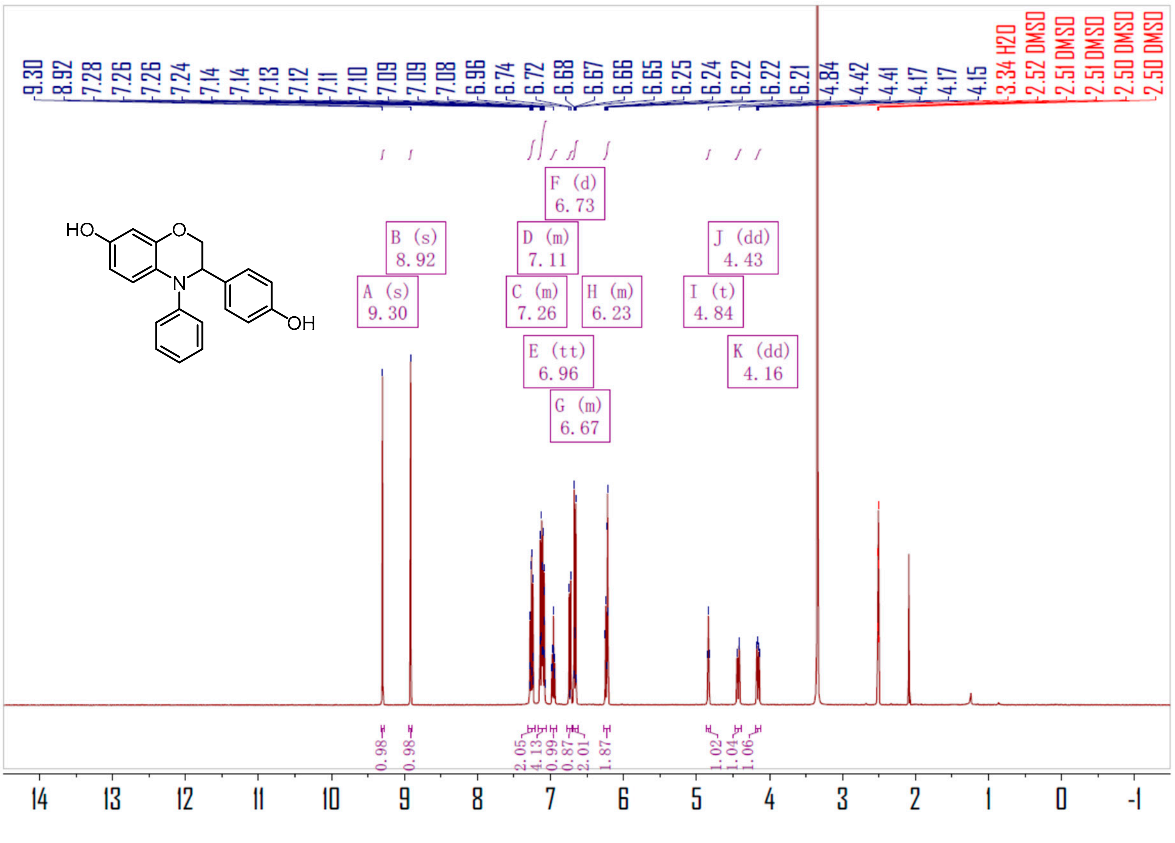

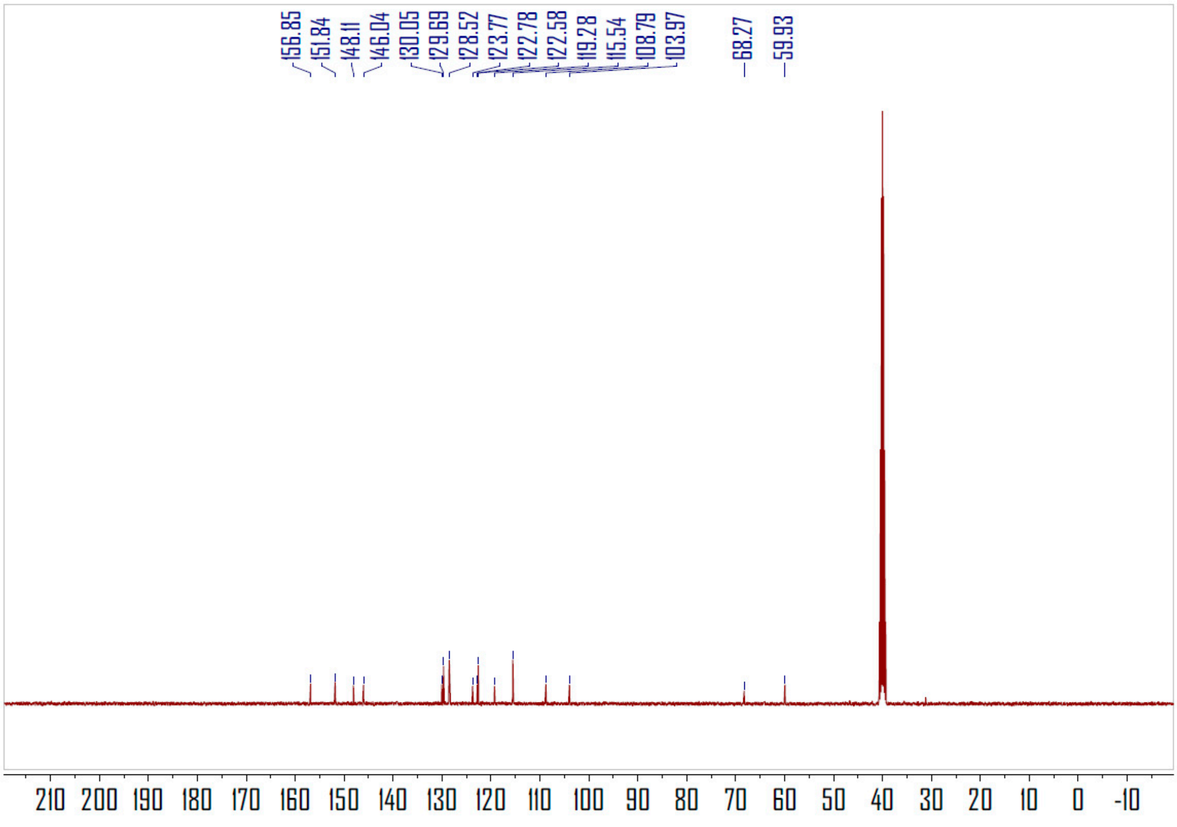

4,4'-(7-hydroxy-2,3-dihydro-4H-benzo[b][1,4]oxazine-3,4-diyl)diphenol (***14d***)

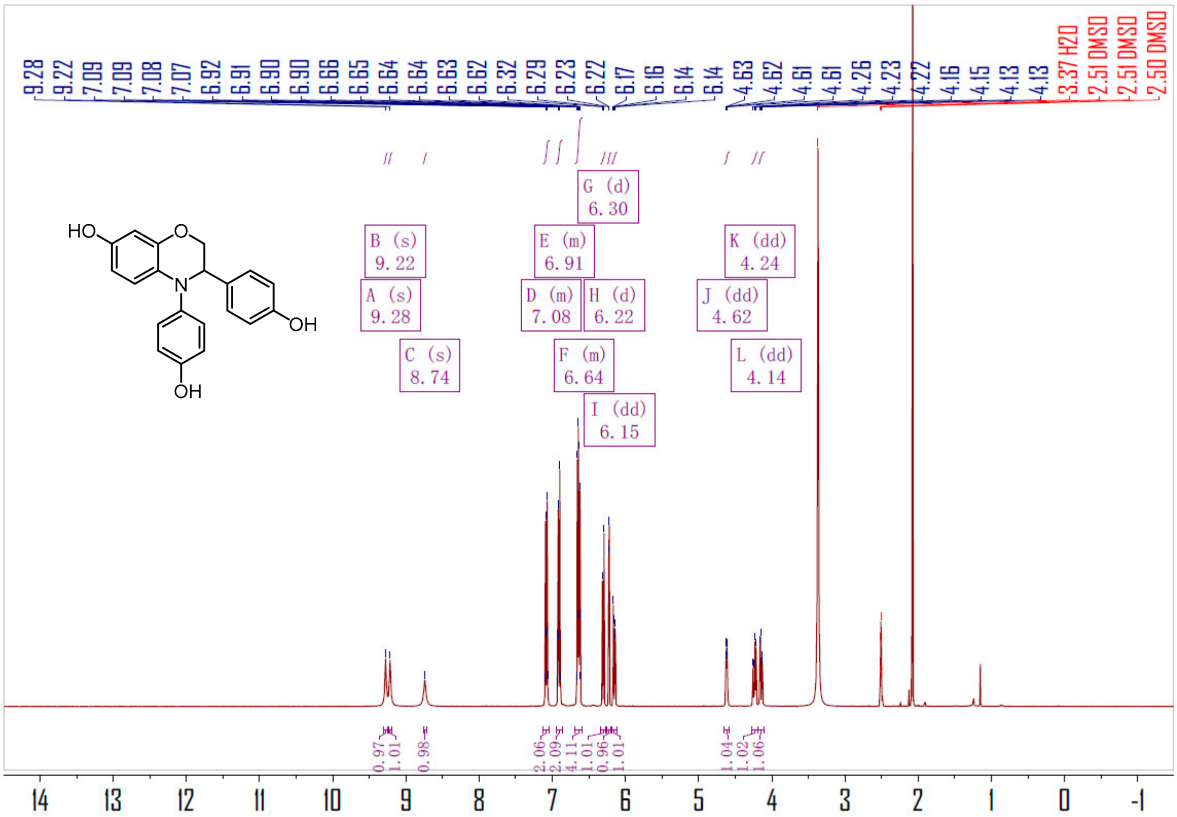

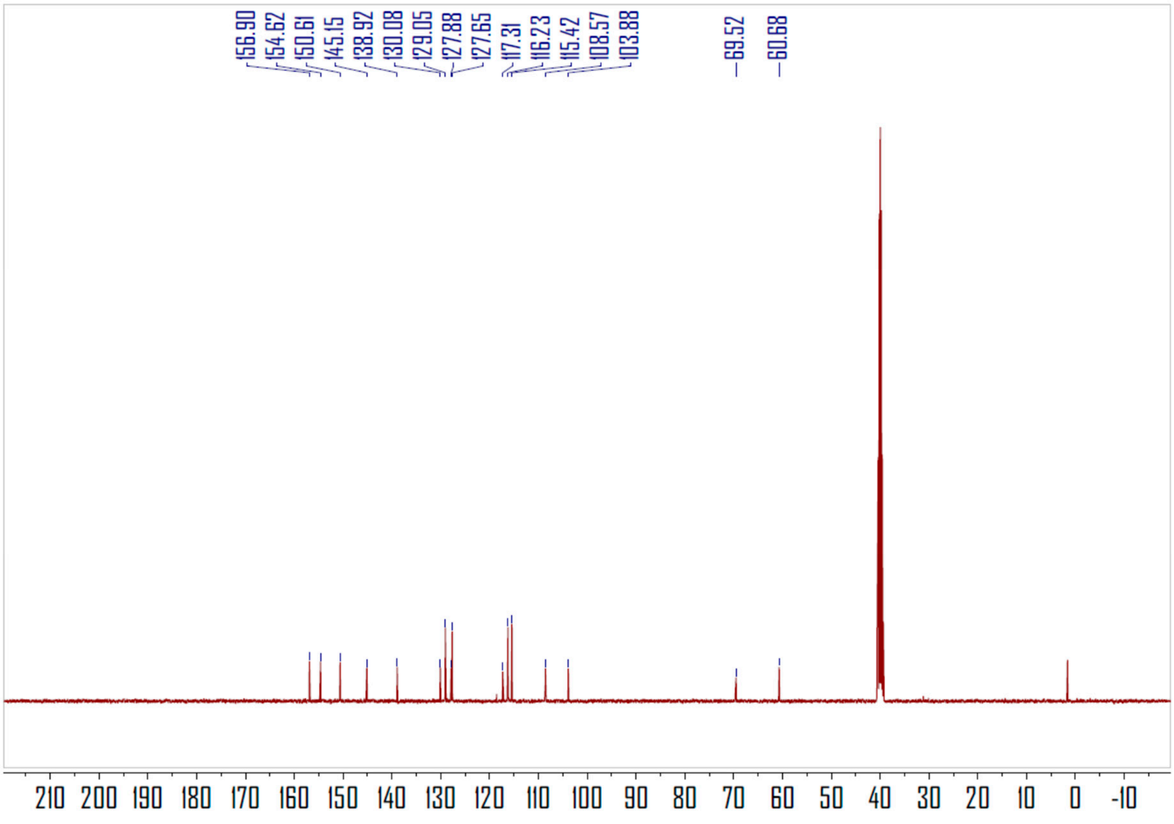

*4-(4-aminophenyl)-3-(4-hydroxyphenyl)-3,4-dihydro-2H-benzo[b][1,4]oxazin-7-ol (**14e**)*

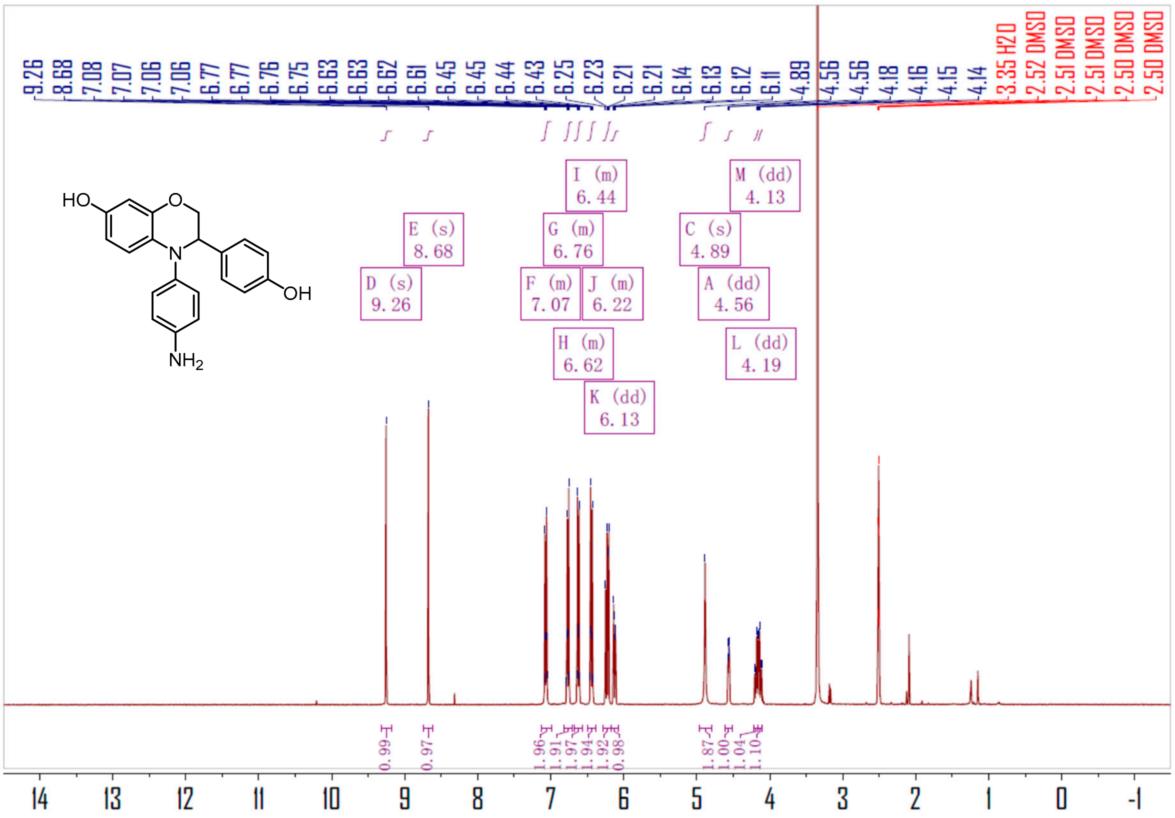

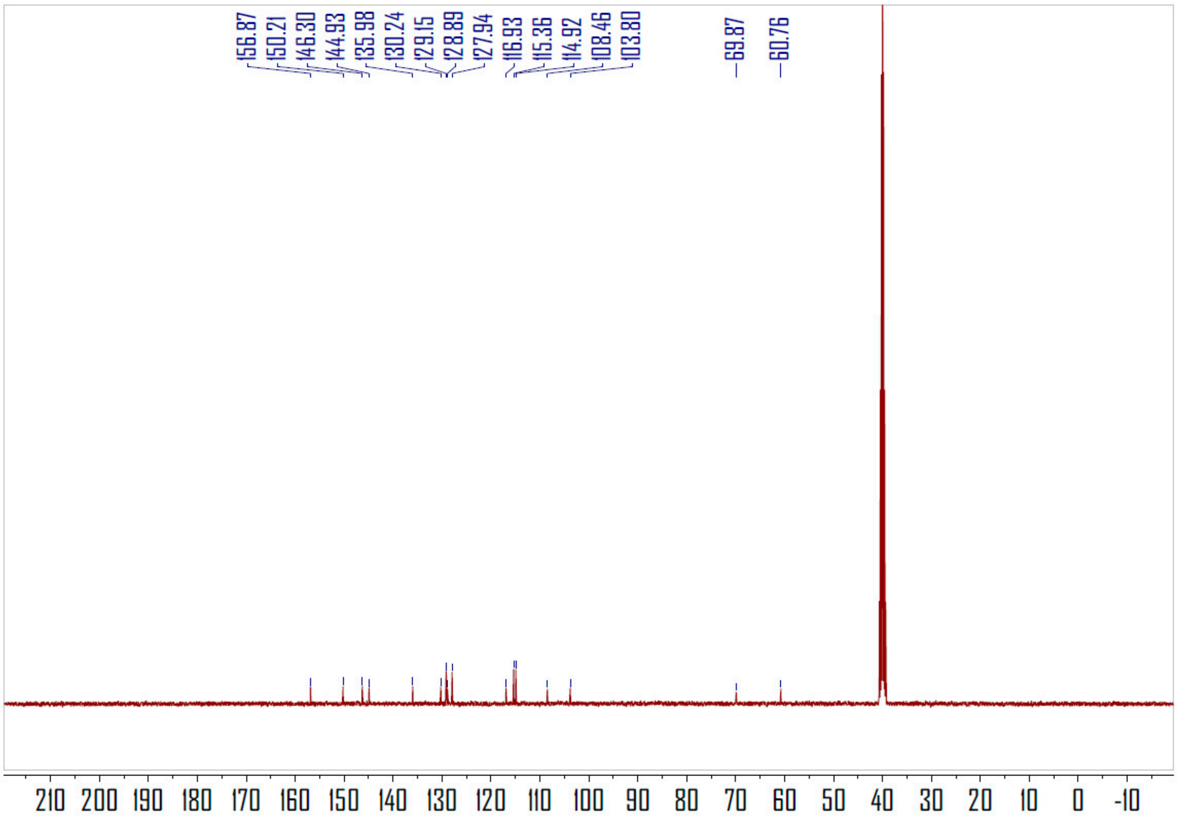

*4-(4-aminophenyl)-3-(2,4-dimethylphenyl)-3,4-dihydro-2H-benzo[b][1,4]oxazin-7-ol (**14f**)*

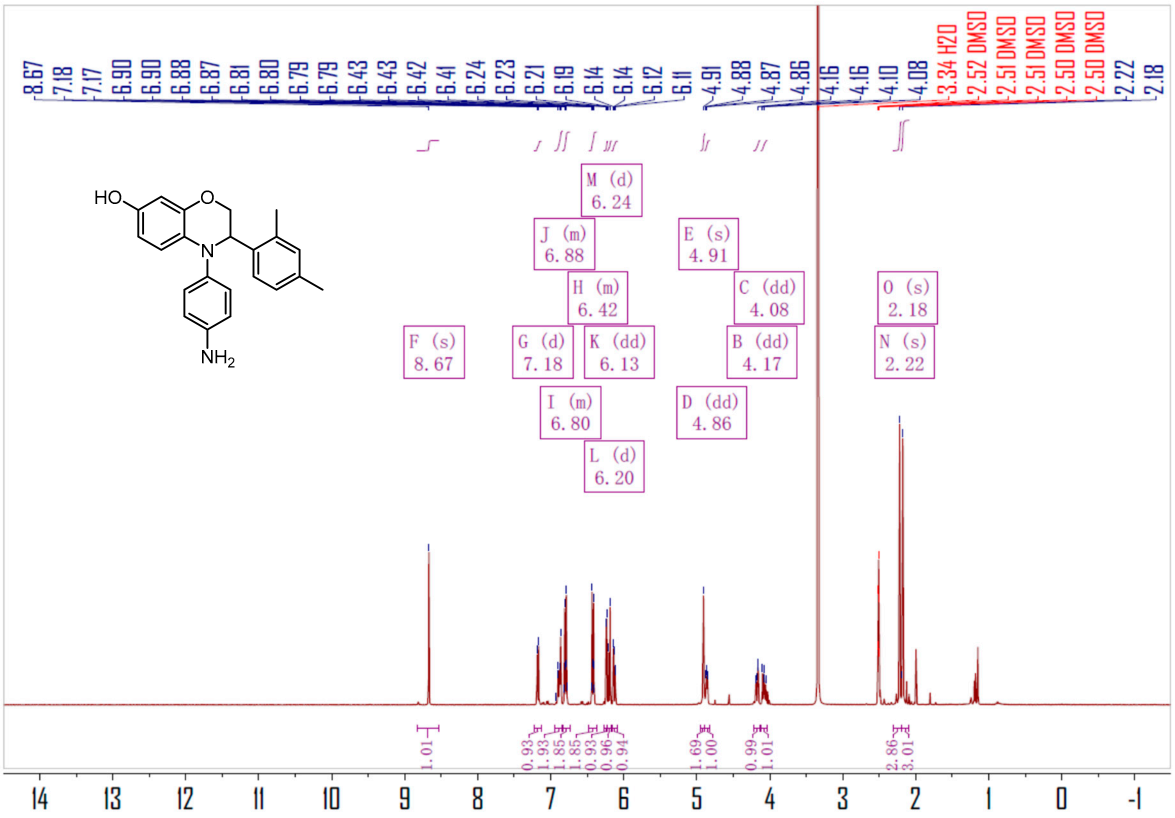

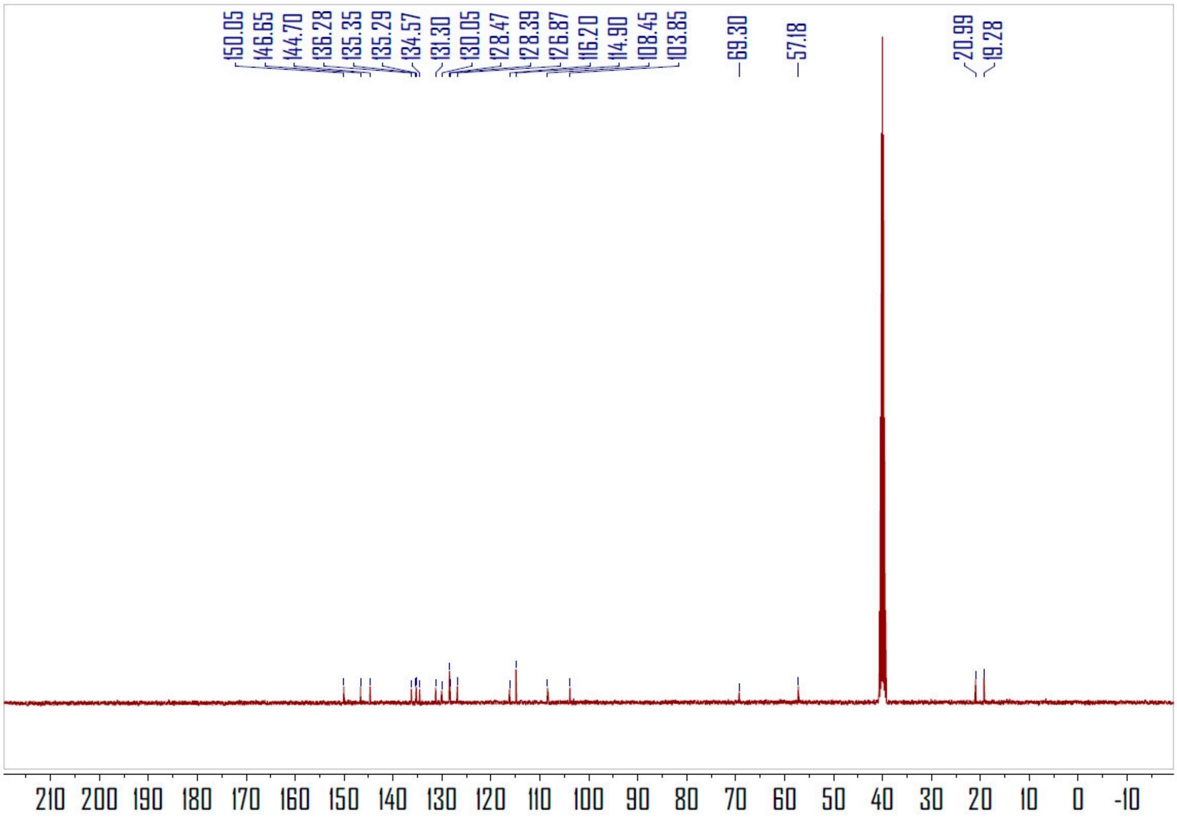

*3-(4-fluorophenyl)-4-(4-hydroxyphenyl)-3,4-dihydro-2H-benzo[b][1,4]oxazin-7-ol (**14g**)*

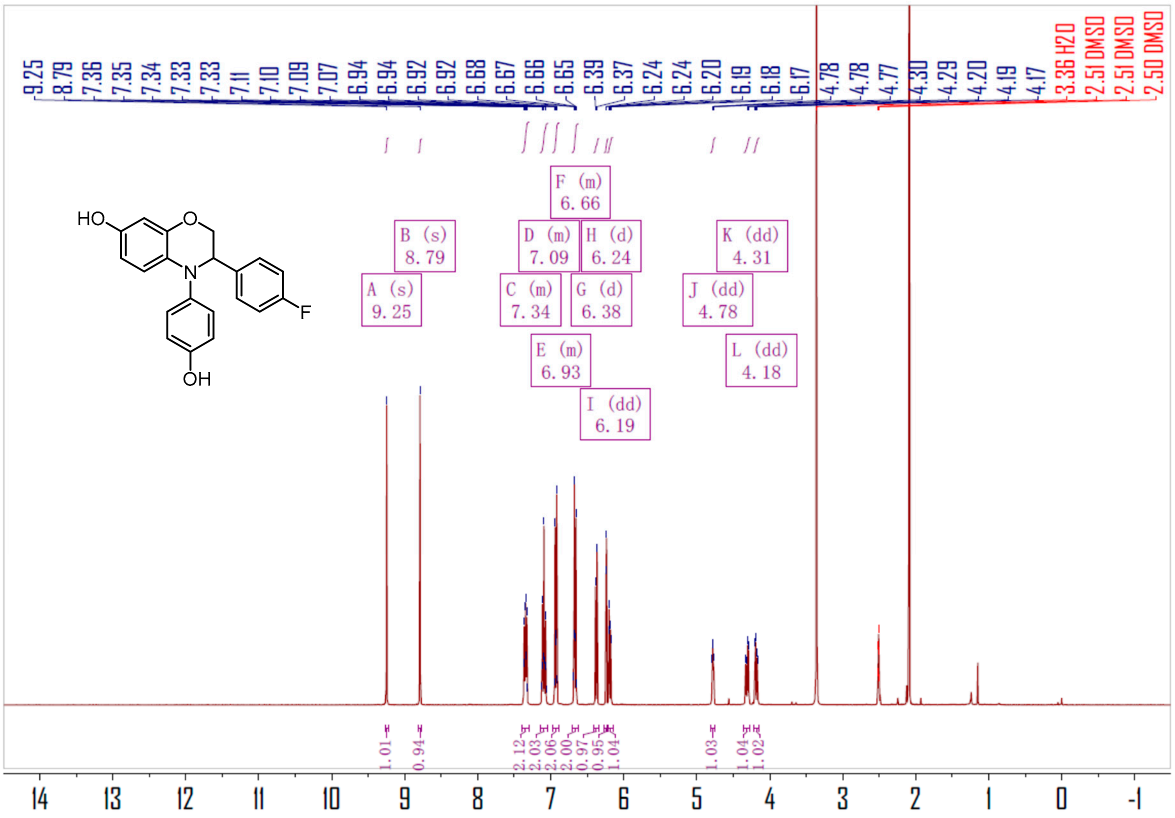

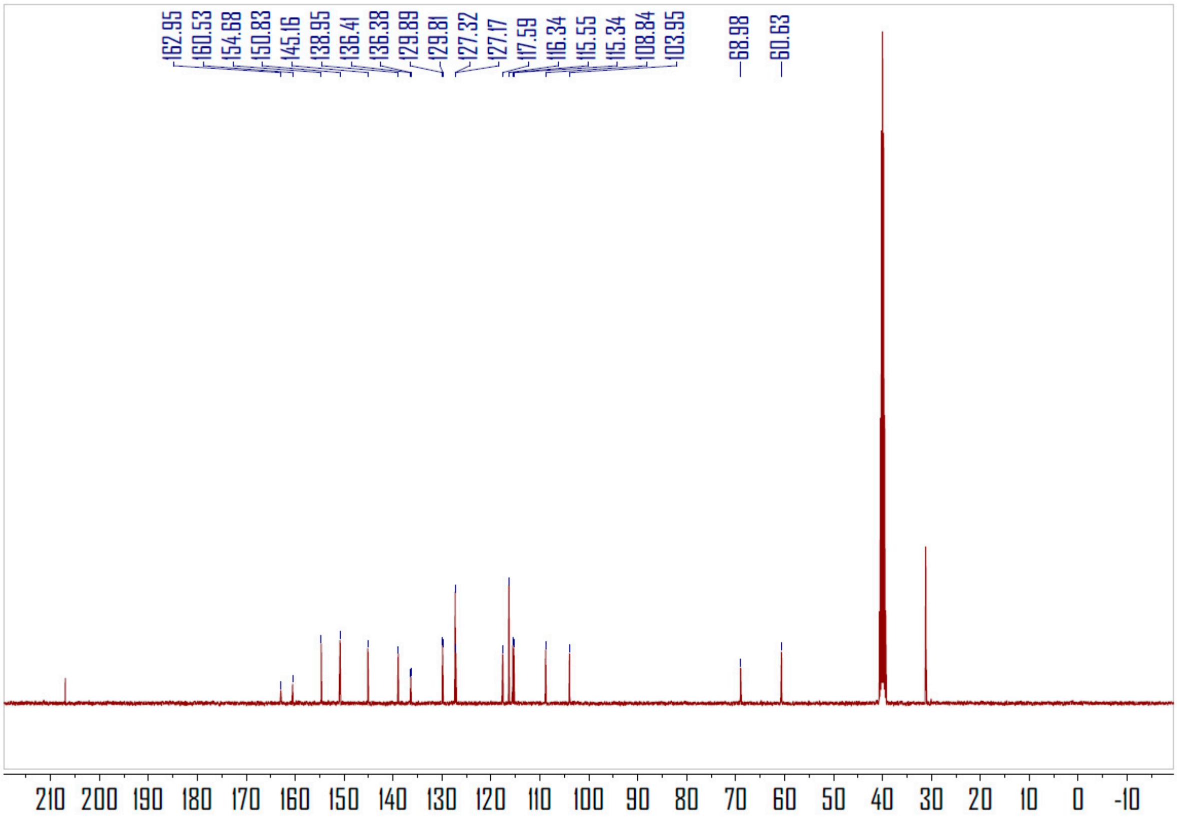

*3-(4-fluorophenyl)-4-(quinolin-3-yl)-3,4-dihydro-2H-benzo[b][1,4]oxazin-7-ol (**15c**)*

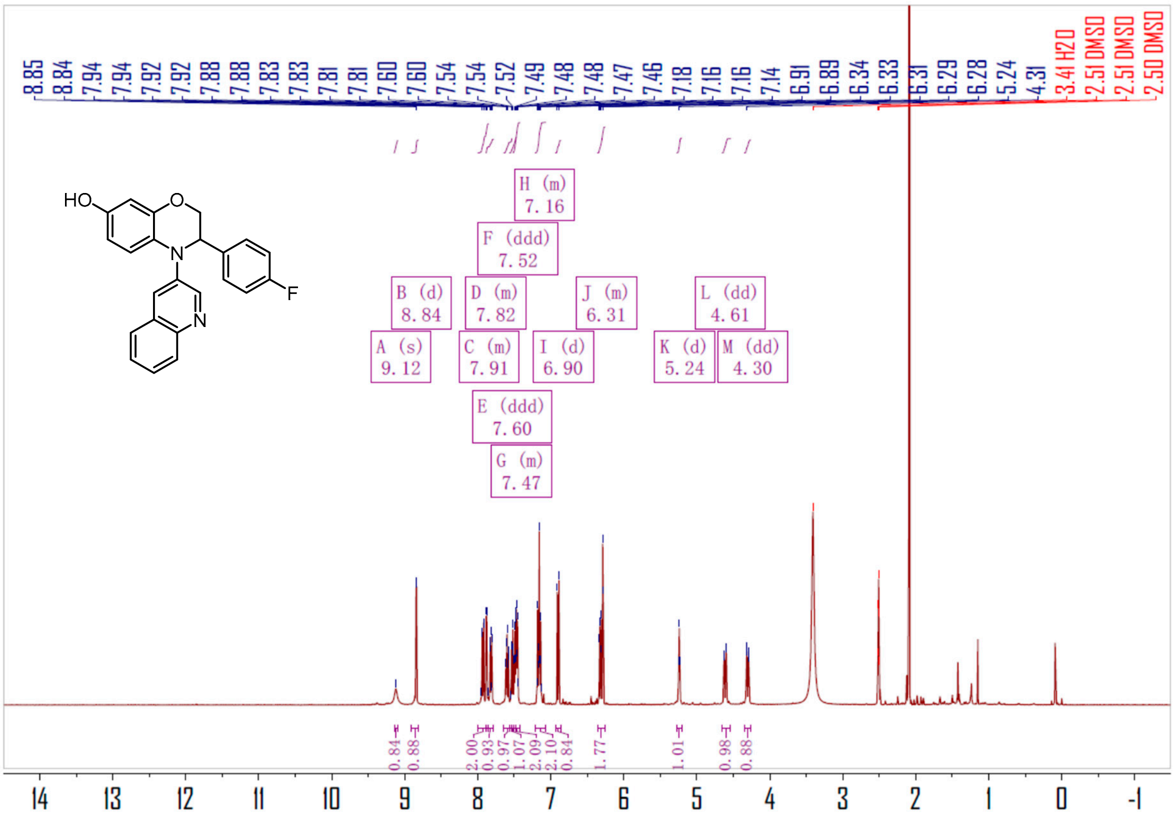

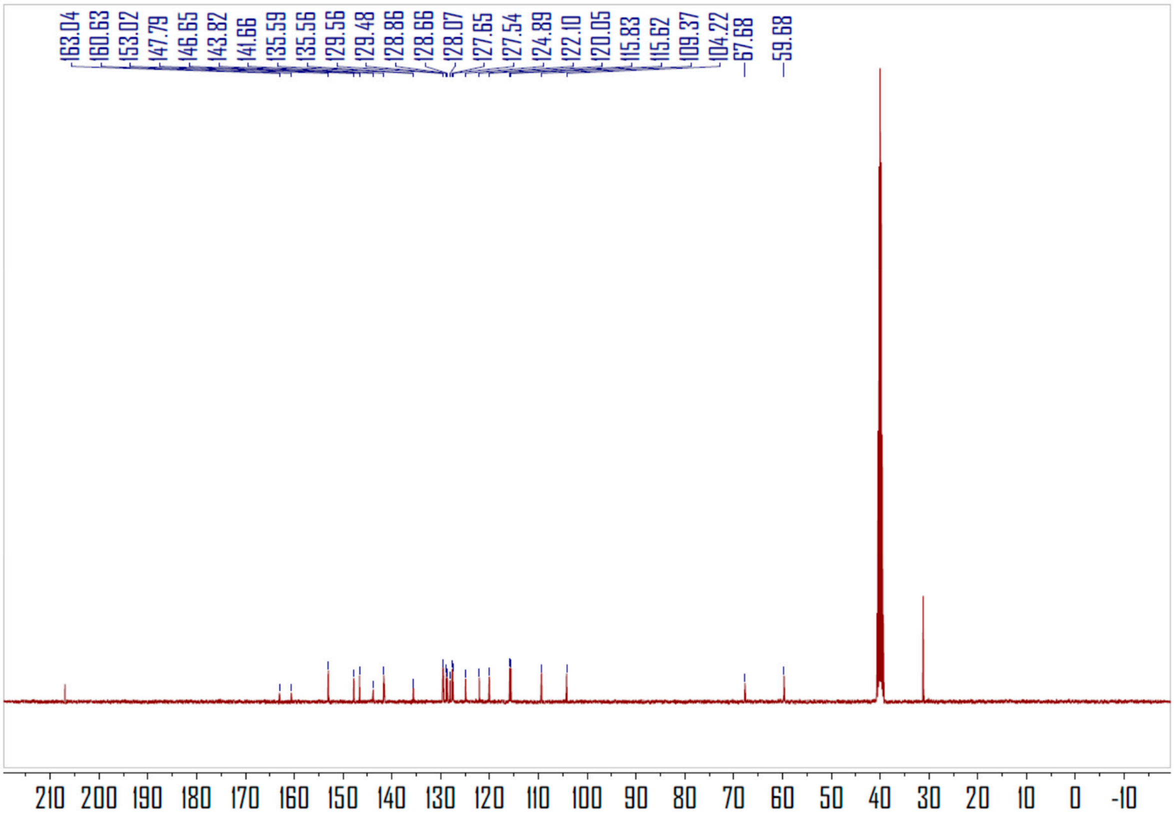

Information about the cell lines.

Name	Vendor	Cat#	Lot. No	Description	Growth properties	Complete medium	Seeding Density	Doubling Time	incubation time
PC-3	ATCC	CRL-1435	7348670	prostate, adenocarcinoma	adherent(The cells form clusters in soft agar and can be adapted to suspension growth)	F-12K+10%FBS	3200	31.37	**72h**
MIA PaCa-2	ATCC	CRL-1420	57866601	pancreas, pancreatic Carcinoma	adherent, single cells and loosely attached clusters	DMEM+10%FBS	1700	21	**72h**
MDA-MB-231	ATCC	HTB-26	4031218	breast, adenocarcinoma (pleural effusion)	adherent	Leibovitz's L-15+10%FBS	4000	**31**	**72h**
